# Integrative Transcriptomic and Systems Biology Analyses Identify *TCB1* as a Calcium-Responsive Gene in *Cryptococcus neoformans*

**DOI:** 10.3390/microorganisms14010122

**Published:** 2026-01-07

**Authors:** Andrea Gomes Tavanti, Júlia Catarina Vieira Reuwsaat, Heryk Motta, Eamim Daidrê Squizani, Rodrigo Silva Araujo Streit, Patrícia Aline Gröhs Ferrareze, Matheus da Silva Camargo, Bruno Cesar Feltes, Marilene Henning Vainstein, Charley Christian Staats, Lívia Kmetzsch

**Affiliations:** 1 Molecular Biology of Pathogens Laboratory, Center of Biotechnology, Federal University of Rio Grande do Sul, Porto Alegre 91501-970, Brazil; 2 Graduate Program in Cellular and Molecular Biology, Center of Biotechnology, Federal University of Rio Grande do Sul, Porto Alegre 91501-970, Brazil; 3 Laboratory of DNA Repair and Aging, Department of Biophysics, Institute of Biosciences, Federal University of Rio Grande do Sul, Porto Alegre 91501-970, Brazil; 4 Laboratory of Microorganisms of Medical and Biotechnological Importance, Center of Biotechnology, Federal University of Rio Grande do Sul, Porto Alegre 91501-970, Brazil; 5 Department of Molecular Biology and Biotechnology, Institute of Biosciences, Federal University of Rio Grande do Sul, Porto Alegre 91501-970, Brazil; 6 Laboratory of Molecular and Cellular Microbiology, Center of Biotechnology, Federal University of Rio Grande do Sul, Porto Alegre 91501-970, Brazil

**Keywords:** tricalbin, polysaccharide capsule, gene discovery, integrative systems biology, calcium signaling

## Abstract

*Cryptococcus neoformans* is a pathogenic yeast and the leading cause of cryptococcosis in humans. The calcium-calcineurin signaling pathway plays a central role in stress adaptation and virulence. To identify the uncharacterized regulators of fungal adaptation, we utilized an integrative systems biology approach, combining differential gene expression and network analysis using transcriptomic data from three key components of the calcium-calcineurin pathway (Cna1, Crz1, and Pmc1). Our workflow identified the *CNAG_00522* gene product, which we designated tricalbin 1 (*TCB1*) due to its conserved calcium and lipid-binding C2 domains. *TCB1* expression was found to be regulated by both Cna1 and Pmc1. Network analyses positioned Tcb1 as a bottleneck linking general stress response and cellular processes. Further molecular characterization confirmed that *TCB1* expression is temperature and calcium-responsive. Functional studies of the *tcb1*Δ mutant revealed an enlarged capsule, increased GXM shedding, and enhanced viability under host-mimicking conditions. However, phenotypic screening demonstrated that the *tcb1*Δ mutant does not display sensitivity to cell wall or osmotic stressors, and *TCB1* deletion did not attenuate virulence in the *Tenebrio* larval model. These findings suggest that *TCB1* functions as a specialized regulator of fungal growth at 37 °C, capsule size, and GXM shedding. This study validates our integrative approach for guiding the identification of these complex regulators.

## 1. Introduction

Invasive fungal infections impact millions of people globally [1]. Cryptococcosis is an acute, invasive fungal disease that primarily affects immunocompromised individuals, with cryptococcal meningitis (CM) representing its most severe manifestation [1,2,3]. The basidiomycetous yeast *Cryptococcus neoformans* is the leading etiological agent of CM and is particularly prevalent among HIV-infected individuals [3,4,5]. CM has an estimated incidence of 194,000 cases annually, with more than 147,000 associated deaths [1]. Recently, the World Health Organization (WHO) added *C. neoformans* to the fungal priority pathogens list to guide research, development, and public health action [6]. Current treatments for cryptococcosis are associated with significant side effects and limited availability in certain countries [3,7,8]. Therefore, understanding the mechanisms by which *C. neoformans* establishes the disease is crucial for developing more effective and accessible treatments [9,10,11].

Humans primarily come into contact with *C. neoformans* by inhaling desiccated yeast cells or basidiospores from environmental sources. Under favorable conditions, cryptococcal cells can evade the innate immune response and establish a lung infection [4,12,13]. Cryptococcal cells may then transmigrate via the bloodstream to the central nervous system (CNS), causing meningitis [12,13]. *C. neoformans* cells rely on a set of virulence determinants to survive and adapt to the host environment [14], and the calcium-calcineurin pathway is crucial for pathogenesis and stress adaptation [15,16,17,18,19]. Calcium is an important secondary messenger that mediates responses to several environmental stimuli [20], and appropriate calcium levels are essential to coordinate adequate stress responses. Intracellular calcium levels are controlled by the activity of several transporters, such as the vacuolar transporter Pmc1, and by channels such as Cch1-Mid1 [17,18,21,22]. Calcium is sensed by calmodulin, which activates the phosphatase calcineurin [10,20], regulating different targets that control a myriad of biological processes at both transcriptional and post-transcriptional levels [16]. The transcription factor Crz1 is one of the calcineurin targets and regulates cryptococcal adaptation to human host temperature and the cell wall integrity [23].

The core components of the calcium-calcineurin signaling cascade are conserved across eukaryotes. However, considerable variation exists in the downstream targets of this pathway and regulators that influence it among different species [10]. This signaling pathway has emerged as a promising target for the development of novel antifungal therapies [9,11,24,25,26]. In particular, species-specific elements within the calcium-calcineurin pathway are of great interest, as they may serve as selective targets for new antifungal drugs [10]. Identifying novel regulatory genes, particularly those whose mechanism may be non-canonical to the primary effectors (e.g., Crz1), remains a challenge.

In this study, we employed an integrative systems biology workflow combining differential gene expression (DEG) analysis of transcriptomic data from key *C. neoformans* calcium-calcineurin pathway mutant strains (*cna1*Δ, *crz1*Δ, and *pmc1*Δ). This systematic screening identified the uncharacterized gene product *CNAG_00522*, which we designated Tricalbin 1 (*TCB1*) based on the presence of conserved calcium and lipid-binding C2 domains and orthology with fungal tricalbins. Gene expression analyses confirmed that *TCB1* expression is thermally responsive and influenced by extracellular calcium. Furthermore, the *tcb1*Δ deletion mutant displayed increased capsule thickness, higher GXM shedding and enhanced viability in host-mimicking conditions. Overall, these results demonstrate the utility of our integrative screening approach for novel gene discovery and suggest that *TCB1* is engaged in *C. neoformans* adaptation to the environment and host conditions, functioning as a regulator within the calcium signaling network that controls specific virulence traits.

## 2. Materials and Methods

### 2.1. Differential Gene Expression Analysis (DGEA)

Studies of *C. neoformans* wild-type (WT, H99), *crz1*Δ mutant, and calcineurin (*cna1*Δ) mutant strains grown at 37 °C were obtained from NCBI’s Gene Expression Omnibus (GEO) [27] under the accession number GSE93005 [15]. Raw RNA-Seq data of H99 and the *pmc1*Δ mutant, also grown at 37 °C, were obtained from the NCBI SRA database [28,29] under accession code SRP120588 [19]. All samples were in three biological replicates, except *cna1*Δ, which is composed of two replicates.

Quality of reads in fastq files was evaluated using FastQC [30] version 0.11.5. Different packages, such as Cutadapt [31] version 1.15, Trimmomatic [32] version 0.39, Sickle [33] version 1.33, and FASTX-toolkit [34] version 0.0.14, were used for the removal of potential adaptors. The processed reads were then aligned to the *C. neoformans* H99 genome sequence (NCBI Reference GCF_000149245.1) and respective GFF file using STAR software version 2.7.6 [35], with the following parameters: “--outFilterType BySJout --alignIntronMin 10 --alignIntronMax 3000 --outFilterIntronMotifsRemoveNoncanonical --genomeDir [genome file] --runThreadN 12 --readFilesIn [fastq file] --outFileNamePrefix [output alignment file]”. MultiQC version 1.9 [36] was used to generate STAR alignment score plots (Supplementary Figure S1). The resulting SAM files were converted to BAM files using SAMtools version 1.7 [37]. Read summarization was performed using HTSeq version 0.12.4 [38], with the following parameters: “-f bam --stranded=no --type=exon --idattr=locus_tag [bam file] [gff file] > [output count file]” and DEG analysis was performed in the integrated development environment RStudio version 1.2 [39] using a built script in R language, which is available at: https://github.com/deatavanti/differential-expression-analysis-script.git(accessed on 21 January 2021). The R package DESeq2 [40] version 1.24 was utilized for the differential gene expression analyses. DEGs are defined as those having absolute values of Log_2_ fold change (FC) higher than or equal to |1| and FDR-adjusted *p*-values lower than 0.05.

### 2.2. Protein–Protein Interaction Networks (PPINs) Construction and Tcb1 Selection

DEGs lists from comparison between WT and each mutant were submitted individually to perform a PPIN assembly in the STRING database [41] version 12.0. Due to limited entries of *C. neoformans* H99 proteins in the STRING database, the construction of the PPIN was conducted using the *C. neoformans* JEC21 protein-coding genes, which are syntenic orthologs to the respective H99 DEGs. Conversion of codes was performed using the FungiDB website [42]. In STRING, basic settings were modified regarding active interaction sources: gene fusion and text mining were disabled, and the minimum required interaction score was set to “low confidence at 0.150”. The maximum number of first shell interactors was adjusted to 200. Not all DEGs connected in STRING, likely due to limited interaction evidence or network settings. This limitation excluded certain DEGs from subsequent analyses. The first shell interactor protein codes had to be converted to their corresponding gene codes (this option is not currently available on the STRING database) to enable posterior functional enrichment analysis. From STRING, a protein annotation TSV file, which contained all nodes present in the network, was exported for each network and used for code conversion in the FungiDB website. The resulting networks were also exported to TSV files, and their protein codes were converted to gene codes by using a Python (version 2.7.17) script available at: https://github.com/deatavanti/protein-to-gene-conversion-python-script.git (accessed on 23 November 2025). After this modification, the networks were imported into Cytoscape [43] version 3.10 with the “combined interaction score” used as edge attributes. Dynet plug-in [44] was used to generate an overlap map of each mutant network. The node tables were filtered to identify genes present in all three networks (Cna1, Pmc1, Crz1) or in at least two networks, as these overlap patterns suggest potential central or connector roles. The filtered node lists were analyzed in FungiDB to check for annotations and examine conserved domains, and upon target selection, we also utilized the database to verify possible orthologs and associated transcriptomic data.

### 2.3. PPINs Topological and Functional Enrichment Analysis

Upon selection of Tcb1, present in *cna1* and *pmc1* networks, cluster analysis was performed on both networks through the MCODE plug-in [45] using default parameters except enabling “include loops” with a degree cutoff of 2 and enabling “fluff” with node density cutoff of 0.1. CentiScaPe plug-in [46] was used to check the centralities (degree and betweenness) of the selected node in each network. Functional enrichment analysis was performed on clusters where Tcb1 was present using the ClueGO plug-in [47] to gain insight into which processes this protein may be involved in. The default parameters were applied except for enabling “show only pathways with *p*-value ≤ 0.05”. The selected ontologies were Gene Ontology (GO) biological processes and molecular functions [48]. ClueGO outputs were used for bubble plotting using ggplot (version 3.5.2) in the R environment (version 4.5.1) using custom scripts, which will be available upon request.

### 2.4. Conserved Domain Analysis

Conserved domain analysis of the *C. neoformans* Tcb1 protein was performed using the InterProScan tool available on the InterPro website version 106.0, using default parameters [49]. The Tcb1 protein sequence was obtained from the FungiDB database using the “Retrieve Sequence” tool (Supplementary Table S1).

### 2.5. Transcription Factor Binding Sites (TFBS) Inference

The *TCB1* predicted regulatory region, here defined as the region encompassing the 1000 nucleotides upstream of the transcription start site (TSS), was obtained using the retrieve sequence tool of the FungiDB website. Putative Crz1 and Pdr802 motifs were obtained from Chow et al. (2017) [15] and Reuwsaat et al. (2021) [50], respectively. Searches for TFBS were conducted using Find Individual Motifs Occurrences (FIMO) version 5.3.0 [51]. Crz1 and Pdr802 motifs (Supplementary Table S2) were used as motif input, and the *TCB1* promoter region (Supplementary Table S3) was used as sequence input. FIMO matches were filtered considering *p*-value < 0.0001.

### 2.6. RNA Extraction and qRT-PCR Analysis

The transcript levels of *TCB1* were assessed using qRT-PCR from RNA samples collected from the H99 strain and the null mutant for the *PMC1* gene *(pmc1*Δ), as well as from the KN99α strain and the *crz1*Δ mutant. The RNA extraction and qRT-PCR analysis were performed as previously described by Squizani et al. (2018) [19]. Briefly, yeast cells were cultured in yeast extract peptone dextrose (YPD) medium (1% yeast extract, 2% peptone, 2% dextrose) for 16 h at distinct temperatures (30 °C or 37 °C) with and without 100 mM CaCl_2_. All strains were cultivated in three biological replicates. Total RNA was extracted with Trizol (Invitrogen, Waltham, MA, USA), treated with DNase I (Promega, Madison, WI, USA), and reverse transcribed with ImProm-II (Promega). For qRT-PCR, each biological replicate was analyzed in three technical replicates for each primer pair. *ACT1* cDNA levels were used for data normalization. Relative expression was determined by the 2^−ΔCT^ method [52]. Primer sequences for the expression detection of *TCB1* and *ACT1* are listed in Supplementary Table S4.

### 2.7. Knockout Mutant Construction

The *TCB1* deletion mutant strain was constructed via the split-marker strategy [53]. Briefly, fragments of 1 kb upstream and 1 kb downstream of the *TCB1* coding region were fused via PCR to the Hygromycin resistance gene. The resulting deletion cassette was then introduced into the *C. neoformans* H99 strain via biolistic transformation, as previously described [54]. One deletion mutant for the *TCB1* gene (*tcb1*Δ) was recovered and used for phenotypic tests. RT-PCR and qRT-PCR were performed to confirm the mutant strain construction. To rule out ectopic integration of the deletion cassette, the relative copy number of the *ACT1* promoter (P*_ACT1_*) was determined via qPCR. Genomic DNA from the H99 wild-type (WT) and *tcb1*Δ strains was analyzed using standard curves with primers for *ACT1* coding sequence (CDS) and the hygromycin resistance gene promoter (P*_ACT1_*). Because *ACT1* is a single-copy gene in *C. neoformans* and its promoter is also used to drive the hygromycin cassette, a correctly constructed knockout mutant is expected to contain exactly two copies of the promoter sequence, whereas ectopic integration would yield three or more copies. The primers used for the construction and confirmation are listed in Supplementary Table S4. *C. neoformans* H99 wild-type and *tcb1*Δ mutant strains were maintained on YPD solid medium plates. For mutant strain selection, hygromycin (200 µg/mL) was added to YPD plates.

### 2.8. Capsule Induction, GXM Quantification, Phenotypic Tests, and Growth Viability

Fungal cell strains grown overnight in YPD were washed three times with phosphate-buffered saline (PBS), and cell concentrations were estimated via optical density (OD_600_). Cell suspensions were diluted to 10^6^ cells/mL and plated in three replicates for each strain in a 24-well plate containing 1 mL DMEM (D6429, Sigma Aldrich, St. Louis, MO, USA). The plates were incubated at 37 °C and 5% CO_2_ for 72 h. For the viability estimation, the colony-forming units (CFU) were determined after growth on YPD agar at 30 °C for 48 h; the inoculum of each strain was used as a control. For capsule induction analysis, cells were fixed in 4% paraformaldehyde and washed with PBS after 72 h of incubation. *C. neoformans* cells were pipetted on glass slides and mixed with a similar volume of India ink. Cells were visualized using an optical microscope, and capsule measurements were performed using the ImageJ software (version 1.54g). A total of 150 cells were measured for each strain (50 cells per biological replicate).

For the GXM quantification, cells were grown in DMEM for 48 h as described above. The cell-free supernatant was used for GXM quantification via ELISA, using anti-GXM antibody as previously described [55,56]. For the phenotypic tests, 3 μL of 10-fold serial dilutions (10^7^ to 10^3^ cells/mL) were spotted on YPD agar with different stressors (NaCl, CaCl_2_, Congo Red, Calcofluor White, Caffeine, SDS, Ethanol, Fluconazole, Caspofungin, pH 8.0 and FK506), and YNB at acidic and neutral pHs (4.0 and 7.0), at concentrations indicated in the figure.

### 2.9. Virulence Assay Using Tenebrio Molitor

Fungal cells were initially cultured in YPD medium for 16 h at 30 °C with agitation. Subsequently, 1 mL was transferred to fresh YPD medium and cultured for an additional 16 h under the same conditions. The fungal cells were then washed three times with PBS and diluted. Cell concentrations were estimated using a Neubauer chamber, and cell suspensions were diluted to a concentration of 2 × 10^4^ cells/μL. A total of 20 larvae were inoculated with 5 μL of the diluted cell suspension for each of the fungal strains. Additionally, 20 larvae were inoculated with 5 μL of PBS, as a negative control. The larvae were incubated in sterile Petri dishes at 37 °C. Larval mortality was monitored and recorded every 24 h until there were no survivors remaining from the mutant and wild-type strain infections. The experiment was repeated with an initial inoculum concentration of 2 × 10^3^ cells/μL. Kaplan–Meier survival analysis was performed for statistical analysis using GraphPad Prism version 8 software.

## 3. Results

### 3.1. An Integrative Systems Biology Workflow Identifies TCB1 as a Novel Regulatory Node

**Figure 1:**
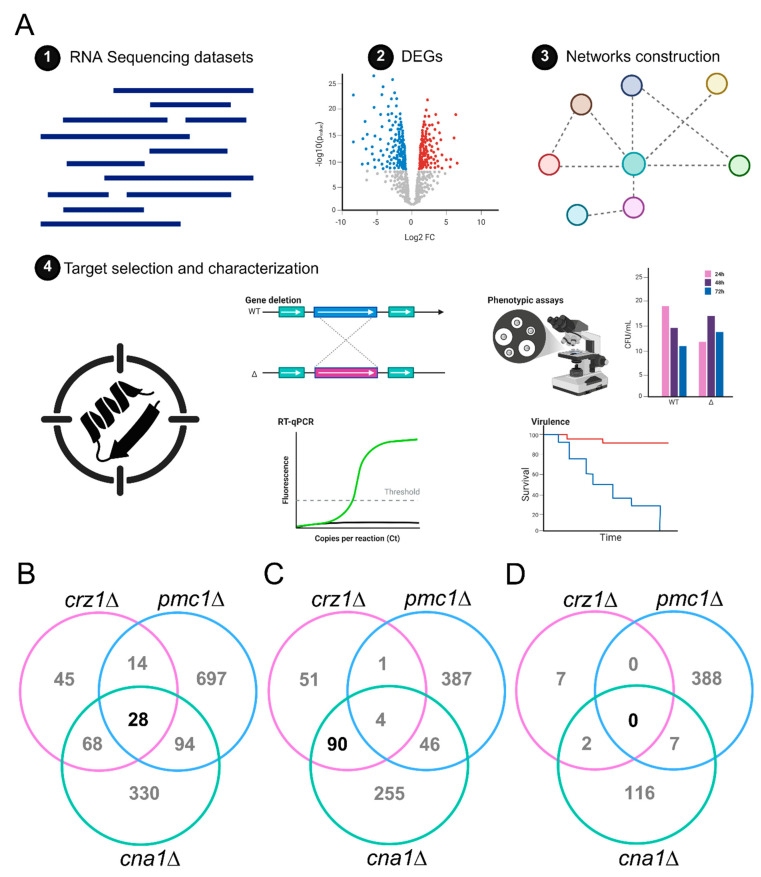
Integrative transcriptomic and network analysis for identification of cryptococcal uncharacterized genes. (**A**) Workflow employed to select uncharacterized genes associated with the calcium signaling network in *C. neoformans*. The process comprises four main steps: (1) Selection of published RNA-Seq datasets of deletion mutants from the calcineurin signaling pathway (*cna1*Δ, *crz1*Δ, and *pmc1*Δ); (2) Identification of differentially expressed genes (DEGs); (3) Construction of protein–protein interaction networks, using STRING, FungiDB, and Cytoscape; (4) Target selection and characterization, through conserved domains search, gene expression analysis, knockout mutant construction, and phenotypic and virulence assays. Venn diagram comparing the total number of DEGs (**B**), positively regulated DEGS (**C**), and negatively regulated DEGs (**D**) for *cna1*Δ, *crz1*Δ, and *pmc1*Δ related to the wild-type strain (H99). In black are the numbers of genes coregulated in the three mutants (**B**); positively regulated by both Cna1 and Crz1 (**C**); and negatively regulated by the three mutants (**D**). Illustration created in BioRender.

We employed an integrative workflow combining DEG analysis and network mapping to identify uncharacterized genes regulated by components of the *C. neoformans* calcium signaling system (Figure 1A). First, we analyzed previously published RNA-seq libraries from wild-type (*C. neoformans* H99) and three key pathway deletion mutants: *cna1*Δ, *crz1*Δ, and *pmc1*Δ. DEG analysis identified 520, 155, and 833 DEGs influenced by the absence of Cna1, Crz1, and Pmc1, respectively (Supplementary Table S5).

We observed a slight overlap of regulated genes among the *cna1*Δ, *crz1*Δ, and *pmc1*Δ mutants (Figure 1B). Notably, nearly 60% of the genes positively regulated by Crz1 were also positively regulated by Cna1 (Figure 1C). However, the overlap among the three DEGs datasets was minimal, with no negatively DEGs co-regulated comparing all datasets (Figure 1D), suggesting that a large proportion of regulated genes represent non-canonical or pathway-specific responses.

To identify relevant uncharacterized candidates, we constructed protein–protein interaction networks (PPINs) from the DEG datasets (Supplementary Figures S2–S4). This approach expanded the regulatory landscape by incorporating proteins with evidence of interaction in STRING. Overlapping the PPINs with Dynet highlighted common genes across networks. By cross-referencing these candidates with FungiDB annotations, we refined our selection. We prioritized genes based on three criteria: (1) functional novelty (hypothetical/unknown annotation), (2) presence of domains linked to calcium signaling, and (3) co-regulation across multiple PPINs (Supplementary Table S6). We first analyzed the 27 genes present across all networks, but none met all the established criteria. We then focused on genes present in two networks (Cna1 and Pmc1; Pmc1 and Crz1; or Cna1 and Crz1). From this analysis, the *CNAG_00522* gene product emerged as the most compelling candidate, appearing in both the Cna1 and Pmc1 networks. All PPINs Cytoscape sessions can be found as Supplementary Files S1–S3.

**Figure 2:**
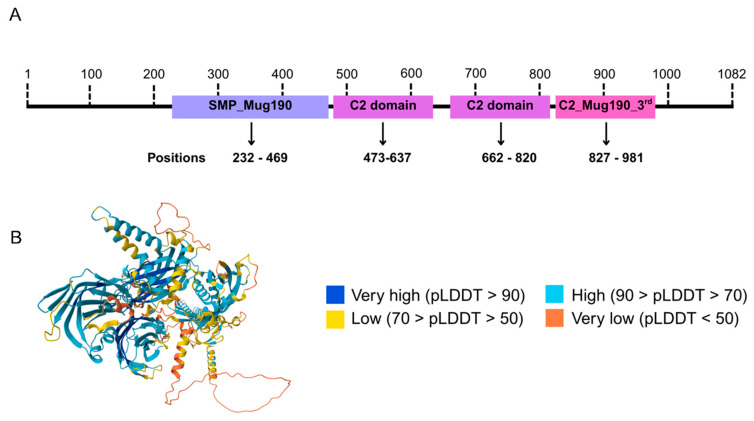
Primary and tertiary Tcb1 protein structure representations. (**A**). Schematic representation of conserved domains identified in the *C. neoformans* Tcb1 protein using InterProScan. Four domains were detected: one SMP_Mug190 domain, spanning residues 232–469, and three C2 domains. The first C2 domain extends from residues 473 to 637, the second from 662 to 820, and the third from 827 to 981. (**B**). Tridimensional representation of *C. neoformans* Tcb1 protein according to AlphaFold. Colors in protein structure indicate the per-residue model confidence score (pLDDT) between 0 and 100 generated by AlphaFold.

We designated this protein tricalbin 1 (Tcb1). Its sequence comprises one synaptotagmin-like mitochondrial lipid-binding (SMP) domain and three C2 domains (Figure 2A). The C2 domains are particularly noteworthy as they are associated with Ca^2+^-dependent membrane targeting and signal transduction [57,58]. Structural prediction also suggests the presence of these domains (Figure 2B). These features are consistent with the domain architecture observed in tricalbin orthologs from other fungi.

**Figure 3:**
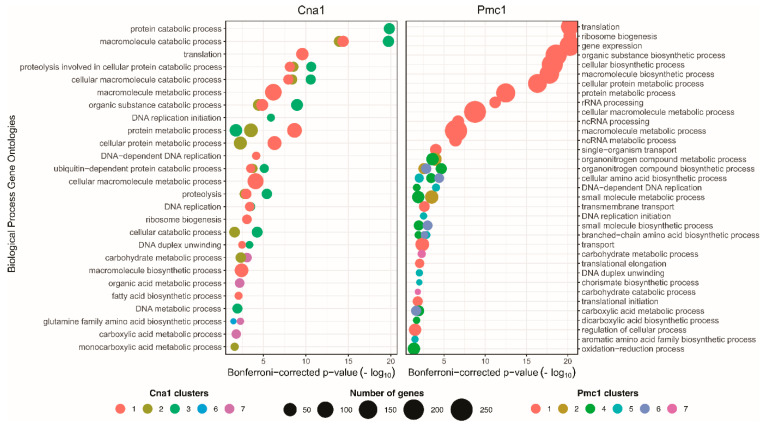
Combined functional enrichment analysis of Tcb1-containing clusters within the Cna1 and Pmc1 networks. Functional enrichment analysis of clusters containing Tcb1 within the Cna1 (**left panel**) and Pmc1 (**right panel**) protein–protein interaction networks. The bubble plots show enriched Gene Ontology Biological Process terms associated with Tcb1-containing clusters in each network. GO terms are displayed on the *Y*-axis. The *X*-axis displays the −log_10_ of the Bonferroni-corrected *p*-values for the enrichment. Bubble color indicates the specific cluster number within each network, and the size of the bubble represents the number of genes associated with each enriched GO term.

Centrality 46 Centrality analysis revealed that the Tcb1 (ortholog of *C. neoformans* JEC21 *CNA05040*) is a bottleneck in the Cna1 and Pmc1 PPINs. Further investigation of representative pathways by functional enrichment analysis from both networks demonstrated that Tcb1-containing clusters are associated with a diverse set of basal processes, including DNA replication, translation, and carbohydrate metabolism (Figure 3). The consolidation of these results strongly suggests a central, pleiotropic regulatory role for Tcb1, acting as an important connector within the overall networks.

### 3.2. External Transcriptomic Data Link TCB1 to Environmental Adaptation and Host Interaction

To gain further insight into the role of *TCB1* in cryptococcal survival and fitness, we compiled transcriptomic datasets reporting its differential expression under distinct conditions (Supplementary Table S7). These data suggested that *TCB1* may be important for survival in host-related environments [59,60,61,62]. This gene is upregulated in *C. neoformans* cells under conditions that induce titan cell formation, in cells phagocytosed by the amoeba *Acanthamoeba castellanii*, and in cells recovered from cerebrospinal fluid (CSF) of infected patients [59,60,62]. Furthermore, *TCB1* expression is linked to other key regulators. It was identified as differentially expressed in a *CCR4* knockout mutant strain (*ccr4*Δ)*,* which affects thermal stress response [61]. *TCB1* was also found to be positively regulated by Ada2 and Bzp4, transcription factors known to influence capsule formation [63] (Supplementary Table S7).

### 3.3. TCB1 Expression Is Controlled by Calcium Signaling and Core Regulators

**Figure 4:**
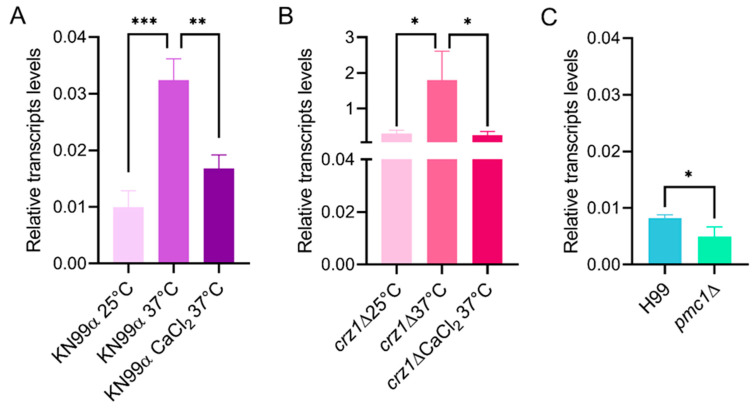
*TCB1* expression is temperature-regulated in *C. neoformans*. Transcript levels of *TCB1* were assessed by qRT-PCR in the KN99α strain (**A**), *crz1*Δ (**B**) samples at 25 °C or 37 °C with or without CaCl_2_ addition, and in H99 and *pmc1*Δ samples at 30 °C (**C**). Error bars indicate standard deviation. Statistical analysis was conducted using one-way analysis of variance (ANOVA) or Student’s *t*-test. * *p* < 0.05; ** *p* < 0.01; *** *p* < 0.001.

To validate *TCB1* as a potential component of the calcium-calcineurin pathway, we performed qRT-PCR analysis to assess its expression under a condition that activates this signaling cascade (37 °C in the presence or absence of Ca^2+^), and in *pmc1*Δ and *crz1*Δ mutants. The relative transcript levels of *TCB1* were increased in KN99α wild-type cells cultured at 37 °C compared to 25 °C (Figure 4A). The addition of Ca^2+^ led to decreased *TCB1* transcript levels compared to those observed in control conditions (Figure 4A). The same pattern was observed for mutant cells lacking *CRZ1* (Figure 4B), and we also found that proper expression of *TCB1* depends upon the presence of the calcium transporter Pmc1 (Figure 4C).

**Figure 5:**
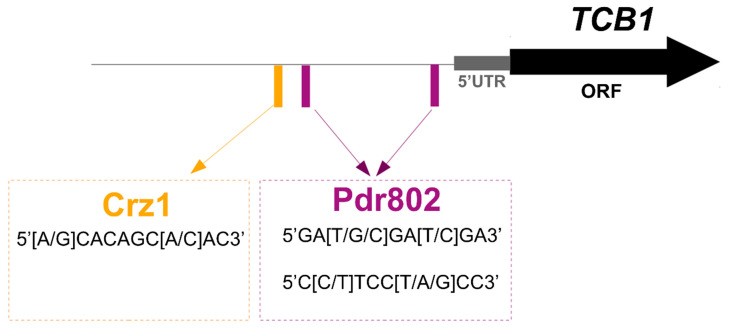
*TCB1* promoter region and transcription factor binding sites of Crz1 and Pdr802. The black line represents the *TCB1* promoter region. Orange and purple rectangles represent Crz1 and Pdr802 binding sites, respectively.

These expression patterns and dependency profiles prompted us to evaluate the presence of canonical sequences in the regulatory region of *TCB1* that could indicate a direct regulation by Crz1 and/or other transcription factors. *TCB1* promoter analysis identified putative binding sites for the transcription factors Crz1 and Pdr802 (Figure 5 and Supplementary Table S8). Crz1 is a central component of the calcium-calcineurin signaling pathway and directly regulates genes related to stress response and virulence [23,64,65,66,67]. Pdr802 is a transcription factor that negatively regulates Titan cell formation and is also essential for *C. neoformans* virulence. Direct targets of Pdr802 include the calcineurin targets Had1 and Pmc1, and *pdr802*Δ null mutant cells accumulate intracellular calcium [50]. These findings reinforce a possible participation of *TCB1* in the calcium-calcineurin signaling pathway.

### 3.4. The TCB1 Deletion Modulates Specific Virulence Traits and Exhibits a Specialized Phenotypic Profile

To further investigate the role of *TCB1* in *C. neoformans* stress adaptation and virulence, we generated a *tcb1*Δ knockout strain, in which the absence of *TCB1* transcripts was confirmed by RT-PCR and qRT-PCR, and the absence of ectopic integration of the deletion cassette was confirmed by qPCR (Supplementary Figure S5). We analyzed the *tcb1*Δ mutant for general stress susceptibility against a panel of stressors. The *tcb1*Δ mutant demonstrated no significant difference from the wild-type strain in growth assays performed at different temperatures, in the presence of cell wall stressors (e.g., Congo Red, Calcofluor White), osmotic stressors (e.g., NaCl), or antifungal drugs (e.g., fluconazole, FK506) (Supplementary Figure S6A–D). The absence of broad stress sensitivity indicates that *TCB1* operates in a specialized regulatory pathway rather than in a global stress response.

**Figure 6:**
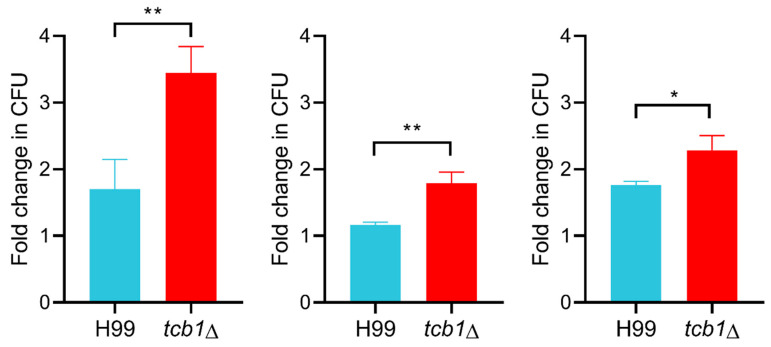
*TCB1* deletion increases cryptococcal viability in DMEM. Graphs show three independent viability estimations via CFU counts of wild-type H99 and *tcb1*Δ samples grown in DMEM media at 37 °C and 5% CO_2_ for 72 h. Statistical analysis was conducted using Student’s *t*-test. * *p* < 0.05 and ** *p* < 0.01.

**Figure 7:**
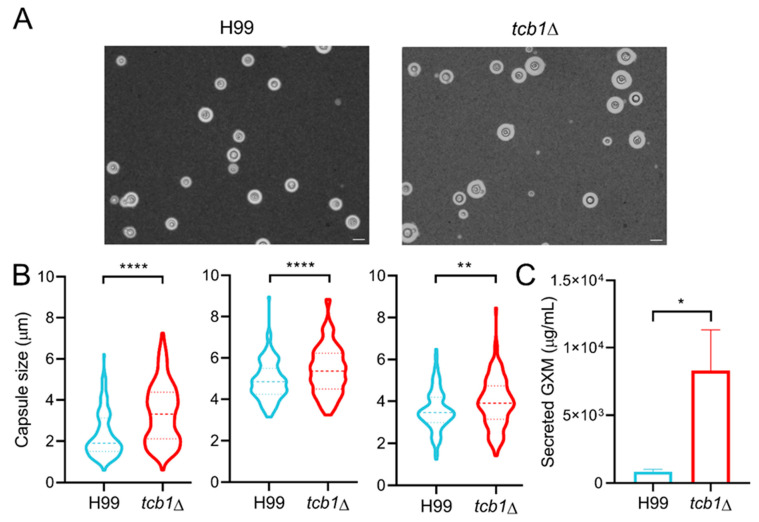
*TCB1* deletion promotes capsule enlargement and increases GXM shedding. (**A**). India ink staining to visualize the capsule of the indicated strains after growth in DMEM (at 37 °C, 5% CO_2_) for 72 h. Scale bars = 10 µm. (**B**). Graphs show the results of three independent capsule induction assays. Capsule size (µm) for wild-type H99 is compared to *tcb1*Δ strain. A total of 150 cells were measured for each strain (50 cells per biological replicate). Statistical analysis was conducted using the Mann–Whitney test. ** *p* < 0.01 and **** *p* < 0.0001. (**C**). Secreted GXM was measured after 48 h of growth in DMEM (at 37 °C, 5% CO_2_). The GXM was quantified in the cell-free supernatant via ELISA, using the anti-GXM antibody 18B7. Statistical analysis was conducted using Student’s *t*-test. * *p* < 0.05.

Despite lacking general stress susceptibility, the *tcb1*Δ mutant did exhibit altered virulence-associated traits in vitro. Based on our expression analysis and the presence of Crz1 and Pdr802 DNA-binding motifs on the *TCB1* promoter region, we evaluated the role of *TCB1* in survival under host-mimicking conditions. The fungal viability in DMEM at 37 °C and 5% CO_2_, condition that replicate the host environment, was assessed by comparing CFU counts between the mutant and wild-type strain. Interestingly, the *tcb1*Δ mutant exhibited increased viability compared to the wild-type (Figure 6). Given that the polysaccharide capsule is a major virulence factor of *C. neoformans*, we also performed a capsule induction assay. The mutant strain displayed an enlarged capsule compared to the wild-type, and secreted more GXM to the supernatant (Figure 7).

To determine the biological relevance of the observed in vitro phenotypes, we assessed *tcb1*Δ virulence in the *T. molitor* invertebrate model. The survival curve for the *tcb1*Δ mutant did not differ significantly from the wild-type control (Supplementary Figure S7), supporting the conclusion that *TCB1* has a specific, non-essential regulatory function over select virulence traits rather than a global role in pathogenesis.

## 4. Discussion

The study of complex biological systems, such as those governing fungal pathogenesis, is advanced by the rise of multi-omics data and system biology approaches [68,69]. In this context, the integrative workflow employed in this study provides a valuable method to systematically identify previously uncharacterized regulators within intricate fungal signaling systems. As a direct result of our workflow, we selected the *CNAG_00522* gene, designated as *TCB1*, a *C. neoformans* tricalbin, as a potential novel and influential component within the broad cryptococcal calcium signaling network. The function of tricalbins has been explored in the Ascomycota fungi *Saccharomyces cerevisiae* and *Candida albicans* [70,71,72,73]. In *S. cerevisiae*, tricalbins have been implicated in vacuolar morphology, glucose metabolism, and both calcium-dependent and calcium-independent lipid transport, related to plasma membrane integrity [70,71,72]. In the human pathogen *C. albicans*, tricalbin knockout strains were more sensitive to cell wall stress induced by caspofungin. They also exhibited reduced proteinase transport and attenuated virulence in mice [73]. These findings in other fungi suggest that tricalbins, including the putative *C. neoformans* ortholog Tcb1, may similarly contribute to stress responses and virulence-related processes.

The host environment challenges yeast survival, prompting the regulation of myriad biological processes related to stress and metabolism that directly impact virulence and adaptation [13]. The functional enrichment terms associated with Tcb1-containing clusters highlighted a diversity of basal processes essential to *C. neoformans’* cell maintenance, survival within the host, and pathogenicity. This result is supported by the roles described for tricalbins in other fungi species [70,71,72,73]. Crucially, centrality analysis showed that Tcb1 is a bottleneck in both Cna1 and Pmc1 networks. Bottleneck proteins represent important connectors that may have an essential role in a network [74]. The fact that Tcb1 also clustered with proteins involved in carbohydrate metabolic processes within the *C. neoformans* Co-Expression Network (CryptoCEN) further supports the hypothesis that Tcb1 acts as a key connector in essential processes enabling *C. neoformans* to adapt to various stresses and the host environment [75].

The molecular data strongly support that the *TCB1* gene responds to calcium dynamics. Considering the conserved domains identified within the *TCB1* coding sequence, together with our results showing increased *TCB1* expression at 37 °C and reduced expression in the presence of calcium, it is clear that *TCB1* transcription is responsive to Ca^2+^ signals. The protein structure includes C2 and SMP domains, which are associated with Ca^2+^ detection and lipid-dependent membrane targeting [57,58]. *TCB1* expression also depends on the vacuolar calcium transporter Pmc1, thereby directly corroborating the initial linkage suggested by the differential gene expression analysis. Pmc1 is a known participant in the calcium-calcineurin pathway, and the deletion of the *PMC1* coding gene impairs CNS dissemination and capsule formation [18,19]. The influence of Pmc1 on *TCB1* expression provides further support that *TCB1* is responsive to calcium ions.

While the preliminary genetic screening aligned *TCB1* with the calcineurin axis, our subsequent phenotypic analyses established a specialized regulatory role that differentiates *TCB1* from canonical core pathway effectors. Mutants lacking the primary calcineurin phosphatase Cna1 or the Crz1 transcription factor typically display broad hypersensitivity to cell wall, thermal, and osmotic stressors [23]. In contrast, the *tcb1*Δ deletion mutant showed no significant susceptibility defects in these standard stress assays. This evidence indicates that the Tcb1 role differs phenotypically from that of the primary calcineurin-Crz1 stress effectors.

Instead, *TCB1* appears to regulate specific cryptococcal virulence traits. *TCB1* influences capsule size, a phenotype often linked to calcium homeostasis and GXM secretion [17,18], the major component of the cryptococcal capsule during infection, which plays immunoregulatory roles in the host [76,77,78]. In our capsule induction assay, the *tcb1*Δ mutant displayed a significantly enlarged capsule compared to the wild-type strain. Additionally, the GXM secretion assay in DMEM revealed that the *tcb1*Δ mutant sheds a higher amount of GXM compared to the wild type strain at 48 h of incubation period. These findings suggest a potential link between the *TCB1* function with calcium signaling, capsule formation, and GXM production.

This modulatory role is supported by extensive external data. *TCB1* expression is upregulated when internalized by the protozoan *A. castellanii*, a natural predator of *C. neoformans* in the environment, and in titan cells inducing conditions, a morphotype that enhances resistance to host conditions and contributes to *C. neoformans* virulence [60,62]. Additionally, *TCB1* shows increased expression in CSF during infection, highlighting a role in host adaptation [59]. Its expression is negatively influenced by the Ccr4 deadenylase, which suggests *TCB1* is regulated during the switch to thermal stress adaptation-related mRNA translation [61]. Furthermore, the positive regulation of *TCB1* by the capsule-influencing transcription factors Ada2 and Bzp4 [63] is consistent with our finding that the *tcb1*Δ mutant forms a larger capsule and sheds more GXM compared to the wild-type strain. Collectively, these transcriptomic data suggest that *TCB1* may play a crucial role in fungal survival and adaptation in both host and environmental conditions [59,60,61,62,63].

The *TCB1* promoter region contains putative binding sites for the transcription factors Crz1 and Pdr802, further indicating a potential role in stress response and survival in *C. neoformans* [10,16,50]. While knockout mutant strains lacking the *PDR802* gene grow poorly in DMEM at 37 °C and 5% CO_2_ [50], the *tcb1*Δ deletion mutant displays enhanced viability in the same host-like condition. However, similar to the *pdr802*Δ mutant, the *tcb1*Δ mutant also produces a thicker capsule. These contrasting and overlapping results indicate that *TCB1* might participate in the Pdr802 regulatory network, further suggesting the importance of *TCB1* to *C. neoformans* adaptation and survival in host-like conditions.

Finally, while the *TCB1* deletion caused changes in key in vitro virulence traits (capsule size, GXM shedding, and DMEM viability), the survival curve for the *tcb1*Δ mutant in the *T. molitor* insect model did not differ significantly from the wild-type control. This phenotype, combined with the lack of general stress susceptibility, is the defining feature arguing that *TCB1* has a highly specialized role in cryptococcal biology.

## 5. Conclusions

In conclusion, this study validates the utility of our integrative systems biology workflow for guiding the identification of specialized regulatory genes within complex fungal systems. *TCB1* is involved in the key processes related to the adaptation of *C. neoformans*, acting as a specific calcium-responsive gene associated with capsule size, GXM shedding, and viability regulation. The accumulation of molecular and functional evidence, especially the absence of susceptibility to core stressors and the non-attenuating virulence phenotype, strongly suggests that *TCB1* serves as a specialized node linking distinct signaling cascades, rather than participating as a canonical effector within the core calcineurin pathway. To our knowledge, this is the first study to investigate the role of tricalbins in *C. neoformans* biology, providing foundational insights for understanding the role of tricalbins as modulators of fungal adaptation.

## Data Availability

Raw *C. neoformans* RNA-seq sequence datasets used in this study were obtained from NCBI SRA database under accession code SRP120588.

## Supplementary Materials

The following supporting information can be downloaded at https://www.mdpi.com/article/10.3390/microorganisms14010122/s1. Supplementary File S1: Pmc1 PPIN Cytoscape session; Supplementary File S2: Cna1 PPIN Cytoscape session; Supplementary File S3: Crz1 PPIN Cytoscape session. Supplementary Table S1: Tcb1 (CNAG_00522) protein sequence; Supplementary Table S2: Transcription factor binding motifs for Crz1 and Pdr802; Supplementary Table S3: *TCB1* (*CNAG_00522*) promoter sequence; Supplementary Table S4: Primer sequences used in this study; Supplementary Table S5: Differentially expressed genes (DEGs); Supplementary Table S6: Cross-referencing Dynet PPIN overlaps with FungiDB annotations and conserved domain; Supplementary Table S7: Literature microarray and RNA-seq data evidence of possible *TCB1* participation in *C. neoformans* virulence and survival; Supplementary Table S8: FIMO results for TFBS in *TCB1* promoter region. Supplementary Figure S1: MultiQC STAR Alignment Scores plots; Supplementary Figure S2: Protein–protein interaction network of WT × *pmc1*Δ corresponding DEGs; Supplementary Figure S3: Protein–protein interaction network of WT × *cna1*Δ corresponding DEGs; Supplementary Figure S4: Protein–protein interaction network of WT × *crz1*Δ corresponding DEGs; Supplementary Figure S5: Construction of *tcb1*Δ mutant strain and confirmation; Supplementary Figure S6: Phenotypic stress susceptibility assays for *tcb1*Δ deletion mutant; Supplementary Figure S7: Virulence Assays of *tcb1*Δ and wild-type strains of *C. neoformans* in *T. molitor*.
