# Supplementary material for: Integrative Transcriptomic and Systems Biology Analyses Identify TCB1 as a Calcium-Responsive Gene in Cryptococcus neoformans
Source: Microorganisms. 2026 Jan 7;14(1):122. doi: 10.3390/microorganisms14010122 (PMC12843964; doi:10.3390/microorganisms14010122)
Supplement: Supplementary file 1 [file microorganisms-14-00122-s001.zip › Supplementary Figure S1.pdf]

SUPPLEMENTARY FIGURE S1

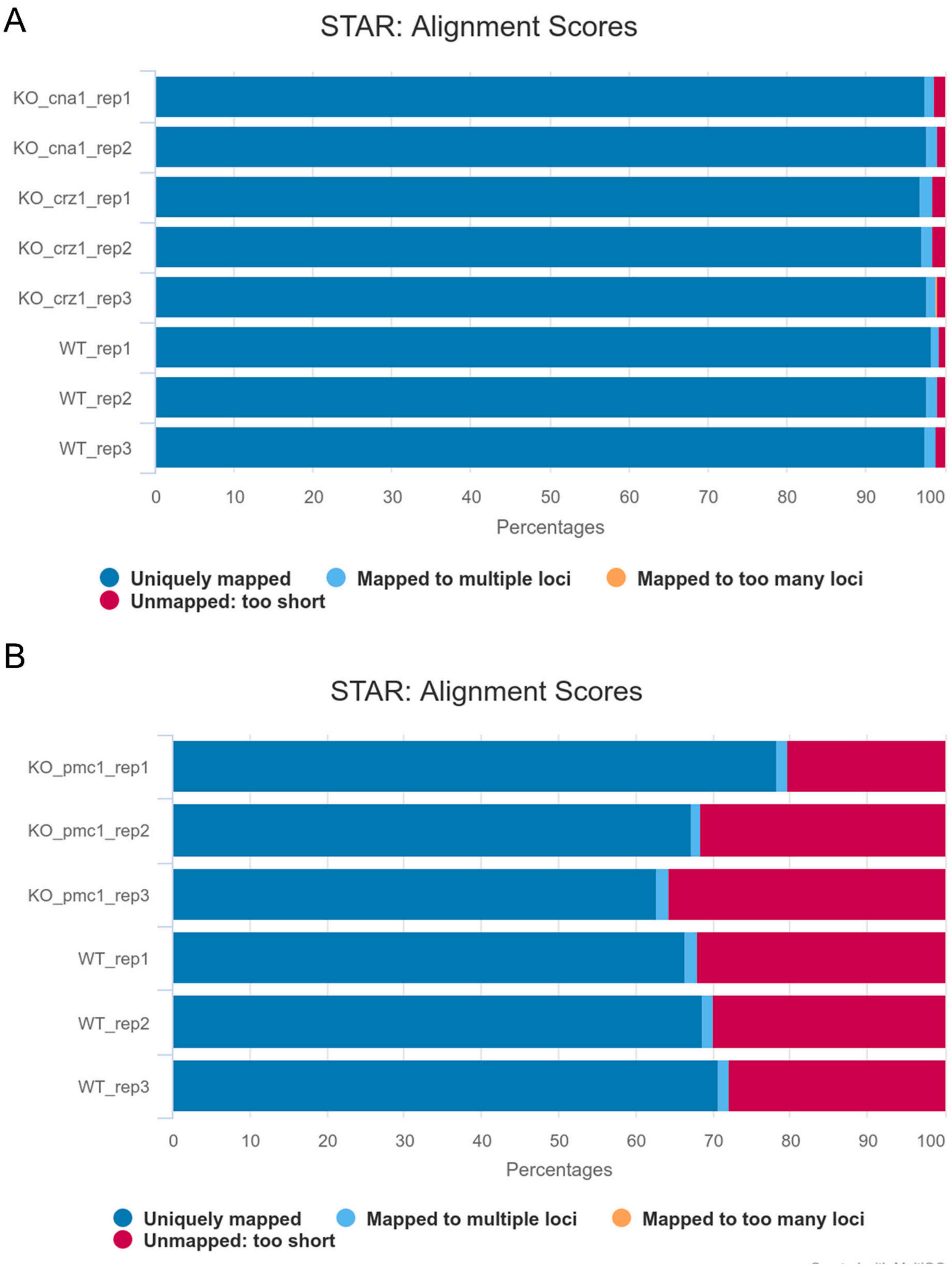

Supplementary Figure S1. MultiQC STAR Alignment Scores plots. STAR Alignment Scores plot for *C. neoformans cna1*Δ, *crz1*Δ and wild-type (WT) libraries (A), and *pmc1*Δ and wild-type (WT) libraries (B).
