# Supplementary material for: Integrative Transcriptomic and Systems Biology Analyses Identify TCB1 as a Calcium-Responsive Gene in Cryptococcus neoformans
Source: Microorganisms. 2026 Jan 7;14(1):122. doi: 10.3390/microorganisms14010122 (PMC12843964; doi:10.3390/microorganisms14010122)
Supplement: Supplementary file 1 [file microorganisms-14-00122-s001.zip › Supplementary Figure S4.pdf]

SUPPLEMENTARY FIGURE S4

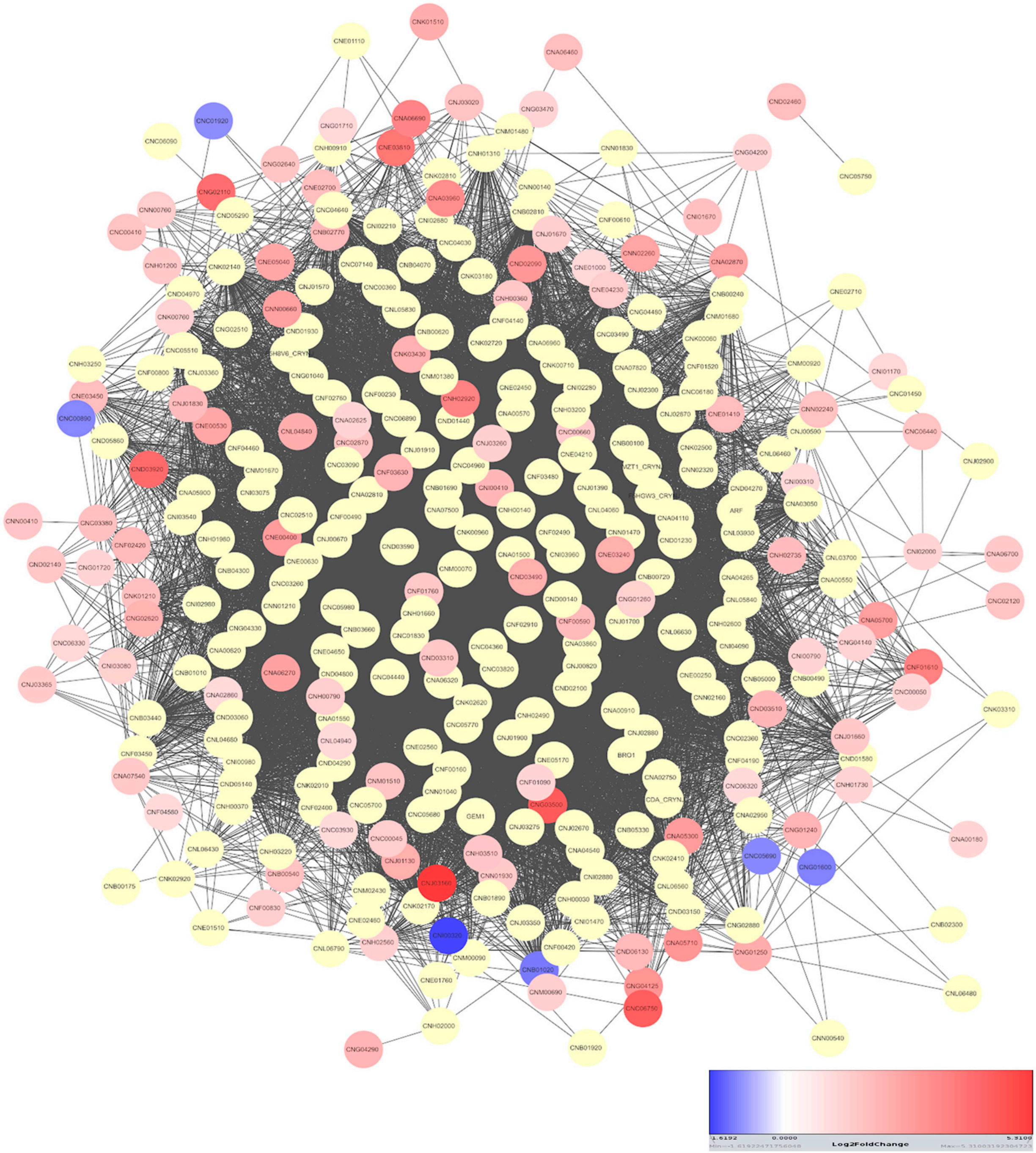

Supplementary Figure S4. Protein-protein interaction network of WT x *crz1*Δ corresponding DEGs. The interaction network is composed of 309 (109 DEGs) and 11,447 edges. Hues of red and blue indicate $Log_2FoldChange$ values for up and downregulated DEGs respectively. Light yellow nodes indicate first shell interactors.
