# Supplementary material for: Integrative Transcriptomic and Systems Biology Analyses Identify TCB1 as a Calcium-Responsive Gene in Cryptococcus neoformans
Source: Microorganisms. 2026 Jan 7;14(1):122. doi: 10.3390/microorganisms14010122 (PMC12843964; doi:10.3390/microorganisms14010122)
Supplement: Supplementary file 1 [file microorganisms-14-00122-s001.zip › Supplementary Figure S5.pdf]

SUPPLEMENTARY FIGURE S5

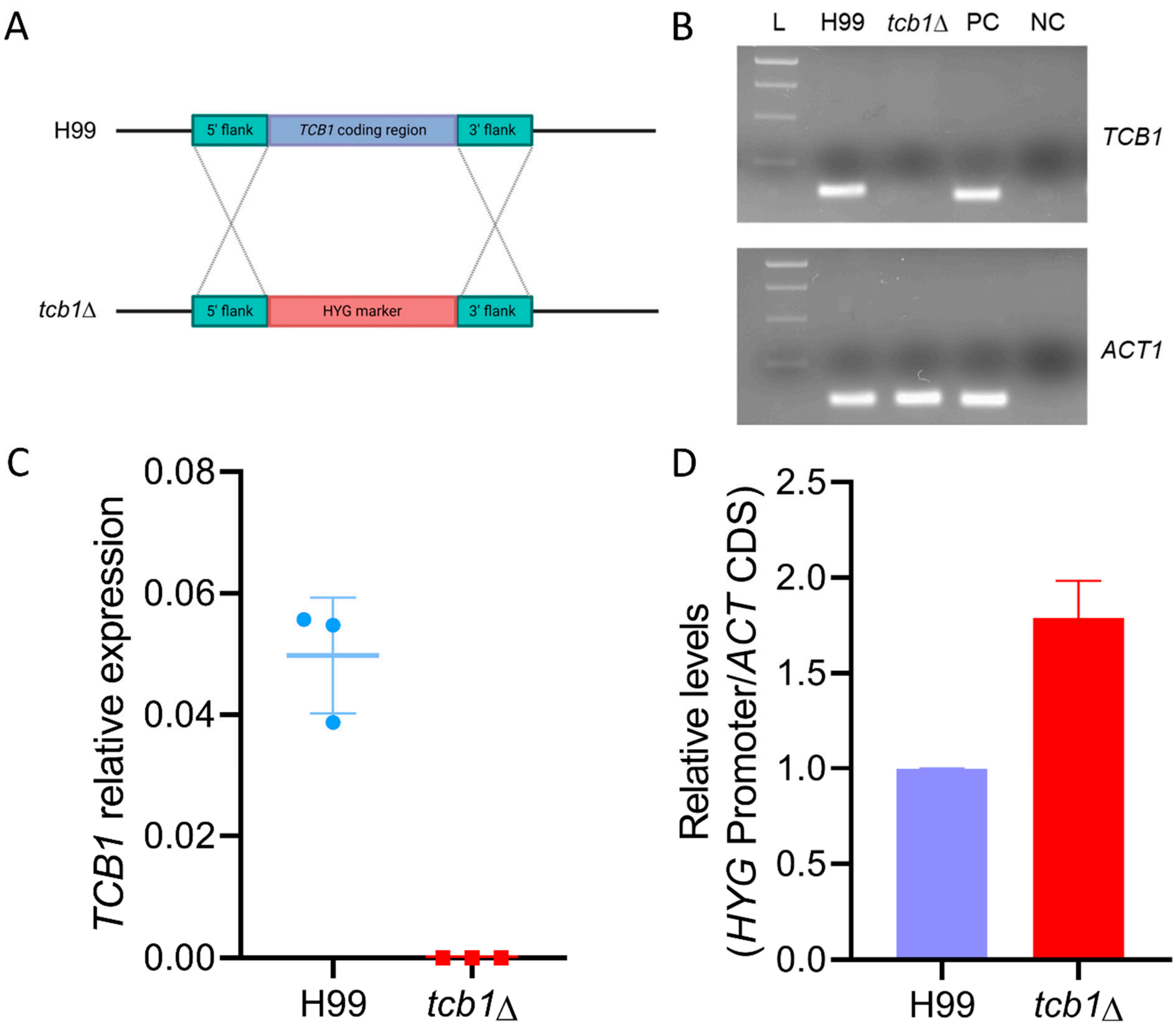

Supplementary Figure S5. Construction and confirmation of the *tcb1*Δ mutant strain. A. Schematic representation for deleting *TCB1* gene in the *C. neoformans* H99 background (image created with BioRender). B. Electrophoresis gel showing the results of the RT-PCR analysis of cDNA samples from the *C. neoformans* H99 wild-type and *tcb1*Δ strains. The upper gel shows the *TCB1* expression product, and the bottom gel shows *ACT1* expression. L, 1kb ladder (Promega); PC: RT-PCR positive control (H99 genomic DNA); NC: RT-PCR negative control. From top to bottom, the bands of the ladder represent 1000 bp, 750 bp, 500 bp, and 250 bp. C. Transcript levels of *TCB1* were assessed by qRT-PCR in the H99 wild-type and *tcb1*Δ strains after growth at 30°C, 200 rpm for 18 hours. Relative *TCB1* transcript levels were normalized by *ACT1* expression. Error bars indicate standard deviation. D. Validation of *TCB1* deletion by qPCR copy number analysis. To rule out ectopic integration of the deletion cassette, the relative copy number of the *ACT1* promoter (P_{ACT1}) was determined using genomic DNA from the H99 wild-type (WT) and *tcb1*Δ strains. Because *ACT1* is a single-copy gene and P_{ACT1} is also used to drive the hygromycin

resistance gene, a successful knockout mutant is expected to possess exactly two copies of the promoter sequence. This result confirms that the *tcb1*Δ mutant contains two copies of P_{ACT1}, indicating a single, targeted integration event at the *TCB1* locus.
