# Supplementary material for: Integrative Transcriptomic and Systems Biology Analyses Identify TCB1 as a Calcium-Responsive Gene in Cryptococcus neoformans
Source: Microorganisms. 2026 Jan 7;14(1):122. doi: 10.3390/microorganisms14010122 (PMC12843964; doi:10.3390/microorganisms14010122)
Supplement: Supplementary file 1 [file microorganisms-14-00122-s001.zip › Supplementary Figure S6.pdf]

SUPPLEMENTARY FIGURE S6

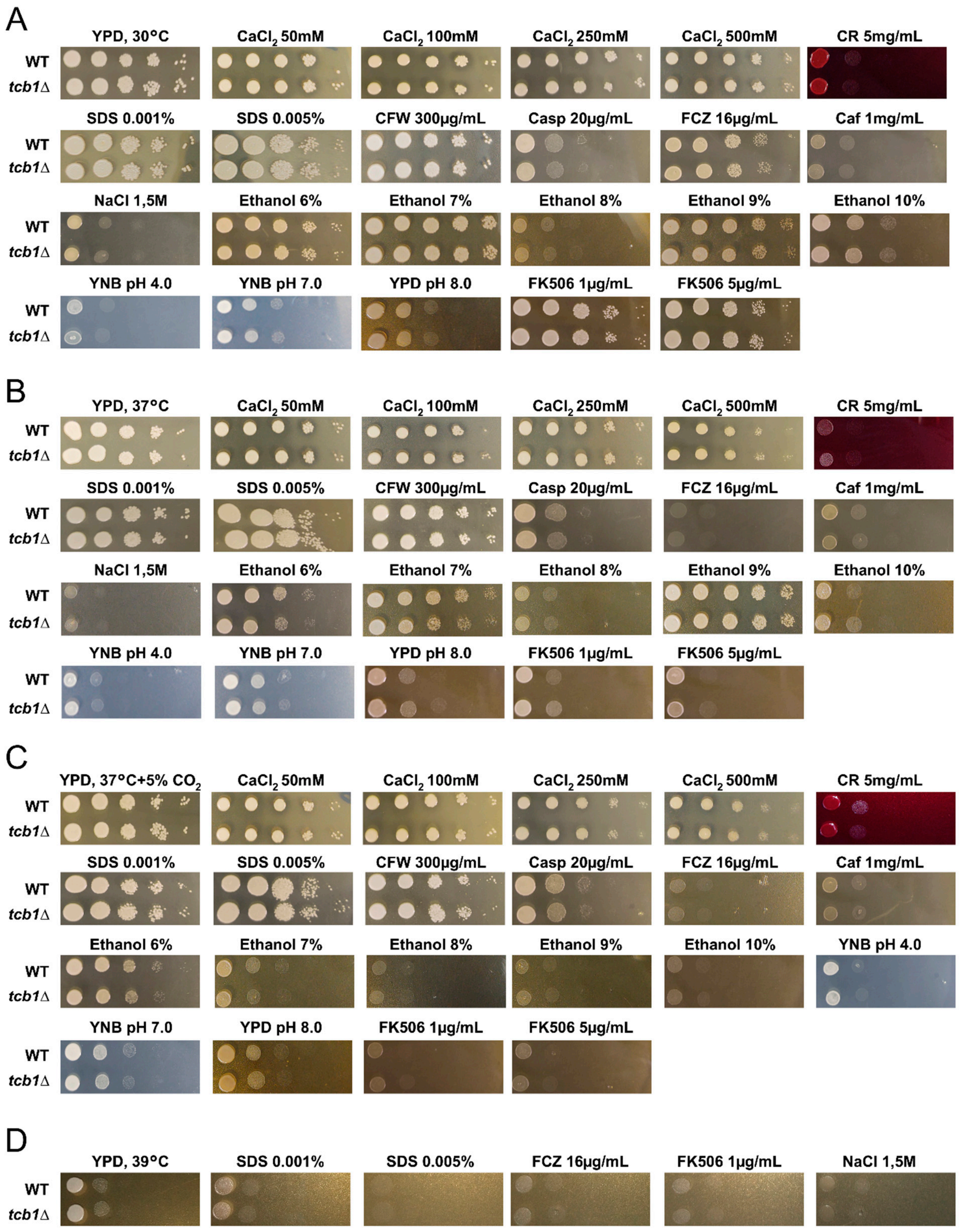

Supplementary Figure S6. Phenotypic stress susceptibility assays for *tcb1*Δ deletion mutant. Spot dilution assay for the wild-type and mutant strains. Ten-fold serial dilutions of fungal cells were inoculated onto specified media and incubated under various conditions: 30ºC (A), 37 ºC, 37ºC with 5% CO_2 (C), or 39ºC (D). The specified media used either YPD or YNB media bases to prepare varying pH conditions. Additional stress conditions included cell wall stressors, osmotic stressors, and antifungal agents (all assays not involving pH variation were performed on a YPD medium base). CR, Congo Red; CFW, calcofluor white; Casp, caspofungin; FCZ, fluconazole; Caf, caffeine.
