# Supplementary material for: Integrative Transcriptomic and Systems Biology Analyses Identify TCB1 as a Calcium-Responsive Gene in Cryptococcus neoformans
Source: Microorganisms. 2026 Jan 7;14(1):122. doi: 10.3390/microorganisms14010122 (PMC12843964; doi:10.3390/microorganisms14010122)
Supplement: Supplementary file 1 [file microorganisms-14-00122-s001.zip › Supplementary Figure S7.pdf]

SUPPLEMENTARY FIGURE S7

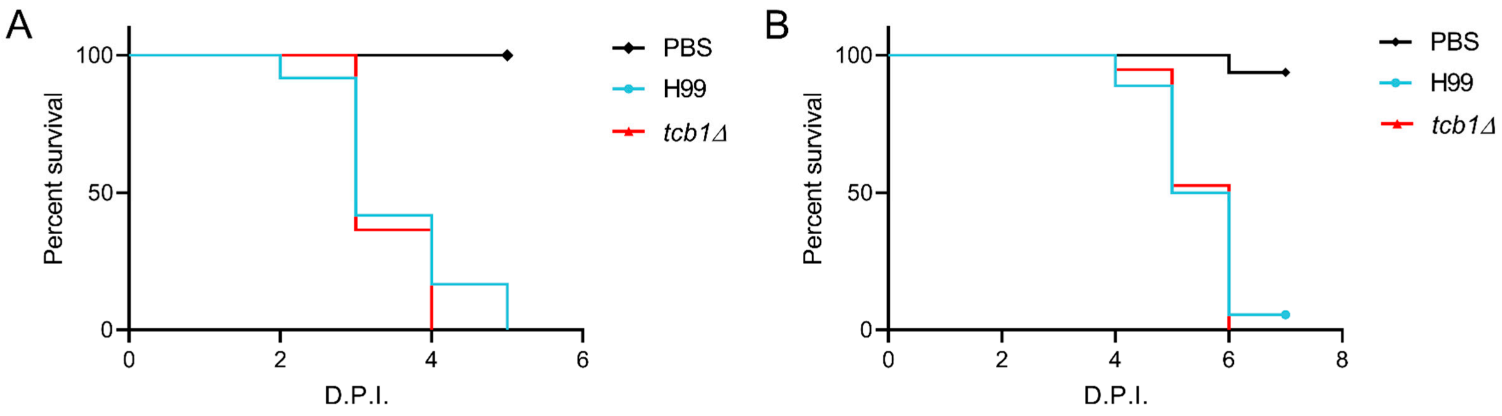

Supplementary Figure S7. Virulence Assays of *tcb1*Δ and wild-type strains of *C. neoformans* in *T. molitor*. Survival curve of *T. molitor* larvae injected with 5 µL of PBS (negative control) and with a (A) high inoculum of 1×10^5 cells (corresponding to 2×10^4 cells/µL) and (B) a lower inoculum of 1×10^4 cells (corresponding to 2×10^3 cells/µL) of the WT and *tcb1*Δ *C. neoformans* strains.
