# Supplementary material for: Integrative Transcriptomic and Systems Biology Analyses Identify TCB1 as a Calcium-Responsive Gene in Cryptococcus neoformans
Source: Microorganisms. 2026 Jan 7;14(1):122. doi: 10.3390/microorganisms14010122 (PMC12843964; doi:10.3390/microorganisms14010122)
Supplement: Supplementary file 1 [file microorganisms-14-00122-s001.zip › Supplementary Table S1.pdf]

Supplementary Table S1. Tcb1 (CNAG_00522) protein sequence.

CNAG_00522 protein sequence | *Cryptococcus neoformans* var. *grubii* H99
MVERRAREAEERERRARESRSGIGGDGHAAPQSGSPDAHGARAPVKAGPDEETNAAK<br>VQEGKKTDEPNAASGANEKARMMEQMNANQLKPTERFAKAEKGQRRVRDPITGAEII<br>VKDADPKDFDSKQPATKGTNVLYHAFPPPQPISVRIALDKLKLIQYGIAGFSFFIWLTVS<br>FGSGLLAFVYRSILCSVSAFILMTAVSLVERSLEKDVEKVRQDMGRQRGEAFSPPVPES<br>VEWLNGLIKLVWGLLDPAMFVSIADMVEDILQQSLPGFVDAVRITDIGQGTNPIRITSIR<br>ALPDQPGDEGYPHTGWINQGNDDIKTKDTSGKDVEEDEAGDYYNFEVAFSYAALPGQ<br>GAHQRARNIHLLIEFFLGLYDWLHIPIPIWIQVEQIFGIIRLRVQFIPQPPFVRNLTFALCGV<br>PAVEVSAIPMSRHLPNVLDLPFVSSFVKMGIAAGTAEMSVPKSMTINLQEMLSGAAVG<br>DTRAIGVFLITVHHCTGLSSQDNNGLSDPYVVLAYAKFGKPLYSTRIILEDLNPVYEETC<br>VLLLTMDEVKSKEDLAAMLWDSDNLSADDLVGRVQIPVEELMLKPNQMIRREDSLMG<br>FEDANDMPGKLVWSIGYFEKAPLRKELEQGPTVEEAANTTDAAPKTAPEMEMHPADIA<br>PNPAAKDLPPPPPDVLKTRPDPRWPSGVLSIILHQVNNLERQNLKGASGNNREGEAGQD<br>TDQPSEQSDNLPSGYGEFIVNDDLVYKTRVKQYSTNPYFEAGTEVFVRDFENTVVRVV<br>VRDSRLREADPILGIVSVRLSEVFREASSVNQTYALTEGVGFGKANISFAFRGMQMTLP<br>PNMRGWETGTLEVSDVRFTSTNPSLFDAQATRLRVVTSEQAEVLPKKEAEVQGHTISW<br>EMDMLRLPVYARYQSSVVFEIGKTGGPLSIVGVGVKPDAIAVLWMQDLTDDIEQEVKL<br>PVLVSSKIENLRQNAINDQTQKHHDFKVVGELVARIKLDSGLDEDHEQNLKQSQSRRH<br>ALEAYDHIEGEAEVARKQTHFADDGVIDKHEKKEIDKAHKRQLQSRERGPAQVKAYRS<br>AKWMTKGIKDRLPGNKPKTREPTVQTEV
