# Supplementary material for: Integrative Transcriptomic and Systems Biology Analyses Identify TCB1 as a Calcium-Responsive Gene in Cryptococcus neoformans
Source: Microorganisms. 2026 Jan 7;14(1):122. doi: 10.3390/microorganisms14010122 (PMC12843964; doi:10.3390/microorganisms14010122)
Supplement: Supplementary file 1 [file microorganisms-14-00122-s001.zip › Supplementary Table S2.pdf]

Supplementary Table S2. Transcription factor binding motifs for Crz1 and Pdr802.

Motif_ID	Motif Sequence
Crz1_1	5’-[A/G]CACAGC[A/C]AC-3’
Crz1_2	5’-GAAGATG[A/G]T[A/G]-3’
Crz1_3	5’-GCACA[A/G]C-3’
Pdr802_1	5’-C[A/C/T]TCTTC[C/T]-3’
Pdr802_2	5’-GA[C/G/T]GA[C/T]GA-3’
Pdr802_3	5’-GA[A/C]GA-GA-3’
Pdr802_4	5’-C[C/T]TCC[A/G/T]CC-3’
