# Supplementary material for: Integrative Transcriptomic and Systems Biology Analyses Identify TCB1 as a Calcium-Responsive Gene in Cryptococcus neoformans
Source: Microorganisms. 2026 Jan 7;14(1):122. doi: 10.3390/microorganisms14010122 (PMC12843964; doi:10.3390/microorganisms14010122)
Supplement: Supplementary file 1 [file microorganisms-14-00122-s001.zip › Supplementary Table S3.pdf]

Supplementary Table S3. ***TCB1*** **(*****CNAG_00522*****) promoter sequence.**

CNAG_00522 | *Cryptococcus neoformans* var. *grubii* H99
CGCCGGTGGAAGGGGAAATGAAGATAGGGAGAGACGAAAAGGAGGAGAGGGCGA<br>GAGAGGAGAGACCGATGAGCGAGAGTGGGAGCGAGAGGGATAGTGTAGGGTGGG<br>TCGGGAAGGAGAGGGATGCTTTTAGGAGAGGTGTGATTCAGCTTTATATGCGGCGG<br>AAAACTGTACCGGGACGTACCGCACGTGAGACAGGCAAAGTAGGAGCCGACGGTG<br>GCTGCGCCGACCAGGAAATATGCGGCCGTAAGCCATATATATAAAAGGGGTGTCTC<br>CGGGGTGGATTTGGCAAGTATGGCGGAGAATGACAGGTAGCCAGCTGCGCTCAGA<br>CAGGCCGAGACGAGGGAGCCTCTGTCCAGCGTAGCAGACGTCAGGTACAGAGTAT<br>GGTGGCACACACAGCCAAACACCTACATCCTTGGGCCGTATTTATCCGTAAGAGAT<br>CCCAGCGGCGCAGCCATGAGGTATACGCCTACGATCCCGCTTGGCACCGTCTGGGT<br>CAGCGGAGGGAGCTACACAAGAAGAAGACGCAGACGCACCCGACGACGATTGTCT<br>GTGCCTGTGTGCCGTCGAGCTGGCGCATGCTGGCGACGACGGGGCCGTAGGTGCCC<br>CAGCAGTAGACGCCGTTGGCCTGGAGGGCGCTGGCGGCGATGGAGAGGCATGTGA<br>GGGCGGCGGCGAGCGGGCGGGGGAGGCGAAGGCGGGGGAGGCGGAGTGTGCCGC<br>GCCGCTTTGCGTGGAGGATGCGGGGTATGTCGGGGGGGGAGGTGTCAGCGCTGCGC<br>TCGATGTCGTACAGCACCACGGGGGCGCCCTCGCTCGACATGGCCGGTGGACAACA<br>CACACAGACATCCGCAACCAGCGCCCGGAAGAGGGGCGCCAGGCGGCGGGCGCCG<br>ACTCGCAGAGAGCACTTCCCCGCATACGTCATAGCCCTCCCCCGCGCGCCAGCCGT<br>CTTAATATATCGCAGACGCAGCGGGAACTTTGCCCACCCACAACACCTCCCCGATG<br>CCCC
