# Supplementary material for: Integrative Transcriptomic and Systems Biology Analyses Identify TCB1 as a Calcium-Responsive Gene in Cryptococcus neoformans
Source: Microorganisms. 2026 Jan 7;14(1):122. doi: 10.3390/microorganisms14010122 (PMC12843964; doi:10.3390/microorganisms14010122)
Supplement: Supplementary file 1 [file microorganisms-14-00122-s001.zip › Supplementary Table S4.pdf]

Supplementary Table S4. Primer sequences used in this study.

Primer name	Sequence (5' - 3')
*TCB1*_qPCR_F	GTCTCCTTGCTTTCGTTTAC
*TCB1*_qPCR_R	ATCCTGCCTTACCTTTTCC
*ACT1*_CDS_qPCR_F	CAAGCAGAACCGAGAGAAG
*ACT1*_CDS_qPCR_R	CGTCACCAGAGTCAAGAAC
*ACT1*_promoter_qPCR_F	GAGGAGAGGATGATGGTAAC
*ACT1*_promoter_qPCR_R	GGTATAGGGGATGTATGTGG
*TCB1*_deletion_5'UTR_F	AGACAGGCAAAGTAGGAGCC
*TCB1*_deletion_5'UTR_R	CACTGGCCGTCGTTTTACCTCTCGACCATCTCTGCGTA
*TCB1*_deletion_3'UTR_F	CATGGTCATAGCTGTTTCCTGAAATGTTTCATGTCCCTTGC
*TCB1*_deletion_3'UTR_R	GACGAGAGCTAGAAAGGCTG
