# Supplementary material for: Integrative Transcriptomic and Systems Biology Analyses Identify TCB1 as a Calcium-Responsive Gene in Cryptococcus neoformans
Source: Microorganisms. 2026 Jan 7;14(1):122. doi: 10.3390/microorganisms14010122 (PMC12843964; doi:10.3390/microorganisms14010122)
Supplement: Supplementary file 1 [file microorganisms-14-00122-s001.zip › Supplementary Table S5.pdf]

Supplementary Table S5. Differentially expressed genes (DEGs).

DEGs for WT vs *crz1Δ*			
**Gene ID**	**Log_2FoldChange**	**p-value**	**p-adj**
CNAG_00588	5.468347887	1.83E-97	1.43E-93
CNAG_04891	5.310031923	5.70E-30	8.91E-27
CNAG_12967	4.979300875	3.01E-14	8.11E-12
CNAG_03223	4.48616927	5.34E-54	2.09E-50
CNAG_01506	4.394424026	4.46E-13	9.69E-11
CNAG_02864	4.333806042	3.42E-15	1.03E-12
CNAG_12762	4.190751807	4.69E-12	8.34E-10
CNAG_07498	4.07838054	6.20E-19	3.03E-16
CNAG_01272	4.06149615	7.71E-10	1.08E-07
CNAG_07725	3.949589175	2.86E-05	0.00186518999
CNAG_02156	3.751499775	8.13E-15	2.35E-12
CNAG_12655	3.66174486	7.12E-24	7.94E-21
CNAG_05818	3.470342773	9.83E-13	2.02E-10
CNAG_05654	3.409572319	4.51E-06	0.0003632746062
CNAG_00691	3.362092255	1.54E-20	1.09E-17
CNAG_05412	3.35046008	3.50E-13	7.81E-11
CNAG_00407	3.118718214	6.60E-22	5.73E-19
CNAG_03154	2.922600761	2.59E-40	6.74E-37
CNAG_00587	2.895208608	3.05E-19	1.70E-16
CNAG_01081	2.852134969	3.20E-23	3.13E-20
CNAG_12183	2.749792213	4.48E-05	0.002755852442

CNAG_00546	2.73573402	3.90E-18	1.79E-15
CNAG_02526	2.71395694	1.75E-17	7.58E-15
CNAG_05916	2.695837278	1.23E-12	2.46E-10
CNAG_03650	2.662988843	4.12E-14	1.07E-11
CNAG_00586	2.641876569	4.52E-05	0.002761562263
CNAG_06336	2.618690744	3.85E-10	5.48E-08
CNAG_05158	2.611435101	6.82E-21	5.33E-18
CNAG_12252	2.538239381	0.0009131993082	0.03604369997
CNAG_00647	2.536868588	3.23E-16	1.26E-13
CNAG_00301	2.507970121	4.17E-12	7.94E-10
CNAG_06501	2.490326428	1.31E-11	2.18E-09
CNAG_05641	2.488220085	1.01E-11	1.71E-09
CNAG_02510	2.448573227	3.46E-28	4.51E-25
CNAG_02030	2.405538163	7.91E-11	1.24E-08
CNAG_05032	2.39643328	1.80E-07	1.76E-05
CNAG_12370	2.357184843	9.35E-07	7.94E-05
CNAG_03224	2.313193089	2.97E-07	2.73E-05
CNAG_03783	2.304614767	2.00E-14	5.60E-12
CNAG_02661	2.263718915	9.67E-12	1.68E-09
CNAG_03465	2.258499781	1.82E-11	2.97E-09
CNAG_12756	2.25318384	7.20E-06	0.0005461230866
CNAG_05138	2.250783151	3.64E-05	0.002331243293
CNAG_01230	2.226505554	4.02E-20	2.62E-17
CNAG_02217	2.214845589	0.0002508421277	0.0119532392

CNAG_04681	2.213252901	1.50E-06	0.0001260830719
CNAG_02008	2.198273971	4.94E-06	0.0003940312454
CNAG_04280	2.1823887	8.19E-07	7.11E-05
CNAG_03316	2.078399138	7.03E-14	1.77E-11
CNAG_03464	2.065872286	2.91E-10	4.30E-08
CNAG_06658	2.047870555	7.72E-14	1.89E-11
CNAG_03135	2.038267348	2.60E-15	8.46E-13
CNAG_12591	2.026483506	0.0009585425831	0.03745505144
CNAG_02685	2.015607292	3.10E-30	6.05E-27
CNAG_01232	1.976202636	2.73E-19	1.64E-16
CNAG_04514	1.967387127	6.46E-09	7.89E-07
CNAG_06835	1.905812751	0.0001045827925	0.005636651883
CNAG_05915	1.89108529	2.04E-16	8.40E-14
CNAG_12834	1.887442118	0.0003541518917	0.01590630479
CNAG_05731	1.880476089	4.53E-12	8.24E-10
CNAG_03782	1.878357025	1.21E-07	1.24E-05
CNAG_05632	1.847633612	7.49E-06	0.0005597839857
CNAG_04752	1.840844431	3.97E-07	3.60E-05
CNAG_05159	1.813138982	1.03E-13	2.44E-11
CNAG_02415	1.809233081	5.94E-09	7.37E-07
CNAG_13146	1.804461972	3.43E-15	1.03E-12
CNAG_01121	1.795384601	3.07E-11	4.90E-09
CNAG_02194	1.791844297	9.00E-05	0.00509915667
CNAG_07638	1.771298664	1.41E-05	0.0009731322051

CNAG_00668	1.754769349	2.88E-13	6.61E-11
CNAG_12604	1.728762341	0.0002032480782	0.01018194699
CNAG_12383	1.725705011	0.0007279423311	0.02994141746
CNAG_06863	1.709897827	0.001236882333	0.04640980209
CNAG_04606	1.704203758	4.52E-12	8.24E-10
CNAG_07549	1.691249401	3.37E-05	0.002174398687
CNAG_04380	1.689971353	9.30E-08	9.96E-06
CNAG_01854	1.688234961	5.40E-16	2.01E-13
CNAG_06748	1.677320623	7.45E-09	8.95E-07
CNAG_06499	1.665486847	0.0005905807443	0.02522070228
CNAG_04874	1.628689707	4.63E-05	0.002784654499
CNAG_07658	1.627351014	0.0005128634975	0.02202213315
CNAG_03047	1.62280461	0.0004145195379	0.01809759882
CNAG_06507	1.620527701	1.28E-07	1.30E-05
CNAG_01539	1.612367827	2.05E-06	0.0001706558131
CNAG_01796	1.610414266	6.76E-06	0.000518017202
CNAG_01704	1.607748034	3.96E-05	0.002517538096
CNAG_06149	1.588352483	5.39E-19	2.81E-16
CNAG_03314	1.579799255	8.45E-16	3.00E-13
CNAG_07723	1.571839055	1.23E-05	0.0008650058232
CNAG_05277	1.552951061	8.40E-09	9.95E-07
CNAG_06311	1.545600996	1.01E-09	1.36E-07
CNAG_07406	1.537632443	2.89E-09	3.77E-07
CNAG_07873	1.5326748	7.49E-07	6.58E-05

CNAG_01207	1.526999065	8.18E-10	1.12E-07
CNAG_01087	1.526473517	1.99E-07	1.90E-05
CNAG_05803	1.50887272	2.53E-08	2.82E-06
CNAG_00776	1.47279606	3.53E-12	6.89E-10
CNAG_05458	1.467389265	3.45E-09	4.43E-07
CNAG_04736	1.460151153	0.0001304954576	0.006890689197
CNAG_06473	1.455872223	1.20E-15	4.09E-13
CNAG_00692	1.451732218	4.22E-06	0.0003431906409
CNAG_01131	1.429446067	5.64E-06	0.0004406899478
CNAG_03019	1.412129022	0.0002371259293	0.01151018098
CNAG_04916	1.406741339	0.0004288177916	0.01861783912
CNAG_02114	1.390181555	8.20E-13	1.73E-10
CNAG_06347	1.38738532	0.00033253883	0.01502191304
CNAG_13023	1.38296272	8.49E-06	0.0006142909316
CNAG_06973	1.367356837	2.50E-09	3.31E-07
CNAG_07407	1.365681298	0.001174976795	0.04435963116
CNAG_12867	1.355677907	0.0003105340873	0.01427543466
CNAG_05411	1.342938099	1.06E-07	1.12E-05
CNAG_04737	1.313735544	0.0001708676725	0.008670979615
CNAG_06060	1.313392141	1.90E-07	1.84E-05
CNAG_04903	1.30153098	4.94E-05	0.002940851118
CNAG_05889	1.298140775	4.17E-05	0.002604951425
CNAG_03161	1.284629628	0.0002294587353	0.01127811331
CNAG_05615	1.265845797	1.63E-07	1.61E-05

CNAG_06016	1.264845257	2.07E-08	2.35E-06
CNAG_04171	1.25737864	0.000209029405	0.01040487134
CNAG_03463	1.244690338	9.06E-09	1.06E-06
CNAG_02585	1.234679853	8.89E-07	7.63E-05
CNAG_03152	1.2321257	1.07E-05	0.0007696089184
CNAG_02169	1.220493205	0.0003203423703	0.01464020833
CNAG_05521	1.18983166	1.32E-05	0.0009217557149
CNAG_03227	1.185178821	0.0002884386642	0.01341754858
CNAG_04350	1.179573959	9.19E-11	1.41E-08
CNAG_12695	1.174251923	0.0002108332252	0.01042823832
CNAG_04478	1.172993461	1.52E-05	0.001034577213
CNAG_02602	1.165348453	0.0002460638052	0.01179747631
CNAG_03412	1.162382492	1.11E-10	1.66E-08
CNAG_03146	1.155059135	0.0002905658475	0.01343652129
CNAG_05864	1.147516885	8.15E-06	0.0005954012561
CNAG_00274	1.134968872	0.0006188698874	0.02600251704
CNAG_00025	1.134834745	5.66E-08	6.23E-06
CNAG_07499	1.132633326	4.02E-05	0.002533618658
CNAG_05326	1.088355342	9.72E-05	0.005348497056
CNAG_01551	1.085734641	0.0009686116363	0.03759197443
CNAG_04440	1.084253434	0.0002437899848	0.01176060945
CNAG_02458	1.06642289	6.32E-06	0.0004892703558
CNAG_03413	1.061442934	3.58E-06	0.0002948363176
CNAG_04524	1.041768782	4.01E-07	3.60E-05

CNAG_07812	1.020112379	6.83E-05	0.003956637798
CNAG_02774	1.006898855	9.81E-05	0.005361730814
CNAG_01552	1.003323449	0.0001429960353	0.007450093438
CNAG_00300	1.000713077	0.000979303125	0.03770075824
CNAG_06559	1.000404863	0.0001120878355	0.005999770099
CNAG_01681	-1.007218293	5.60E-06	0.0004406899478
CNAG_02588	-1.022665644	0.0001577619285	0.008111246523
CNAG_02958	-1.038361733	2.59E-05	0.001702962921
CNAG_07845	-1.040291415	1.22E-05	0.0008650058232
CNAG_01574	-1.070075556	3.86E-10	5.48E-08
CNAG_01070	-1.102127946	9.68E-05	0.005348497056
CNAG_03426	-1.105334152	2.22E-05	0.001469183315
CNAG_03599	-1.174885517	0.0003241657393	0.01472880961
CNAG_04523	-1.619224718	0.0002616756179	0.01225476333
**DEGs for WT vs *cna1*Δ**			
**Gene ID**	**$Log_2FoldChange$**	**p-value**	**p-adj**
CNAG_12252	7.269232648	1.26E-05	0.0005690289717
CNAG_04891	7.015926394	1.57E-62	6.00E-59
CNAG_04291	6.820726388	1.04E-12	2.95E-10
CNAG_07870	6.743680303	4.38E-05	0.001570541998
CNAG_04459	6.588220313	1.21E-11	2.72E-09
CNAG_00588	6.422745431	5.03E-109	3.84E-105
CNAG_02864	5.713670864	3.08E-22	2.61E-19
CNAG_03759	5.523953148	4.50E-39	6.86E-36

CNAG_03007	5.523287985	1.15E-17	5.85E-15
CNAG_05268	5.487409781	0.003013307821	0.0422749362
CNAG_00269	5.192512412	1.23E-07	9.65E-06
CNAG_02591	5.117934366	5.41E-11	9.83E-09
CNAG_12133	5.072599802	0.002068704706	0.03213567102
CNAG_00091	5.052836516	3.46E-11	7.10E-09
CNAG_07981	4.94625539	1.44E-12	3.85E-10
CNAG_07745	4.689829762	2.29E-08	2.18E-06
CNAG_05939	4.681628732	8.20E-12	1.90E-09
CNAG_06207	4.579202675	4.15E-08	3.73E-06
CNAG_12450	4.548663369	0.0004383264082	0.01032502206
CNAG_12967	4.528757077	4.81E-12	1.18E-09
CNAG_05654	4.411364129	2.32E-11	5.05E-09
CNAG_12183	4.383741476	1.93E-13	6.69E-11
CNAG_00407	4.331528877	5.23E-28	6.65E-25
CNAG_13060	4.234452415	3.21E-08	2.95E-06
CNAG_07939	4.193607243	2.03E-08	1.96E-06
CNAG_02526	4.192769908	1.73E-22	1.65E-19
CNAG_13201	4.147163961	0.002741966875	0.03933588569
CNAG_12812	4.094867725	1.29E-05	0.0005767407931
CNAG_12392	4.046072819	7.59E-06	0.0003731608612
CNAG_05641	4.03911961	4.07E-20	2.82E-17
CNAG_02751	4.004882613	1.18E-06	7.49E-05
CNAG_00586	3.984265442	8.02E-10	1.07E-07

CNAG_13083	3.922404734	1.24E-07	9.67E-06
CNAG_03224	3.883047589	5.59E-07	3.95E-05
CNAG_04903	3.872260527	6.23E-21	4.75E-18
CNAG_03223	3.808885431	1.28E-39	2.50E-36
CNAG_01081	3.772898935	9.41E-27	1.03E-23
CNAG_04105	3.739825166	5.15E-06	0.0002618463965
CNAG_04322	3.688148013	1.25E-06	7.86E-05
CNAG_04585	3.630704764	9.25E-07	6.25E-05
CNAG_12995	3.622499724	9.19E-05	0.002923968286
CNAG_05448	3.604037118	7.00E-08	6.14E-06
CNAG_02156	3.603808428	1.75E-15	7.86E-13
CNAG_07725	3.602596944	0.0001631889812	0.004578890824
CNAG_03143	3.552270387	2.52E-08	2.37E-06
CNAG_06853	3.545547296	2.58E-05	0.001041405081
CNAG_12182	3.504082894	0.0003387000616	0.008475274985
CNAG_01942	3.487259433	8.21E-08	6.96E-06
CNAG_06835	3.48312922	8.95E-14	3.25E-11
CNAG_01090	3.466418622	3.58E-06	0.0001938543309
CNAG_01803	3.465643621	1.67E-07	1.27E-05
CNAG_02070	3.417742388	6.90E-09	7.86E-07
CNAG_00485	3.39163159	8.38E-08	7.01E-06
CNAG_05916	3.382654056	1.34E-08	1.34E-06
CNAG_12773	3.374364628	0.0001040940889	0.003189220622
CNAG_01506	3.371687111	2.34E-09	3.03E-07

CNAG_06297	3.366055452	2.37E-07	1.79E-05
CNAG_06286	3.356664604	6.87E-05	0.002307474748
CNAG_02253	3.288965633	0.003116488376	0.04324552597
CNAG_12655	3.264745855	2.42E-19	1.42E-16
CNAG_02685	3.263892131	1.31E-39	2.50E-36
CNAG_13059	3.263277906	0.0006987071202	0.01473075343
CNAG_12315	3.25656284	0.001092934786	0.02044430953
CNAG_06388	3.24650981	7.53E-06	0.0003729292897
CNAG_13107	3.22425707	0.0005565386493	0.0124926558
CNAG_00979	3.220079793	1.12E-07	8.91E-06
CNAG_00519	3.210132067	1.39E-05	0.0006158757994
CNAG_00587	3.153642286	5.94E-20	3.78E-17
CNAG_12020	3.150198078	0.0008840351514	0.01789643574
CNAG_13125	3.11427215	0.000615902808	0.01358546309
CNAG_12606	3.075251511	1.38E-06	8.61E-05
CNAG_04043	3.039929426	1.75E-05	0.0007634113476
CNAG_06238	3.015878398	8.50E-09	9.40E-07
CNAG_03679	2.993190837	3.29E-06	0.000181938655
CNAG_12900	2.992414671	0.001984986989	0.0316271831
CNAG_12491	2.966798007	0.0001627753607	0.004578890824
CNAG_05632	2.94780045	1.02E-12	2.95E-10
CNAG_04926	2.906386305	0.000183444021	0.005018081606
CNAG_13023	2.904700751	7.23E-12	1.72E-09
CNAG_01341	2.902109313	3.66E-05	0.001364119618

CNAG_12913	2.888008623	2.45E-05	0.0009940564582
CNAG_02899	2.872289897	7.46E-06	0.0003720934584
CNAG_06805	2.868518466	5.83E-06	0.0002928047779
CNAG_12663	2.852784843	0.002356222884	0.03539900207
CNAG_01588	2.843708204	1.66E-05	0.0007301214213
CNAG_05449	2.843388987	0.0001128739104	0.003391549936
CNAG_03492	2.830035038	6.93E-11	1.17E-08
CNAG_00848	2.82314627	3.89E-08	3.53E-06
CNAG_12860	2.813751037	0.001595692999	0.02706295326
CNAG_06396	2.804504316	1.13E-08	1.17E-06
CNAG_12770	2.803719236	0.0001460883035	0.004197425668
CNAG_01735	2.801294193	3.95E-05	0.001449562174
CNAG_06863	2.791736557	1.99E-09	2.61E-07
CNAG_01102	2.781380703	9.64E-05	0.003015919143
CNAG_07498	2.777931848	5.52E-09	6.58E-07
CNAG_12993	2.769722199	0.0001904609724	0.005136389192
CNAG_06109	2.756553177	1.40E-07	1.08E-05
CNAG_02182	2.754869337	0.0003124101557	0.007947714362
CNAG_03650	2.752653764	4.95E-13	1.57E-10
CNAG_12713	2.748415675	2.52E-09	3.16E-07
CNAG_06298	2.747261525	1.15E-05	0.0005315940641
CNAG_03084	2.740038611	6.89E-05	0.002307474748
CNAG_12762	2.737515118	2.14E-06	0.0001230541438
CNAG_06169	2.700900074	9.74E-08	7.91E-06

CNAG_03058	2.696124055	8.11E-06	0.0003896793402
CNAG_06291	2.695827478	3.92E-07	2.85E-05
CNAG_04025	2.689583189	2.67E-05	0.00107279513
CNAG_01464	2.648757308	1.39E-06	8.65E-05
CNAG_12113	2.642172089	0.0001208577707	0.003561337861
CNAG_03783	2.62941634	9.36E-13	2.86E-10
CNAG_00691	2.620911912	4.48E-10	6.57E-08
CNAG_01736	2.612195687	0.00239865648	0.03582494375
CNAG_12974	2.610951376	3.78E-05	0.001400138896
CNAG_03782	2.608269164	6.61E-09	7.65E-07
CNAG_12558	2.606103249	0.002019249997	0.03173433226
CNAG_01230	2.603454149	9.76E-19	5.32E-16
CNAG_12217	2.593067037	5.02E-05	0.001767319021
CNAG_03465	2.5859822	3.84E-11	7.40E-09
CNAG_05387	2.561339768	2.72E-07	2.02E-05
CNAG_03958	2.558291592	0.000544839378	0.01226611839
CNAG_12864	2.545415687	1.97E-06	0.0001155985494
CNAG_02986	2.498136035	0.0006715221701	0.0143282197
CNAG_02577	2.497612842	1.76E-05	0.0007634113476
CNAG_13055	2.495195316	0.003156386224	0.0437196727
CNAG_04027	2.493651861	3.39E-06	0.0001862948676
CNAG_05994	2.472932411	1.12E-05	0.0005232794117
CNAG_00011	2.469667251	0.0001759637677	0.004853929941
CNAG_06668	2.467771248	0.000497291952	0.01136326999

CNAG_01345	2.461422255	0.00268680688	0.03881776143
CNAG_04206	2.437851393	3.65E-05	0.001364119618
CNAG_06574	2.427194329	0.003263565146	0.0447172876
CNAG_01446	2.414813171	0.0001190848086	0.003536401786
CNAG_01737	2.400905891	0.000513731006	0.01159998532
CNAG_13024	2.398446916	0.002025087491	0.03173433226
CNAG_04659	2.394012053	0.0005057469739	0.01148768126
CNAG_06121	2.376533107	0.0002608787366	0.006703792988
CNAG_12565	2.365774395	0.000159904645	0.004536774167
CNAG_05803	2.357683839	4.00E-11	7.44E-09
CNAG_05915	2.351445203	1.27E-15	6.08E-13
CNAG_02058	2.344566295	0.0003929159253	0.00948966564
CNAG_05167	2.341506748	0.0003923906963	0.00948966564
CNAG_13120	2.340033176	0.001087786356	0.02044430953
CNAG_02230	2.333653749	1.78E-06	0.0001067199916
CNAG_00546	2.3335204	6.94E-11	1.17E-08
CNAG_04351	2.30383649	0.002204341701	0.03364707172
CNAG_05818	2.287877612	8.45E-08	7.01E-06
CNAG_01348	2.285070697	4.27E-05	0.001537947384
CNAG_13017	2.2807982	0.0002439240754	0.006332069875
CNAG_03142	2.258014274	1.00E-08	1.06E-06
CNAG_00866	2.257173133	4.46E-09	5.41E-07
CNAG_13056	2.256249781	0.003533226996	0.04669449422
CNAG_03873	2.251635888	9.68E-07	6.48E-05

CNAG_07658	2.249610341	4.42E-05	0.001578100253
CNAG_06302	2.244962945	0.0001117740419	0.003385156698
CNAG_12604	2.232273916	2.28E-05	0.0009350592159
CNAG_12914	2.224377529	0.0001755191146	0.004853929941
CNAG_12841	2.220102927	0.003521721562	0.04669449422
CNAG_04139	2.214233899	6.19E-09	7.26E-07
CNAG_03566	2.206852362	0.0009844698488	0.01926531766
CNAG_06336	2.195307445	5.43E-10	7.68E-08
CNAG_01921	2.186658534	0.001714295311	0.02862910682
CNAG_12346	2.177490168	0.001891631598	0.03065166105
CNAG_01272	2.176814895	0.0004712371599	0.01096488416
CNAG_03227	2.176090785	1.60E-08	1.58E-06
CNAG_00454	2.173779784	0.0005081946231	0.01150902482
CNAG_00826	2.167506689	5.16E-05	0.001799743836
CNAG_12998	2.16577518	0.001540876413	0.02648641619
CNAG_02030	2.157188415	2.03E-15	8.59E-13
CNAG_05158	2.154314736	2.94E-15	1.18E-12
CNAG_04112	2.141863468	0.001994749754	0.03165058238
CNAG_12161	2.140394516	0.003055347281	0.04262963519
CNAG_04106	2.138358108	5.37E-05	0.001862879187
CNAG_06718	2.134979594	1.25E-05	0.0005680016399
CNAG_02297	2.125371335	0.001915249493	0.03096861045
CNAG_02118	2.120222152	0.0009371489924	0.0184814499
CNAG_00834	2.111704263	6.01E-05	0.002048461788

CNAG_12014	2.099083048	0.001373554745	0.0245969138
CNAG_13202	2.095618421	1.25E-05	0.0005680016399
CNAG_01585	2.080325261	0.0003386389407	0.008475274985
CNAG_03408	2.07327128	0.0004265490319	0.01014150222
CNAG_05097	2.07223777	0.001852269685	0.03020624409
CNAG_05138	2.070647824	5.66E-08	5.02E-06
CNAG_07943	2.066246576	8.90E-05	0.002842826011
CNAG_13042	2.060428222	0.003827715033	0.04868853523
CNAG_03154	2.048515805	4.09E-12	1.04E-09
CNAG_06075	2.034116982	0.003817676641	0.04864191674
CNAG_12199	2.026275561	0.003907448425	0.04926922479
CNAG_13146	2.01448894	1.04E-14	3.98E-12
CNAG_01751	1.993160981	0.0001295204063	0.003772899776
CNAG_00827	1.98649509	0.0004816056351	0.01110457464
CNAG_01691	1.967031489	0.001119279	0.02078427573
CNAG_00984	1.959516248	3.53E-11	7.10E-09
CNAG_06493	1.955778339	0.001376163809	0.0245969138
CNAG_13156	1.951894653	0.0001861297691	0.005055311025
CNAG_03268	1.948224648	0.0006618287706	0.01419264306
CNAG_03464	1.939420636	2.45E-13	8.13E-11
CNAG_06501	1.938398018	4.20E-10	6.29E-08
CNAG_05089	1.933323907	0.0003541819292	0.008756814912
CNAG_05891	1.93149386	0.00100800192	0.01941495269
CNAG_03051	1.923799935	1.08E-06	7.00E-05

CNAG_02768	1.919929164	0.003220754658	0.04436967428
CNAG_05458	1.915654781	1.38E-10	2.25E-08
CNAG_00961	1.909224741	0.003501723598	0.04660926208
CNAG_04094	1.909088615	2.32E-05	0.0009450080909
CNAG_12566	1.90786515	0.001283699524	0.02321610134
CNAG_12324	1.901175193	7.01E-05	0.00233606167
CNAG_04606	1.892635749	6.15E-10	8.54E-08
CNAG_04943	1.891878067	2.29E-10	3.64E-08
CNAG_04256	1.889285708	2.00E-05	0.0008453760181
CNAG_12377	1.887583104	0.003860861027	0.0490022937
CNAG_04737	1.887254207	1.02E-06	6.74E-05
CNAG_12600	1.887070318	0.0011575357	0.02139058707
CNAG_01121	1.875223876	5.35E-10	7.68E-08
CNAG_06936	1.872542048	0.001973398586	0.03157437737
CNAG_05889	1.871035642	7.78E-09	8.73E-07
CNAG_06658	1.871013454	4.39E-09	5.41E-07
CNAG_04744	1.869827078	0.0008568458202	0.01748515321
CNAG_03228	1.861316114	1.08E-08	1.13E-06
CNAG_04386	1.856599822	0.003007678925	0.0422749362
CNAG_03047	1.850272229	0.0001171158012	0.003505207038
CNAG_06759	1.847570401	4.30E-06	0.0002262713743
CNAG_00575	1.842674722	1.46E-12	3.85E-10
CNAG_00301	1.839185869	1.28E-05	0.0005750594008
CNAG_05251	1.835601975	0.002021684567	0.03173433226

CNAG_12573	1.83512486	0.002168169914	0.03329471385
CNAG_04691	1.823664198	2.10E-05	0.0008747115242
CNAG_06800	1.82336656	0.002937478902	0.04159339328
CNAG_04796	1.820594328	7.07E-11	1.17E-08
CNAG_06923	1.819513719	4.09E-05	0.001486414357
CNAG_12593	1.815639933	0.0002273392688	0.005982942412
CNAG_12987	1.813371212	0.0008972342611	0.0180677886
CNAG_06267	1.807429687	0.0006914918624	0.01465962748
CNAG_12370	1.804095322	0.0004785509094	0.0110675774
CNAG_13040	1.79961522	9.51E-05	0.003011027055
CNAG_05383	1.791990311	6.70E-05	0.002261361081
CNAG_01936	1.788502211	4.23E-05	0.001530411608
CNAG_01052	1.786541629	0.0003545408553	0.008756814912
CNAG_03688	1.783939109	0.002670372315	0.03875919855
CNAG_00595	1.783202077	0.001015011284	0.01941495269
CNAG_04981	1.777248592	7.40E-08	6.34E-06
CNAG_03998	1.773515218	0.0007095636823	0.01487296647
CNAG_00075	1.759296988	0.002948127873	0.04166687394
CNAG_06868	1.758013716	2.06E-05	0.0008636783382
CNAG_13200	1.739682516	2.14E-06	0.0001230541438
CNAG_01954	1.713386944	2.04E-06	0.000118640103
CNAG_05643	1.70878731	5.65E-05	0.001942819296
CNAG_03242	1.688110609	8.16E-05	0.002629074631
CNAG_00093	1.672161373	8.07E-05	0.002622294488

CNAG_12314	1.669481566	0.0003782177689	0.00922223007
CNAG_00663	1.662747191	0.0006461365199	0.01404932741
CNAG_12357	1.662204424	3.65E-06	0.0001959550232
CNAG_13021	1.661288148	0.0004999071983	0.01138893056
CNAG_00465	1.659918647	0.003667801022	0.04744518204
CNAG_02661	1.654970842	1.61E-05	0.00070822826
CNAG_07406	1.653535168	1.50E-06	9.25E-05
CNAG_02510	1.649479306	3.88E-11	7.40E-09
CNAG_03352	1.641591212	0.00378036588	0.04832789346
CNAG_00453	1.62949169	0.002494796695	0.0370433626
CNAG_06931	1.617386623	0.002605801405	0.03819542208
CNAG_06404	1.602386702	0.0009439755845	0.01856809706
CNAG_04938	1.595354764	4.51E-05	0.00159978446
CNAG_12426	1.588559348	0.0001800880283	0.004943999394
CNAG_04163	1.587164767	9.98E-05	0.003095808252
CNAG_13168	1.582096877	0.0004705559091	0.01096488416
CNAG_00264	1.567211417	0.002019239942	0.03173433226
CNAG_00807	1.565646772	5.40E-06	0.0002729832553
CNAG_05521	1.565334998	4.41E-07	3.14E-05
CNAG_04681	1.56052168	1.06E-05	0.0004943713953
CNAG_03161	1.554754653	1.87E-05	0.0007931934503
CNAG_06499	1.54425551	0.00148730112	0.02583362504
CNAG_02362	1.541730331	5.06E-05	0.001771578271
CNAG_02043	1.538264189	0.001225832343	0.02243537756

CNAG_02524	1.537876362	1.26E-08	1.28E-06
CNAG_06149	1.528795763	6.50E-10	8.85E-08
CNAG_03135	1.52430159	7.08E-08	6.14E-06
CNAG_01384	1.523894822	0.003284646134	0.04476503446
CNAG_01621	1.515694379	0.002741633947	0.03933588569
CNAG_06576	1.508379333	1.14E-06	7.29E-05
CNAG_00776	1.508366035	3.10E-10	4.73E-08
CNAG_12636	1.501554551	0.001418695215	0.02512176771
CNAG_12511	1.495105526	0.0006579502417	0.01418496114
CNAG_03698	1.493120172	0.003536182808	0.04669449422
CNAG_07450	1.490604493	0.002671296965	0.03875919855
CNAG_01087	1.490178597	3.20E-05	0.001221118423
CNAG_03824	1.481867535	0.003628076119	0.04721703096
CNAG_01675	1.474620873	0.002382698327	0.03565637967
CNAG_00932	1.461976743	0.003656432126	0.04737842103
CNAG_03874	1.46117082	0.002514268202	0.03725999013
CNAG_03705	1.458608351	0.002690591693	0.03881776143
CNAG_05159	1.449618639	3.26E-06	0.000181938655
CNAG_03667	1.449254632	0.003602454033	0.04707864585
CNAG_12363	1.446005132	0.002859837178	0.0407526258
CNAG_04076	1.445237097	0.001090391467	0.02044430953
CNAG_01939	1.437182548	0.00310790806	0.04320501697
CNAG_03232	1.435749293	0.0001027998214	0.003176389622
CNAG_02254	1.435721863	0.002652422633	0.03870609854

CNAG_02188	1.423877214	0.001710580871	0.02862910682
CNAG_04880	1.42166938	0.0002302977041	0.006039972775
CNAG_01743	1.402132702	0.003005214433	0.0422749362
CNAG_04874	1.394988492	0.001433215036	0.02526165625
CNAG_00036	1.394180103	0.0005773880302	0.01284730451
CNAG_03412	1.391736894	3.08E-10	4.73E-08
CNAG_00139	1.38320838	3.00E-05	0.001182764923
CNAG_12625	1.379960696	0.001529066854	0.02634274995
CNAG_02347	1.377144078	0.0008141458387	0.01683891881
CNAG_06748	1.376530337	5.53E-05	0.001910971149
CNAG_07574	1.37260772	0.0003420935892	0.008532216577
CNAG_03454	1.370957075	0.001553968109	0.02659166952
CNAG_07549	1.364825654	1.54E-06	9.43E-05
CNAG_01643	1.347617711	0.000345277551	0.008583577423
CNAG_05412	1.34443339	7.56E-05	0.002496474336
CNAG_01215	1.337276605	0.00203247997	0.03173433226
CNAG_00647	1.335253496	3.16E-05	0.001212352663
CNAG_02129	1.325368574	0.0005599848597	0.01253315088
CNAG_05731	1.323953134	8.03E-05	0.002620636917
CNAG_02602	1.320741253	0.0002484120655	0.006405002986
CNAG_00668	1.319534729	1.84E-06	0.0001090468253
CNAG_00115	1.297218628	6.87E-07	4.72E-05
CNAG_12109	1.29655948	0.002240870264	0.03400063988
CNAG_04326	1.294571224	3.07E-07	2.25E-05

CNAG_00522	1.291708334	0.001010043071	0.01941495269
CNAG_03019	1.286491458	0.0001566408705	0.004460757924
CNAG_01925	1.285788848	0.0009067148028	0.018162854
CNAG_01417	1.279003863	7.69E-05	0.002519396364
CNAG_06500	1.278874081	3.94E-05	0.001449562174
CNAG_03555	1.276940124	0.003865461335	0.0490022937
CNAG_02125	1.269574459	0.0006441011977	0.01404508669
CNAG_05386	1.268957205	4.65E-06	0.0002412717332
CNAG_03040	1.251563734	3.41E-05	0.001295529771
CNAG_03463	1.245773599	4.72E-06	0.0002433201573
CNAG_12794	1.244543258	0.0006620258031	0.01419264306
CNAG_02415	1.243199672	9.73E-08	7.91E-06
CNAG_05444	1.239084067	0.000241961607	0.006302563088
CNAG_04804	1.233738962	0.001772909357	0.02935107204
CNAG_06440	1.226922637	8.12E-06	0.0003896793402
CNAG_04440	1.226537718	2.46E-06	0.0001398558031
CNAG_06081	1.224851305	0.0002325816221	0.006078982671
CNAG_03314	1.220558626	3.56E-06	0.0001938543309
CNAG_05662	1.219153834	0.003633423838	0.04721703096
CNAG_00515	1.215223113	0.001030316967	0.01953913255
CNAG_01174	1.202010259	0.0009023560688	0.01812310926
CNAG_00130	1.198771159	0.0001888603124	0.005111283348
CNAG_04658	1.198229096	0.001796654495	0.02961569569
CNAG_01476	1.197942446	0.0008724759438	0.01770940533

CNAG_07511	1.195333681	3.01E-05	0.001182764923
CNAG_00025	1.183007384	1.77E-05	0.0007650884747
CNAG_02586	1.182814164	0.003070005036	0.04275598254
CNAG_06901	1.181279988	0.001601573185	0.02710245353
CNAG_00692	1.180924207	0.001172744519	0.02161929026
CNAG_05520	1.17820997	0.0007137533935	0.01488351339
CNAG_01232	1.177652494	2.11E-05	0.0008747115242
CNAG_06453	1.174618692	0.002890840454	0.04100909729
CNAG_02933	1.172938973	0.001236207847	0.02253568509
CNAG_04634	1.171311508	0.002643474681	0.03864942292
CNAG_03771	1.170014948	0.002547582263	0.03768051906
CNAG_03316	1.169623614	3.06E-05	0.001196542274
CNAG_02226	1.162666652	3.87E-06	0.0002066824748
CNAG_00849	1.147536502	0.0001042920551	0.003189220622
CNAG_04478	1.144351266	3.44E-05	0.001299970672
CNAG_02102	1.14431557	0.001461240491	0.02557841153
CNAG_06311	1.143464503	0.0002138915037	0.00564851196
CNAG_05031	1.141004879	0.002228913153	0.03395422193
CNAG_02424	1.128078622	0.002198558233	0.03364707172
CNAG_07641	1.125384069	0.0006721046453	0.0143282197
CNAG_02722	1.121250966	0.0001216474394	0.003570820222
CNAG_03194	1.120669625	0.0004091490564	0.0098387055
CNAG_00249	1.118057964	0.001591506306	0.02706295326
CNAG_00074	1.115805361	5.86E-05	0.002004026834

CNAG_01379	1.113896979	0.003536349274	0.04669449422
CNAG_08019	1.112490086	0.003306722446	0.04494303315
CNAG_06614	1.112231235	0.001137915701	0.02107905978
CNAG_02796	1.109678832	0.001881874659	0.03062359786
CNAG_05652	1.102938628	0.001001347752	0.01941495269
CNAG_01252	1.102119268	0.0003318012592	0.008357449539
CNAG_06521	1.100146086	0.0005679307953	0.01267382406
CNAG_04920	1.094710124	0.001673030845	0.02812460663
CNAG_02693	1.093215696	0.003320235054	0.04500223082
CNAG_01913	1.084625568	0.002562759082	0.03775864347
CNAG_02684	1.079859211	0.0001548584135	0.00442651465
CNAG_07687	1.077441356	0.0002456944364	0.006356406571
CNAG_01131	1.074525662	0.003274374547	0.04476503446
CNAG_02605	1.065598576	0.001013648464	0.01941495269
CNAG_05774	1.055557934	0.0009506120293	0.01865056814
CNAG_02000	1.053860267	0.0009911794803	0.01929765764
CNAG_13206	1.052609763	0.001695046326	0.02843207375
CNAG_00923	1.049121628	3.54E-05	0.001331635048
CNAG_04388	1.044628772	0.0001384402211	0.004002180938
CNAG_02606	1.041468486	0.002130812456	0.03285325387
CNAG_01821	1.038494068	0.003975744569	0.04974243041
CNAG_02114	1.036116492	0.0001044687049	0.003189220622
CNAG_04621	1.033260484	0.002666454014	0.03875919855
CNAG_05229	1.027875736	0.002204321466	0.03364707172

CNAG_07522	1.017506147	0.002587882098	0.03805532981
CNAG_05615	1.014900441	0.0006084668406	0.01346034472
CNAG_01261	1.010040466	0.0001125957517	0.003391549936
CNAG_05183	-1.013336194	0.00108309155	0.02041025854
CNAG_00482	-1.025120463	0.001990107825	0.03164271442
CNAG_03913	-1.032340298	0.0004099460625	0.0098387055
CNAG_07426	-1.040986853	0.001772697926	0.02935107204
CNAG_02886	-1.052187626	0.002862081686	0.0407526258
CNAG_02777	-1.052887379	0.002033292515	0.03173433226
CNAG_02935	-1.054604426	0.0001931030425	0.005171096212
CNAG_05194	-1.058389217	0.0001326459129	0.00384925326
CNAG_00936	-1.064291747	0.0009218244613	0.01836909736
CNAG_04906	-1.075064757	0.003495841782	0.04660926208
CNAG_05618	-1.081957718	0.0008130262653	0.01683891881
CNAG_02725	-1.106138269	0.0006510742988	0.01411647457
CNAG_05227	-1.116646261	0.0004682068184	0.01096120993
CNAG_01442	-1.123566209	0.0004852717368	0.0111554033
CNAG_00309	-1.126731237	1.86E-05	0.0007931934503
CNAG_01180	-1.130061477	3.11E-05	0.001201539803
CNAG_06031	-1.133011507	0.001649720701	0.02785546104
CNAG_00627	-1.137308805	0.0004354640665	0.01028935528
CNAG_04677	-1.137313587	0.001486375372	0.02583362504
CNAG_07445	-1.141274504	7.67E-05	0.002519396364
CNAG_06624	-1.14704725	2.69E-05	0.001072881566

CNAG_05761	-1.150828445	0.003874669704	0.0490022937
CNAG_04414	-1.152015132	0.0001204126046	0.003561337861
CNAG_03540	-1.15247079	0.0006317750526	0.01385548046
CNAG_06583	-1.152602229	0.0003163188388	0.008020416536
CNAG_06764	-1.158644584	0.00301313857	0.0422749362
CNAG_04269	-1.168131851	4.39E-07	3.14E-05
CNAG_02531	-1.168548748	0.001836869431	0.03008366416
CNAG_02735	-1.178151337	0.00395721041	0.04959183883
CNAG_00062	-1.17929381	0.00152776083	0.02634274995
CNAG_07626	-1.198193915	0.002059937356	0.03208457531
CNAG_05590	-1.201377237	0.001031141133	0.01953913255
CNAG_00076	-1.207786431	0.00350546599	0.04660926208
CNAG_01538	-1.215717303	0.003689352301	0.04756273101
CNAG_01864	-1.23342153	0.0008294109245	0.01702498408
CNAG_05738	-1.236977017	0.003382496088	0.04560991192
CNAG_01627	-1.241052253	1.62E-06	9.79E-05
CNAG_05591	-1.252054574	0.002491467957	0.0370433626
CNAG_12497	-1.26729711	0.001819088085	0.0298565167
CNAG_03599	-1.284917879	0.001658021941	0.02793382661
CNAG_06760	-1.288103298	0.003712606792	0.04770137211
CNAG_04467	-1.28881782	0.0001089330695	0.003312259708
CNAG_06876	-1.289400796	9.74E-06	0.0004616745729
CNAG_00332	-1.298651098	0.001800980371	0.02962302197
CNAG_05329	-1.312722314	0.0004424624933	0.01039038077

CNAG_03061	-1.323514383	0.0002627377164	0.006728906885
CNAG_01749	-1.32674026	0.0009911431082	0.01929765764
CNAG_02309	-1.334621089	0.0007112988417	0.01487296647
CNAG_01055	-1.336851907	0.0004771296431	0.0110675774
CNAG_00176	-1.35030752	0.0001630244234	0.004578890824
CNAG_00194	-1.355385937	4.94E-06	0.0002531456025
CNAG_06141	-1.356252394	0.000329606482	0.008329657851
CNAG_04313	-1.367890822	0.003426642962	0.0461237021
CNAG_06777	-1.368103846	9.64E-05	0.003015919143
CNAG_05457	-1.374602668	1.82E-06	0.0001086783764
CNAG_04307	-1.374925979	0.0008336800246	0.01705803203
CNAG_03394	-1.375892582	1.18E-05	0.0005437822571
CNAG_04707	-1.390010717	0.0008605890182	0.0175147077
CNAG_06356	-1.39429867	0.00101321828	0.01941495269
CNAG_01565	-1.395005051	0.0001843233249	0.005024127198
CNAG_06628	-1.406163629	1.05E-06	6.93E-05
CNAG_01264	-1.407510527	0.003704355957	0.04767562338
CNAG_06690	-1.415584444	9.61E-06	0.0004583901762
CNAG_06166	-1.416891527	3.27E-06	0.000181938655
CNAG_05653	-1.422462087	0.0006384173609	0.01396103524
CNAG_06634	-1.42664409	0.002292103401	0.03456114714
CNAG_06250	-1.426906427	7.63E-06	0.0003731608612
CNAG_00919	-1.431200158	1.08E-06	7.00E-05
CNAG_03912	-1.444777578	0.003550467976	0.04679995094

CNAG_00550	-1.45977442	9.55E-09	1.03E-06
CNAG_01577	-1.461654885	0.003637790121	0.04721703096
CNAG_05515	-1.489375092	0.002295925262	0.03456114714
CNAG_02192	-1.493561354	9.75E-05	0.00303704795
CNAG_00758	-1.502679486	3.12E-06	0.0001765329596
CNAG_00190	-1.506843396	4.14E-06	0.0002192489526
CNAG_01118	-1.519131979	0.002251826978	0.03409909424
CNAG_01879	-1.51925901	2.38E-09	3.03E-07
CNAG_01026	-1.519273819	6.43E-05	0.00218086053
CNAG_00699	-1.520429106	0.001332651883	0.02404444248
CNAG_03849	-1.537900219	5.03E-05	0.001767319021
CNAG_02882	-1.550029147	0.003484281748	0.04660926208
CNAG_01562	-1.552043821	0.001031744028	0.01953913255
CNAG_03949	-1.553959377	0.003280254428	0.04476503446
CNAG_05130	-1.557956551	5.96E-11	1.06E-08
CNAG_01977	-1.573075466	0.002557882531	0.03775864347
CNAG_12615	-1.578264998	1.07E-07	8.57E-06
CNAG_04758	-1.581679436	0.0009278175102	0.01839247594
CNAG_07548	-1.582932303	0.002012629077	0.03173433226
CNAG_01443	-1.583145324	1.05E-05	0.0004943713953
CNAG_05602	-1.590259638	0.0001919992722	0.005159642413
CNAG_03086	-1.591627144	0.000146293924	0.004197425668
CNAG_05548	-1.653009091	0.0009255798812	0.01839247594
CNAG_05129	-1.666964748	3.12E-05	0.001201539803

CNAG_00904	-1.66972394	0.002237423834	0.03400063988
CNAG_02100	-1.679770189	0.0001761711994	0.004853929941
CNAG_00601	-1.687452692	1.87E-05	0.0007931934503
CNAG_03341	-1.715096533	0.003333243834	0.04502533972
CNAG_00792	-1.718315712	2.97E-05	0.001179269109
CNAG_06568	-1.734800643	0.0003610053063	0.008859139863
CNAG_00177	-1.750694988	0.0003672267903	0.008982932254
CNAG_02705	-1.753871306	6.93E-07	4.72E-05
CNAG_05637	-1.764489245	0.0003604390041	0.008859139863
CNAG_00791	-1.796202438	3.10E-05	0.001201539803
CNAG_00796	-1.812225779	1.81E-08	1.77E-06
CNAG_00141	-1.813749606	8.20E-05	0.002629074631
CNAG_05514	-1.843046871	0.001195747059	0.02193735951
CNAG_04523	-1.865609695	0.0008298341297	0.01702498408
CNAG_01004	-1.868370284	5.90E-07	4.13E-05
CNAG_06000	-1.900787416	9.23E-09	1.01E-06
CNAG_02389	-1.908080708	0.001415095511	0.02511629986
CNAG_07909	-1.909016666	0.00346665491	0.04649826058
CNAG_00236	-1.910766678	5.99E-07	4.16E-05
CNAG_01244	-1.91345166	0.00285523492	0.0407526258
CNAG_07917	-1.920440986	7.74E-06	0.0003763268081
CNAG_06610	-1.927182646	4.58E-06	0.0002394986925
CNAG_05115	-1.935278094	0.0001735180669	0.004850878706
CNAG_03772	-1.944995715	2.80E-11	5.93E-09

CNAG_00798	-1.98081874	0.001368956448	0.02458323673
CNAG_05264	-2.033205263	0.001758754633	0.02930745712
CNAG_05759	-2.077721686	9.63E-05	0.003015919143
CNAG_01993	-2.125796405	0.00159275968	0.02706295326
CNAG_05913	-2.28456034	2.56E-07	1.92E-05
CNAG_00474	-2.423263857	4.01E-05	0.001464809608
CNAG_06963	-2.630361087	0.0002022304948	0.005359108112
CNAG_00164	-2.733630974	3.14E-08	2.93E-06
**DEGs for WT vs *pmc1*Δ**			
**Gene ID**	**Log_2FoldChange**	**p-value**	**p-adj**
CNAG_06346	4.923407258	6.58E-43	2.93E-40
CNAG_06623	4.454868621	3.65E-95	2.12E-91
CNAG_03084	3.468027125	0.000166991753	0.001479689922
CNAG_01534	3.465750175	0.0001336533291	0.001225507978
CNAG_01761	3.279999283	3.97E-80	7.66E-77
CNAG_12392	3.223877182	2.39E-05	0.0002749610087
CNAG_04585	3.166149986	1.08E-18	8.97E-17
CNAG_13120	3.154576335	4.68E-05	0.0004924106755
CNAG_01562	2.975912228	4.42E-78	6.40E-75
CNAG_05641	2.83448109	0.0001680714615	0.001484598355
CNAG_06576	2.811705832	6.32E-59	7.32E-56
CNAG_06302	2.79737781	4.96E-05	0.0005189779288
CNAG_02323	2.772260187	2.47E-10	7.23E-09
CNAG_00575	2.768111366	1.92E-07	3.41E-06

CNAG_00462	2.687897164	9.14E-18	6.88E-16
CNAG_06267	2.581460167	9.27E-23	1.41E-20
CNAG_04951	2.572418447	1.60E-26	3.31E-24
CNAG_06347	2.516501013	1.84E-51	1.18E-48
CNAG_05473	2.497037582	0.001823993744	0.01112636184
CNAG_01558	2.478349322	7.99E-24	1.36E-21
CNAG_00588	2.469524081	4.44E-12	1.72E-10
CNAG_03716	2.448763517	0.0002025758983	0.001734013783
CNAG_01621	2.392417513	1.28E-35	4.64E-33
CNAG_05265	2.279928615	1.54E-08	3.43E-07
CNAG_03644	2.271502751	3.66E-07	6.06E-06
CNAG_00539	2.254578459	2.13E-07	3.76E-06
CNAG_06220	2.231330815	1.03E-55	9.97E-53
CNAG_03295	2.21476473	2.58E-09	6.53E-08
CNAG_03759	2.159458626	2.63E-06	3.72E-05
CNAG_04963	2.159404023	2.26E-15	1.25E-13
CNAG_00699	2.134408497	4.67E-08	9.59E-07
CNAG_01683	2.038511994	6.95E-16	4.15E-14
CNAG_02235	2.030370602	0.009837423433	0.04506550893
CNAG_05586	2.006425417	0.0009798819022	0.006639048506
CNAG_05115	2.003276524	4.17E-17	2.91E-15
CNAG_02044	1.994561784	2.04E-11	6.93E-10
CNAG_02204	1.967122972	1.32E-05	0.000160870949
CNAG_05590	1.966338044	6.29E-21	7.14E-19

CNAG_00605	1.957814395	5.36E-08	1.08E-06
CNAG_06993	1.94346378	0.000340174131	0.002693045203
CNAG_03524	1.93365808	1.86E-09	4.82E-08
CNAG_00613	1.928446523	0.000540339204	0.003993961336
CNAG_03302	1.913738007	2.93E-05	0.0003306906686
CNAG_07979	1.893406348	0.0002699638664	0.00223172697
CNAG_01908	1.891165931	0.0008123725173	0.005666271829
CNAG_00669	1.889843236	3.66E-05	0.0004065345012
CNAG_05401	1.886919003	0.003578210712	0.01970476726
CNAG_03878	1.88125517	6.38E-05	0.0006451328631
CNAG_04981	1.876388221	4.90E-14	2.35E-12
CNAG_01138	1.853405655	3.25E-21	3.93E-19
CNAG_01915	1.832649519	2.50E-13	1.12E-11
CNAG_04052	1.829810925	0.009903839703	0.04522205712
CNAG_02942	1.829665988	7.42E-05	0.0007312407393
CNAG_04680	1.822799586	0.0002050185024	0.001749752903
CNAG_06205	1.821125123	3.23E-14	1.60E-12
CNAG_01810	1.81534856	0.001751014457	0.01076047591
CNAG_01239	1.811638762	0.000450434896	0.003407663476
CNAG_05789	1.80356996	7.93E-06	0.000100803826
CNAG_00480	1.797776742	0.0006761400808	0.004849296743
CNAG_06634	1.792038949	0.004004950889	0.02164989776
CNAG_07797	1.786491193	0.0001666369629	0.001478807351
CNAG_03859	1.773382244	8.45E-08	1.61E-06

CNAG_02527	1.760928796	2.22E-13	1.01E-11
CNAG_04068	1.750891029	1.23E-12	5.04E-11
CNAG_06109	1.749487719	3.94E-09	9.81E-08
CNAG_07752	1.745531523	2.02E-07	3.58E-06
CNAG_05939	1.741517159	1.05E-26	2.26E-24
CNAG_06396	1.739084985	2.50E-12	9.99E-11
CNAG_01629	1.738856067	0.0002457910963	0.002058322837
CNAG_06447	1.737068466	1.31E-25	2.54E-23
CNAG_05875	1.728675207	4.02E-05	0.0004375288417
CNAG_00399	1.724731907	7.30E-05	0.0007233754913
CNAG_00716	1.72300079	2.71E-29	7.48E-27
CNAG_06574	1.708738465	0.000470591141	0.003537063116
CNAG_03817	1.708159849	6.99E-10	1.90E-08
CNAG_04776	1.705894463	4.02E-08	8.40E-07
CNAG_04076	1.70484181	0.00185898644	0.01130829709
CNAG_01102	1.694242451	9.16E-14	4.26E-12
CNAG_07672	1.691788393	1.76E-06	2.58E-05
CNAG_03310	1.6873339	0.001392585863	0.008868170411
CNAG_03772	1.681713786	1.47E-30	4.26E-28
CNAG_01390	1.680750944	4.01E-07	6.55E-06
CNAG_06112	1.680634602	4.80E-15	2.60E-13
CNAG_01628	1.672603902	3.41E-19	3.09E-17
CNAG_04753	1.668329343	0.0008325985851	0.005785262351
CNAG_00895	1.654548909	1.51E-10	4.55E-09

CNAG_03975	1.651557287	5.77E-08	1.13E-06
CNAG_03142	1.648753586	4.00E-13	1.73E-11
CNAG_00735	1.639525809	0.0007464377075	0.005300988376
CNAG_06777	1.638198598	0.007080730582	0.03468540467
CNAG_04807	1.637283927	0.0008565112088	0.005894872275
CNAG_04348	1.635371172	1.20E-12	4.98E-11
CNAG_12913	1.634479612	4.65E-06	6.21E-05
CNAG_06344	1.632312223	0.0001392089128	0.001268420833
CNAG_03993	1.630932164	0.01045693832	0.04715794365
CNAG_06774	1.629761485	1.70E-05	0.0002018507873
CNAG_04206	1.629280067	6.86E-09	1.62E-07
CNAG_04043	1.617777316	0.006239814306	0.03103838962
CNAG_05660	1.608005508	0.001482590542	0.009348870717
CNAG_04762	1.607757516	4.50E-29	1.19E-26
CNAG_01846	1.600605608	5.65E-08	1.12E-06
CNAG_07939	1.595181768	1.52E-07	2.77E-06
CNAG_04347	1.592900329	4.31E-07	6.98E-06
CNAG_04322	1.585484597	1.22E-17	9.10E-16
CNAG_05387	1.576099753	1.51E-22	2.24E-20
CNAG_01348	1.573930553	0.0002601004131	0.002165634905
CNAG_05725	1.559581666	8.60E-28	2.08E-25
CNAG_02405	1.559401416	0.000269281913	0.002230815052
CNAG_03578	1.554653971	0.01062398723	0.04772558606
CNAG_05145	1.554451256	0.001687074842	0.01044508409

CNAG_01535	1.553661312	5.42E-08	1.09E-06
CNAG_02546	1.538380697	1.10E-07	2.06E-06
CNAG_04862	1.53517841	1.08E-06	1.64E-05
CNAG_04934	1.533956074	4.06E-09	1.01E-07
CNAG_06659	1.53154784	0.0002526187803	0.002109403216
CNAG_01639	1.520898931	2.21E-07	3.89E-06
CNAG_05459	1.520230268	1.10E-13	5.02E-12
CNAG_04448	1.518326252	1.93E-20	2.00E-18
CNAG_00070	1.507206962	1.06E-07	1.99E-06
CNAG_05497	1.503347254	1.94E-11	6.66E-10
CNAG_06334	1.500901739	0.007352344531	0.0358028365
CNAG_04635	1.499812876	1.31E-08	2.95E-07
CNAG_00779	1.499449183	1.20E-15	6.90E-14
CNAG_06684	1.491078997	0.0002694685999	0.002230815052
CNAG_01152	1.4886422	2.63E-20	2.67E-18
CNAG_03624	1.488539808	3.51E-11	1.15E-09
CNAG_00771	1.468898637	7.28E-17	4.96E-15
CNAG_00838	1.468839561	0.003973684219	0.02152102808
CNAG_02129	1.46543971	3.83E-13	1.67E-11
CNAG_03293	1.464870455	4.24E-06	5.69E-05
CNAG_00674	1.460673749	0.0005271796121	0.003916674169
CNAG_01745	1.456999593	6.90E-06	8.83E-05
CNAG_00700	1.456218448	9.18E-12	3.26E-10
CNAG_05884	1.453541735	4.58E-16	2.76E-14

CNAG_07413	1.4524197	4.16E-09	1.03E-07
CNAG_03510	1.451685177	0.000109834928	0.001023301299
CNAG_05085	1.448651255	2.10E-06	3.00E-05
CNAG_04004	1.447421723	3.27E-20	3.27E-18
CNAG_02418	1.445733174	2.08E-08	4.56E-07
CNAG_03268	1.444974583	4.40E-09	1.08E-07
CNAG_03221	1.443751007	8.39E-15	4.50E-13
CNAG_06535	1.443506105	3.48E-10	9.84E-09
CNAG_04744	1.440407902	1.75E-05	0.0002069900864
CNAG_05525	1.435217097	1.77E-14	9.09E-13
CNAG_07629	1.434173198	0.01065729604	0.04783813364
CNAG_00603	1.430350182	3.84E-06	5.25E-05
CNAG_02070	1.42792542	1.62E-07	2.93E-06
CNAG_07362	1.427644521	1.78E-17	1.31E-15
CNAG_06404	1.426912769	2.80E-16	1.74E-14
CNAG_05598	1.422150378	0.001064195075	0.007121259193
CNAG_00995	1.420106149	5.25E-05	0.0005452409621
CNAG_02328	1.419113989	2.09E-08	4.58E-07
CNAG_04445	1.418454322	2.74E-23	4.54E-21
CNAG_04953	1.416557375	3.33E-11	1.10E-09
CNAG_00116	1.41131847	1.19E-19	1.13E-17
CNAG_03390	1.410462217	0.0007779103426	0.005470862179
CNAG_06541	1.407337615	2.41E-14	1.22E-12
CNAG_00992	1.406940582	7.08E-16	4.19E-14

CNAG_02815	1.40581077	3.97E-07	6.50E-06
CNAG_01486	1.402231297	1.80E-15	1.00E-13
CNAG_07917	1.401613474	3.91E-07	6.44E-06
CNAG_04365	1.398410965	6.04E-08	1.17E-06
CNAG_06095	1.396838094	8.68E-23	1.36E-20
CNAG_03675	1.396731593	5.56E-08	1.11E-06
CNAG_01277	1.394416225	1.17E-05	0.0001436883651
CNAG_02330	1.394243137	1.38E-21	1.78E-19
CNAG_04755	1.391866508	1.52E-06	2.27E-05
CNAG_05800	1.390555014	1.32E-16	8.61E-15
CNAG_07356	1.388342964	6.32E-08	1.22E-06
CNAG_05309	1.3818296	0.0001181261876	0.001096919887
CNAG_04799	1.381756177	1.73E-16	1.12E-14
CNAG_01300	1.380528483	7.03E-11	2.20E-09
CNAG_04356	1.380425878	0.0006135667807	0.00446125407
CNAG_05371	1.379837644	0.0001278726413	0.001174361262
CNAG_03300	1.377088098	7.27E-05	0.0007213294651
CNAG_02378	1.375249189	3.61E-09	9.01E-08
CNAG_06350	1.371435536	0.0008494091507	0.005873897409
CNAG_00643	1.371386372	0.004868760017	0.025349923
CNAG_03476	1.370997916	1.18E-09	3.17E-08
CNAG_03566	1.370467491	4.68E-05	0.0004924106755
CNAG_04323	1.370127032	4.54E-05	0.0004831627485
CNAG_06741	1.369407995	3.74E-08	7.85E-07

CNAG_05402	1.366306458	9.96E-07	1.53E-05
CNAG_01428	1.364400407	1.42E-20	1.52E-18
CNAG_03333	1.364332936	1.40E-05	0.0001696014513
CNAG_01148	1.363611581	5.57E-12	2.11E-10
CNAG_03897	1.361923048	5.56E-06	7.29E-05
CNAG_02060	1.360846691	0.008507713042	0.03988850896
CNAG_06379	1.35691903	3.00E-10	8.61E-09
CNAG_06611	1.355282986	0.0001683142569	0.001484598355
CNAG_00513	1.351051787	1.46E-05	0.000175297517
CNAG_05814	1.348326433	4.20E-18	3.20E-16
CNAG_04219	1.347175257	4.23E-06	5.69E-05
CNAG_04373	1.342935398	4.08E-06	5.51E-05
CNAG_06063	1.339382011	0.0004256258974	0.003245397468
CNAG_07429	1.339202154	0.001504159598	0.009474570514
CNAG_00672	1.338755531	3.32E-18	2.60E-16
CNAG_03322	1.336415237	1.34E-11	4.66E-10
CNAG_04098	1.335897045	2.98E-07	5.05E-06
CNAG_01712	1.332626526	0.00349080077	0.01928426164
CNAG_07965	1.328585832	6.76E-20	6.53E-18
CNAG_04370	1.32847718	1.95E-06	2.82E-05
CNAG_00259	1.328233008	0.0003772191481	0.002938151832
CNAG_06770	1.327989069	3.03E-23	4.88E-21
CNAG_01598	1.326634975	7.30E-06	9.32E-05
CNAG_03397	1.321109016	0.0005449888408	0.004018989195

CNAG_00522	1.321007406	7.36E-05	0.0007266338588
CNAG_03263	1.320098206	3.03E-09	7.62E-08
CNAG_02088	1.318600466	0.0001079532336	0.001010644569
CNAG_01881	1.318554441	1.71E-05	0.0002027474219
CNAG_05762	1.315171124	1.53E-20	1.61E-18
CNAG_00656	1.314936911	6.05E-19	5.08E-17
CNAG_00192	1.311541701	1.12E-05	0.0001385696234
CNAG_00370	1.307051382	9.62E-15	5.07E-13
CNAG_05905	1.306992108	0.007025977867	0.03444631281
CNAG_04334	1.306108758	0.0002427042758	0.002035414296
CNAG_00930	1.304520152	1.20E-15	6.90E-14
CNAG_01043	1.29470699	6.87E-12	2.52E-10
CNAG_03679	1.294036399	0.0007994361543	0.005601853101
CNAG_00906	1.293382	3.32E-06	4.60E-05
CNAG_05465	1.292891825	2.72E-19	2.50E-17
CNAG_00821	1.291576642	4.34E-16	2.65E-14
CNAG_01153	1.291203462	2.95E-21	3.64E-19
CNAG_04513	1.29092479	4.46E-05	0.0004763610862
CNAG_00721	1.289979011	8.89E-07	1.38E-05
CNAG_06144	1.289974701	3.30E-13	1.45E-11
CNAG_00640	1.2870475	5.45E-06	7.17E-05
CNAG_06182	1.282320928	0.002087831524	0.01249895008
CNAG_05176	1.281517249	0.007556792629	0.03655393429
CNAG_00334	1.28048988	1.03E-25	2.07E-23

CNAG_04072	1.279000249	6.54E-06	8.42E-05
CNAG_03747	1.276130992	8.49E-22	1.20E-19
CNAG_03789	1.274557512	5.92E-07	9.33E-06
CNAG_03113	1.271382144	3.15E-09	7.90E-08
CNAG_05571	1.27116213	6.25E-06	8.08E-05
CNAG_06538	1.26837037	0.0005778043124	0.004233092276
CNAG_06138	1.267317742	8.76E-05	0.0008450923824
CNAG_00997	1.26543283	0.007262752467	0.03542731528
CNAG_06949	1.265275251	0.001712670012	0.01058094107
CNAG_06472	1.264948415	2.86E-07	4.88E-06
CNAG_04049	1.262848739	0.007581417509	0.03658144418
CNAG_02986	1.259184701	0.0005989114363	0.004371148329
CNAG_05232	1.257747417	3.59E-18	2.77E-16
CNAG_01976	1.255562965	4.46E-09	1.08E-07
CNAG_00609	1.254831455	0.001118064721	0.007438788814
CNAG_01232	1.254421243	2.98E-06	4.17E-05
CNAG_03667	1.254024207	5.98E-09	1.42E-07
CNAG_00232	1.252878195	2.26E-11	7.62E-10
CNAG_03438	1.250974272	1.16E-21	1.53E-19
CNAG_01179	1.250496871	1.12E-05	0.0001385696234
CNAG_04114	1.248917494	2.72E-13	1.21E-11
CNAG_06605	1.248371195	6.96E-17	4.80E-15
CNAG_03856	1.248267796	4.40E-12	1.71E-10
CNAG_01140	1.247894056	4.56E-05	0.0004843951602

CNAG_04726	1.247038316	3.12E-18	2.48E-16
CNAG_01884	1.243857522	2.50E-17	1.81E-15
CNAG_01480	1.240763458	1.10E-13	5.02E-12
CNAG_06005	1.23674086	2.83E-05	0.0003196549247
CNAG_03577	1.236376054	1.40E-18	1.12E-16
CNAG_04694	1.230717461	4.88E-08	9.96E-07
CNAG_01890	1.230075851	3.79E-20	3.72E-18
CNAG_06231	1.228999255	1.36E-14	7.10E-13
CNAG_06468	1.226090127	2.09E-12	8.51E-11
CNAG_06314	1.225263916	3.69E-06	5.06E-05
CNAG_04079	1.224486975	0.01119817949	0.04976491574
CNAG_06948	1.223996199	6.88E-05	0.000691154317
CNAG_00707	1.223919531	2.25E-07	3.91E-06
CNAG_06633	1.222739543	0.0001526272792	0.001375041465
CNAG_07878	1.2209925	0.006749402292	0.03328747769
CNAG_01990	1.220864026	1.33E-18	1.09E-16
CNAG_01575	1.219944676	3.83E-05	0.0004234591069
CNAG_01224	1.217446449	3.85E-19	3.38E-17
CNAG_02208	1.21612038	3.44E-05	0.0003837460827
CNAG_00034	1.215968821	1.43E-11	4.97E-10
CNAG_07801	1.215644546	0.0008619072451	0.005917953181
CNAG_03198	1.213719925	5.42E-19	4.62E-17
CNAG_05904	1.212009063	3.50E-17	2.47E-15
CNAG_05069	1.211398202	6.64E-05	0.0006705761061

CNAG_06318	1.207747114	0.0005382043081	0.003983261769
CNAG_00417	1.205078182	2.05E-14	1.04E-12
CNAG_00891	1.204818649	3.00E-13	1.33E-11
CNAG_00874	1.204486505	3.36E-07	5.63E-06
CNAG_01170	1.201166935	4.43E-19	3.83E-17
CNAG_03098	1.200214748	0.005625740038	0.02842298476
CNAG_04730	1.197305786	0.0008659022905	0.005938347661
CNAG_05149	1.19607044	0.0002846474613	0.00232328456
CNAG_01332	1.194526089	3.69E-12	1.44E-10
CNAG_04021	1.193668373	9.28E-11	2.86E-09
CNAG_06222	1.19269182	7.67E-12	2.80E-10
CNAG_06033	1.188706911	0.001657303393	0.01030479953
CNAG_06543	1.188571924	0.0007665952696	0.005410986099
CNAG_00099	1.186567295	0.002129281411	0.01266856856
CNAG_04146	1.18615799	0.001320740202	0.008494660901
CNAG_02064	1.18603269	0.0001344569121	0.001230928603
CNAG_06568	1.183298114	9.99E-09	2.31E-07
CNAG_05662	1.181338291	9.38E-15	4.99E-13
CNAG_07625	1.180668888	6.04E-06	7.86E-05
CNAG_02212	1.179453362	1.43E-09	3.80E-08
CNAG_01715	1.178714014	4.02E-06	5.45E-05
CNAG_00702	1.172363944	2.06E-09	5.30E-08
CNAG_04628	1.172164638	1.97E-06	2.84E-05
CNAG_04236	1.171475043	0.00045642908	0.00344850915

CNAG_01049	1.170132411	0.0001549150954	0.001389679533
CNAG_03378	1.168948726	4.99E-05	0.000520891476
CNAG_04298	1.167231558	4.28E-05	0.0004614842365
CNAG_01236	1.167034788	6.24E-06	8.08E-05
CNAG_01600	1.166777931	0.0003396202613	0.002693045203
CNAG_00559	1.166362805	0.006052362926	0.03023572686
CNAG_01641	1.165884835	4.99E-06	6.60E-05
CNAG_00984	1.162997033	2.18E-12	8.79E-11
CNAG_01608	1.160641961	0.003335030727	0.01863693642
CNAG_06517	1.159788793	3.90E-05	0.0004277867607
CNAG_04376	1.159527696	0.003411796698	0.01897840106
CNAG_05234	1.159463437	0.0010053751	0.006789092148
CNAG_06377	1.159361175	7.08E-21	7.89E-19
CNAG_02234	1.157562297	2.69E-16	1.70E-14
CNAG_03301	1.156838082	0.0003259098798	0.002608629493
CNAG_00722	1.153059384	0.009277054595	0.04293972155
CNAG_00866	1.151827668	0.0003558020128	0.002797656261
CNAG_04011	1.151228102	1.52E-09	3.99E-08
CNAG_00130	1.15121992	2.65E-07	4.52E-06
CNAG_00265	1.150977392	0.007714391197	0.03700736506
CNAG_02301	1.150868802	8.49E-09	1.98E-07
CNAG_06248	1.147627776	3.38E-07	5.64E-06
CNAG_06889	1.147563247	2.42E-05	0.00027760798
CNAG_01951	1.144178201	8.61E-12	3.08E-10

CNAG_01812	1.143063062	5.75E-06	7.52E-05
CNAG_00442	1.142159264	0.0002819672024	0.002304654355
CNAG_05994	1.141839537	0.0008531667844	0.005878836522
CNAG_06800	1.138444641	0.0003557193904	0.002797656261
CNAG_00268	1.136559005	5.38E-09	1.29E-07
CNAG_03603	1.136220347	2.42E-05	0.00027760798
CNAG_03246	1.133221343	0.01054692707	0.04748985419
CNAG_05264	1.132847563	0.0001569201552	0.001396854531
CNAG_05555	1.129007797	1.74E-15	9.79E-14
CNAG_06766	1.128694722	1.39E-05	0.0001682967243
CNAG_01341	1.126538472	0.0002632089676	0.00218837298
CNAG_04259	1.125530367	7.11E-05	0.0007076530072
CNAG_00788	1.12416664	2.73E-11	9.08E-10
CNAG_02144	1.12394653	3.58E-14	1.76E-12
CNAG_04801	1.123546852	0.0001892723121	0.001641965642
CNAG_06147	1.122290846	0.0001504262436	0.001359937725
CNAG_05900	1.121766126	4.10E-07	6.67E-06
CNAG_06663	1.121174269	2.55E-06	3.62E-05
CNAG_03724	1.118355889	2.87E-06	4.04E-05
CNAG_04504	1.117574396	0.0002125645058	0.001800893729
CNAG_12827	1.115241613	0.00042680677	0.003250125141
CNAG_00703	1.113880256	6.08E-11	1.92E-09
CNAG_00626	1.113003301	4.19E-08	8.71E-07
CNAG_02385	1.112719407	0.009108988897	0.04222927252

CNAG_03196	1.111303238	0.001865267299	0.01130829709
CNAG_04676	1.1109337	2.97E-07	5.05E-06
CNAG_01055	1.108155704	6.24E-10	1.70E-08
CNAG_04395	1.107751882	0.003751521842	0.02043239575
CNAG_00086	1.106796704	0.002339193619	0.01373417125
CNAG_05360	1.106626077	0.001320531306	0.008494660901
CNAG_03782	1.104123599	0.006843532087	0.03366576269
CNAG_03525	1.103347068	0.004765568064	0.02487969994
CNAG_01432	1.10294123	0.001088198977	0.007265107223
CNAG_00686	1.102830241	1.43E-08	3.19E-07
CNAG_00684	1.099929188	2.14E-05	0.0002489362477
CNAG_03780	1.098129467	2.06E-10	6.10E-09
CNAG_03260	1.096767626	0.008538183258	0.03994975318
CNAG_01686	1.09620454	5.32E-05	0.0005501477296
CNAG_04192	1.095760188	2.52E-06	3.58E-05
CNAG_02421	1.095500647	0.00435097759	0.02311082964
CNAG_01397	1.091369699	8.13E-06	0.0001031521218
CNAG_01877	1.090591996	4.05E-08	8.44E-07
CNAG_07855	1.090530184	1.74E-06	2.56E-05
CNAG_03762	1.088360274	3.29E-06	4.57E-05
CNAG_02230	1.087493315	1.79E-07	3.21E-06
CNAG_07609	1.084751811	0.004122769753	0.02216275577
CNAG_12573	1.080237128	0.008478493263	0.03981593879
CNAG_03405	1.079628552	0.001042233563	0.006990443861

CNAG_05623	1.07409265	0.0002049343896	0.001749752903
CNAG_06866	1.074014291	4.89E-05	0.0005129499075
CNAG_00143	1.072395079	0.009669610471	0.04454323743
CNAG_05134	1.072280994	0.0001092835794	0.001019804095
CNAG_06277	1.065928259	1.05E-06	1.60E-05
CNAG_06246	1.063170464	3.38E-12	1.33E-10
CNAG_03283	1.061194798	1.05E-08	2.39E-07
CNAG_03951	1.061032101	0.001956287252	0.01180904648
CNAG_00706	1.060697887	0.002386810451	0.0139571812
CNAG_06946	1.060438591	3.98E-05	0.0004351966693
CNAG_06421	1.06037704	0.0005381579512	0.003983261769
CNAG_01744	1.059308927	1.29E-07	2.38E-06
CNAG_02502	1.059132848	2.97E-06	4.17E-05
CNAG_01044	1.057012032	0.0008864231694	0.006050438477
CNAG_04770	1.056101927	0.0008295733281	0.005771161388
CNAG_00106	1.056006594	0.002042026459	0.01227545989
CNAG_06123	1.054206981	2.64E-05	0.0003011232916
CNAG_06340	1.054195208	6.06E-05	0.0006183511012
CNAG_06840	1.053888873	7.96E-21	8.71E-19
CNAG_05480	1.053864097	2.11E-09	5.42E-08
CNAG_01238	1.053845791	0.000506801972	0.003779816509
CNAG_02128	1.053587613	4.76E-07	7.62E-06
CNAG_01404	1.053313373	5.23E-07	8.30E-06
CNAG_06151	1.051943924	0.003450447392	0.01909766543

CNAG_02507	1.05127345	2.51E-05	0.0002864212842
CNAG_07839	1.050867949	9.84E-13	4.10E-11
CNAG_01146	1.049004406	0.002322125497	0.01366164188
CNAG_02209	1.046913276	4.63E-08	9.58E-07
CNAG_03205	1.045947212	0.0045391586	0.02393487178
CNAG_04372	1.044221419	0.004047454147	0.02181860166
CNAG_01250	1.04405118	0.0006619535722	0.004783068518
CNAG_07884	1.043297603	1.48E-05	0.0001774547916
CNAG_03485	1.041086325	0.00169265429	0.01046844355
CNAG_03226	1.039543442	5.57E-09	1.33E-07
CNAG_05761	1.039267103	0.005898203057	0.02956754906
CNAG_04683	1.038697384	0.004897696679	0.02543203607
CNAG_03876	1.038013163	4.93E-06	6.54E-05
CNAG_03936	1.037630298	9.57E-05	0.0009150805289
CNAG_04589	1.037579265	0.007731360479	0.03705809262
CNAG_03341	1.037371347	0.003415784695	0.01897840106
CNAG_03595	1.036949553	0.006376009568	0.03167770274
CNAG_06092	1.035755013	9.00E-07	1.39E-05
CNAG_03861	1.03528452	1.14E-07	2.12E-06
CNAG_04147	1.035094536	0.0004734183271	0.003551930594
CNAG_06646	1.03457017	6.26E-09	1.48E-07
CNAG_00799	1.03278783	5.81E-08	1.14E-06
CNAG_02182	1.031302266	3.56E-08	7.55E-07
CNAG_05179	1.027957868	1.02E-07	1.93E-06

CNAG_00162	1.025006885	3.53E-11	1.15E-09
CNAG_03435	1.024627065	1.55E-08	3.44E-07
CNAG_04844	1.02462424	1.76E-06	2.58E-05
CNAG_05365	1.018969186	8.30E-05	0.0008043737442
CNAG_00666	1.016311449	0.0003007989203	0.002434538748
CNAG_05228	1.015825562	0.008657516743	0.04042732435
CNAG_01772	1.015677782	0.006238012975	0.03103838962
CNAG_06113	1.013250502	3.07E-10	8.71E-09
CNAG_01750	1.012594817	0.003413882185	0.01897840106
CNAG_05556	1.008739807	5.64E-08	1.12E-06
CNAG_04475	1.005456129	0.001474305555	0.009306754566
CNAG_01400	1.005134163	1.58E-07	2.87E-06
CNAG_05462	1.005122365	5.95E-11	1.89E-09
CNAG_06300	1.002799857	0.001147260591	0.007615549971
CNAG_02751	0.998333453	2.58E-10	7.53E-09
CNAG_04772	0.9976743754	0.003212953394	0.0180242642
CNAG_04380	-0.997706859	0.0007232152045	0.005142370687
CNAG_00583	-0.9990798241	0.0005750965539	0.004218588012
CNAG_02671	-1.000347415	4.77E-06	6.35E-05
CNAG_03242	-1.000974509	0.01036388361	0.04684766421
CNAG_02944	-1.003174386	0.00180479299	0.01102083812
CNAG_04307	-1.00677504	2.47E-09	6.29E-08
CNAG_02409	-1.007140704	0.0102304212	0.04635284666
CNAG_02581	-1.012611084	0.0006754149811	0.004849296743

CNAG_02114	-1.019998353	5.86E-06	7.64E-05
CNAG_00565	-1.020630628	1.87E-07	3.34E-06
CNAG_01169	-1.024469989	0.01081204581	0.04838286136
CNAG_02913	-1.025372922	0.004173816351	0.02239561644
CNAG_01946	-1.027031728	9.03E-08	1.72E-06
CNAG_04611	-1.03444644	7.14E-05	0.0007096240542
CNAG_02924	-1.034806618	1.44E-08	3.22E-07
CNAG_06983	-1.036006383	0.003241808723	0.01816855082
CNAG_07995	-1.040602399	4.59E-05	0.0004859362538
CNAG_06792	-1.041175131	0.007233226092	0.03531301197
CNAG_02704	-1.042292889	0.001225896933	0.008004229279
CNAG_02994	-1.044582891	6.85E-08	1.32E-06
CNAG_02929	-1.045352315	0.001385430615	0.008846187994
CNAG_01183	-1.048111583	0.005699218264	0.02866580143
CNAG_01602	-1.049327853	0.001792014225	0.01096591598
CNAG_03475	-1.049769506	0.0002718736457	0.002241120593
CNAG_02107	-1.050550174	0.0009215146801	0.006275179284
CNAG_03928	-1.050689362	0.003452653722	0.01909766543
CNAG_02915	-1.051349362	0.0002579459575	0.002150786797
CNAG_02921	-1.052956922	9.19E-14	4.26E-12
CNAG_00015	-1.053326335	0.00551529945	0.02799505192
CNAG_03316	-1.05382018	2.33E-05	0.0002694817567
CNAG_06804	-1.054931442	0.001277850751	0.008283160068
CNAG_05352	-1.055138417	7.34E-05	0.0007261434385

CNAG_06106	-1.057470373	0.007145897835	0.03494555102
CNAG_06834	-1.062114044	0.0003804526185	0.00295935963
CNAG_03075	-1.064740511	0.000705047244	0.005037914647
CNAG_03013	-1.06475513	0.0002024046244	0.001734013783
CNAG_02725	-1.068006512	0.0002299917313	0.0019344007
CNAG_01900	-1.072016178	0.005469644237	0.02782843578
CNAG_04417	-1.072396533	6.54E-12	2.42E-10
CNAG_03920	-1.073900266	4.12E-06	5.55E-05
CNAG_02366	-1.0753274	0.004393608015	0.023294564
CNAG_02848	-1.075384531	0.009936427562	0.04530416815
CNAG_04540	-1.075881332	0.002738899026	0.01568487678
CNAG_02886	-1.076396688	4.35E-05	0.0004672197191
CNAG_03672	-1.078022594	8.02E-05	0.0007841830774
CNAG_01539	-1.078499057	2.41E-19	2.25E-17
CNAG_06016	-1.081042751	0.001678278274	0.0104052938
CNAG_05218	-1.081767468	6.78E-07	1.06E-05
CNAG_02500	-1.086479694	1.22E-10	3.76E-09
CNAG_00648	-1.091328605	0.0002293873448	0.001932121603
CNAG_02748	-1.094686454	3.18E-16	1.96E-14
CNAG_02511	-1.095832329	0.003724937501	0.02034496967
CNAG_03018	-1.098018721	0.0002712459519	0.002239131469
CNAG_02794	-1.108197376	3.37E-06	4.64E-05
CNAG_02445	-1.108810974	0.000424463073	0.003241637077
CNAG_02826	-1.11129994	0.01113828791	0.04957479144

CNAG_04414	-1.113349925	0.00222493997	0.01312986469
CNAG_06452	-1.118177402	0.001092514047	0.00728552233
CNAG_00977	-1.118619083	0.003035350142	0.01717759187
CNAG_08002	-1.118977205	0.009705230086	0.04463635583
CNAG_00249	-1.119336708	0.0009905271675	0.006697905409
CNAG_01982	-1.12005751	0.001006355506	0.006789092148
CNAG_04689	-1.121341116	0.004309562254	0.0229750812
CNAG_06500	-1.12451294	0.004760452061	0.02487540099
CNAG_02868	-1.124513678	5.12E-07	8.15E-06
CNAG_07532	-1.12480027	0.003688798181	0.02018563311
CNAG_06887	-1.126982515	0.008986304828	0.04182666951
CNAG_02846	-1.129894857	0.009228467535	0.04274897631
CNAG_02676	-1.133585191	0.0004245733462	0.003241637077
CNAG_03049	-1.133611543	6.16E-06	8.01E-05
CNAG_04017	-1.134116844	5.27E-08	1.06E-06
CNAG_03262	-1.134875901	0.001019978953	0.006865015134
CNAG_02796	-1.139824074	0.0008013814385	0.005608702217
CNAG_01796	-1.141366923	5.70E-07	9.00E-06
CNAG_04521	-1.145516077	0.01068651574	0.04793216619
CNAG_03348	-1.145963899	0.004425545506	0.02342103763
CNAG_02845	-1.146365206	3.85E-05	0.0004246505054
CNAG_00436	-1.155488444	0.005278544049	0.0269508042
CNAG_05005	-1.156838336	0.001342752301	0.008617109173
CNAG_07530	-1.164438233	4.52E-05	0.0004824474975

CNAG_02593	-1.164615151	0.0003400850248	0.002693045203
CNAG_05882	-1.16777962	0.0005087445615	0.003789427678
CNAG_03162	-1.169531039	0.000102710959	0.0009709788048
CNAG_04111	-1.171621753	0.002457239228	0.01432565526
CNAG_01331	-1.172345299	0.002742081852	0.01568644061
CNAG_02851	-1.173052236	0.001276765129	0.008283160068
CNAG_07539	-1.175757933	5.19E-05	0.0005409142716
CNAG_02095	-1.179236675	0.005425434833	0.02765206232
CNAG_02670	-1.180784056	0.003372669325	0.01881099012
CNAG_05308	-1.183022838	0.007130723572	0.03490079654
CNAG_00663	-1.189324712	5.25E-08	1.06E-06
CNAG_07514	-1.191202713	5.58E-05	0.0005756174354
CNAG_02801	-1.193663837	1.26E-15	7.16E-14
CNAG_02995	-1.197088903	8.45E-06	0.0001066458862
CNAG_02956	-1.199800589	4.08E-05	0.0004440869014
CNAG_07622	-1.200007742	0.006462839882	0.0320103907
CNAG_04658	-1.200749819	0.001577755747	0.009909293824
CNAG_02874	-1.206233579	0.004178620397	0.02240065236
CNAG_02823	-1.20634072	0.002966305855	0.01685268866
CNAG_02606	-1.208043752	0.01050640055	0.0473441611
CNAG_03958	-1.209311072	1.26E-05	0.0001551700615
CNAG_03011	-1.214044708	0.005041887387	0.02608726554
CNAG_02976	-1.215241959	0.003587071274	0.01972208542
CNAG_02883	-1.216022408	0.0006677116507	0.00480669443

CNAG_01108	-1.217332986	2.11E-05	0.0002466029176
CNAG_00287	-1.219070052	2.01E-05	0.0002361134388
CNAG_10501	-1.219299831	5.84E-12	2.20E-10
CNAG_04354	-1.222037197	0.0001939429738	0.001674962047
CNAG_01567	-1.22396673	0.00209797721	0.01253379168
CNAG_02264	-1.226197018	0.000464959165	0.003503821016
CNAG_03805	-1.227075433	7.40E-07	1.16E-05
CNAG_03763	-1.22710223	8.89E-05	0.0008559034297
CNAG_00374	-1.23784468	1.07E-08	2.43E-07
CNAG_02895	-1.241247166	1.89E-06	2.74E-05
CNAG_02983	-1.244038135	6.74E-05	0.0006781683275
CNAG_02761	-1.245321082	9.66E-13	4.06E-11
CNAG_02284	-1.247635793	0.01033289008	0.04675268251
CNAG_02104	-1.257699154	0.0008580238324	0.005898277709
CNAG_01466	-1.258022599	0.004134054952	0.02220282525
CNAG_03050	-1.258078498	0.004506807618	0.0238076118
CNAG_07029	-1.25955871	1.08E-08	2.45E-07
CNAG_03020	-1.261731735	0.001012254754	0.006820949182
CNAG_00519	-1.264887731	9.61E-05	0.0009173586818
CNAG_06443	-1.266187666	1.13E-21	1.52E-19
CNAG_03025	-1.266559688	0.0001906187772	0.001650392985
CNAG_02700	-1.271150693	6.90E-05	0.0006918086331
CNAG_05193	-1.271522189	0.0001908133391	0.001650392985
CNAG_05741	-1.271586723	0.0001983866143	0.001705712804

CNAG_02911	-1.278711783	0.004605120195	0.02419462514
CNAG_02766	-1.279518284	0.001124707036	0.007474400542
CNAG_00854	-1.279914897	0.00177059873	0.01085779856
CNAG_07556	-1.284830912	0.001350417403	0.008647147903
CNAG_04371	-1.287339342	0.004860567288	0.02533002467
CNAG_06121	-1.289295511	2.22E-07	3.89E-06
CNAG_05575	-1.293375283	0.005048068359	0.0260959466
CNAG_02937	-1.299039024	3.55E-06	4.87E-05
CNAG_01739	-1.30011204	0.002156139546	0.01280207855
CNAG_06021	-1.301478557	1.70E-06	2.52E-05
CNAG_02964	-1.306606529	0.0005120482135	0.003809139149
CNAG_01261	-1.309025945	5.19E-12	1.98E-10
CNAG_01874	-1.311485969	0.0007768919319	0.005470338694
CNAG_02757	-1.315441469	0.0001366280588	0.001246865513
CNAG_03609	-1.316816299	0.001165938708	0.00771303061
CNAG_02223	-1.320766354	1.32E-05	0.0001608714891
CNAG_04432	-1.325034311	0.004747964557	0.02483254026
CNAG_03045	-1.327558569	1.28E-07	2.37E-06
CNAG_03638	-1.333897906	1.51E-10	4.55E-09
CNAG_00769	-1.338126518	6.64E-06	8.54E-05
CNAG_02990	-1.338294884	1.29E-16	8.54E-15
CNAG_06722	-1.340566202	0.0001524756098	0.001375041465
CNAG_02830	-1.343184825	3.10E-06	4.32E-05
CNAG_06926	-1.343453959	0.006773034504	0.03337562496

CNAG_02605	-1.344079428	0.001054149108	0.007062189686
CNAG_05852	-1.345635197	3.34E-06	4.62E-05
CNAG_06008	-1.347830785	0.008221516852	0.03892458346
CNAG_05469	-1.347998376	0.0009695462208	0.006586776494
CNAG_05113	-1.35618446	0.0001070021035	0.001003438722
CNAG_02501	-1.359537204	0.007277504814	0.03546942001
CNAG_02843	-1.360212169	2.60E-24	4.85E-22
CNAG_03017	-1.362013345	9.65E-05	0.000919566441
CNAG_02434	-1.364217147	0.0004737950559	0.003551930594
CNAG_06839	-1.364643318	9.23E-06	0.0001157254283
CNAG_07564	-1.366208048	0.0008791499878	0.006014963612
CNAG_07506	-1.367468026	1.17E-09	3.14E-08
CNAG_02854	-1.370921504	0.0003299951524	0.00263768539
CNAG_00727	-1.376821215	0.0003062778717	0.002471978087
CNAG_02788	-1.381170822	0.0006067042122	0.004422453974
CNAG_07320	-1.381434773	0.005603964464	0.02836242277
CNAG_03463	-1.382611983	1.08E-06	1.64E-05
CNAG_02770	-1.38330168	0.001748137989	0.01075420344
CNAG_07529	-1.384284819	1.86E-05	0.0002191533817
CNAG_07579	-1.386344026	0.0009806773979	0.006639048506
CNAG_00691	-1.386863334	4.39E-05	0.0004702549133
CNAG_02897	-1.396094095	0.0002995890525	0.002431984614
CNAG_04025	-1.396135458	0.003133738712	0.01766538505
CNAG_03786	-1.400494681	0.0004016451282	0.003099245696

CNAG_07540	-1.402106438	3.52E-11	1.15E-09
CNAG_02950	-1.402548361	4.61E-07	7.44E-06
CNAG_02772	-1.402734734	0.002299814354	0.01354413026
CNAG_05807	-1.411698422	0.0008520405485	0.005878065451
CNAG_07512	-1.412598313	3.52E-07	5.85E-06
CNAG_00306	-1.414013587	4.47E-13	1.92E-11
CNAG_04544	-1.421402239	0.009778527631	0.04488074331
CNAG_02827	-1.422473781	1.58E-06	2.35E-05
CNAG_00515	-1.43025216	0.0006490972145	0.004696027912
CNAG_02010	-1.431560801	4.66E-08	9.59E-07
CNAG_03808	-1.431897091	1.80E-06	2.63E-05
CNAG_01375	-1.438092241	4.99E-09	1.20E-07
CNAG_07566	-1.439054312	0.007689040542	0.03694302998
CNAG_07365	-1.440910937	0.002155424555	0.01280207855
CNAG_02523	-1.44221874	0.001798244142	0.01099243123
CNAG_04600	-1.445842932	5.22E-06	6.89E-05
CNAG_01542	-1.448873173	4.62E-27	1.07E-24
CNAG_04538	-1.457394959	0.002349970631	0.01378348158
CNAG_07943	-1.460063946	0.0001020897978	0.000968265758
CNAG_06658	-1.462723921	0.0002084411984	0.001773739713
CNAG_06874	-1.463045884	0.00173187729	0.01067683925
CNAG_02600	-1.463623204	0.001653176573	0.01029842096
CNAG_07972	-1.46962334	0.0003563821522	0.002798420829
CNAG_01314	-1.47084942	3.58E-10	1.01E-08

CNAG_02595	-1.475944346	0.01039937053	0.04697143587
CNAG_02935	-1.476291904	2.42E-09	6.18E-08
CNAG_03016	-1.47894039	2.65E-08	5.73E-07
CNAG_04606	-1.481164993	0.003210044591	0.0180242642
CNAG_03913	-1.489059302	0.0004967577539	0.003714466044
CNAG_07517	-1.490975774	2.07E-05	0.0002419314473
CNAG_03071	-1.494362561	0.004317947788	0.02299862815
CNAG_05109	-1.495653007	1.57E-10	4.70E-09
CNAG_05715	-1.499963949	5.37E-05	0.0005549879266
CNAG_04804	-1.509497902	3.13E-08	6.72E-07
CNAG_01519	-1.5158276	0.002120897314	0.01263165461
CNAG_02817	-1.518705071	1.62E-21	2.04E-19
CNAG_06331	-1.521918384	0.00324778259	0.01818444455
CNAG_00816	-1.52504993	5.57E-08	1.11E-06
CNAG_02569	-1.530492634	0.0005518342066	0.004063378942
CNAG_06891	-1.542395468	0.0001559916468	0.001392868402
CNAG_02855	-1.545158399	1.53E-09	4.02E-08
CNAG_02675	-1.549347199	0.0003146537807	0.002525510608
CNAG_02662	-1.549472504	1.94E-05	0.000228041645
CNAG_02078	-1.554145434	3.04E-08	6.55E-07
CNAG_05824	-1.558830419	0.005170464352	0.02660012498
CNAG_06017	-1.56250449	0.003761850982	0.0204694145
CNAG_06863	-1.565745243	1.33E-05	0.0001623169465
CNAG_06928	-1.573174716	0.0004162716518	0.003190865373

CNAG_03034	-1.574949876	0.003060144945	0.01730101459
CNAG_05607	-1.575796503	0.003340031163	0.01864689845
CNAG_04861	-1.578746557	0.002597583314	0.01506806337
CNAG_01252	-1.58255729	0.002487180653	0.01447109627
CNAG_01545	-1.588587636	0.008527250991	0.03994779264
CNAG_01872	-1.591368095	0.00969579556	0.04462838385
CNAG_00639	-1.592712874	1.84E-07	3.29E-06
CNAG_03058	-1.596880871	1.58E-43	7.64E-41
CNAG_03004	-1.597432307	0.0002979211791	0.002424793866
CNAG_02985	-1.600278876	1.17E-11	4.13E-10
CNAG_02137	-1.605522822	2.71E-06	3.82E-05
CNAG_06433	-1.615209391	0.007615563652	0.03671563341
CNAG_02993	-1.62522462	0.0001817809526	0.001586476838
CNAG_03083	-1.629773285	0.00523164357	0.02682953495
CNAG_01272	-1.632010484	4.85E-11	1.56E-09
CNAG_01129	-1.632089108	6.34E-05	0.0006423465711
CNAG_06376	-1.634838925	0.001220193783	0.007980838571
CNAG_02217	-1.634944395	0.0001089564452	0.00101839129
CNAG_02723	-1.63640396	5.66E-05	0.0005815985185
CNAG_00790	-1.643843607	7.90E-06	0.0001006221344
CNAG_06934	-1.659346551	4.86E-06	6.46E-05
CNAG_02755	-1.660180884	0.000299644006	0.002431984614
CNAG_02862	-1.663340585	0.003633867606	0.01994153672
CNAG_07337	-1.673074479	0.0003329552163	0.002654024042

CNAG_02980	-1.675594859	0.0001680798632	0.001484598355
CNAG_07108	-1.685108624	2.40E-07	4.16E-06
CNAG_06460	-1.691976183	8.84E-22	1.22E-19
CNAG_06371	-1.692188098	5.27E-05	0.0005460730939
CNAG_03036	-1.695533773	0.0006130108987	0.00446125407
CNAG_02941	-1.698561991	4.50E-10	1.25E-08
CNAG_05632	-1.70928736	0.007890975915	0.03766738503
CNAG_02873	-1.716990896	0.0001528087496	0.001375041465
CNAG_02878	-1.719174445	0.0002226282654	0.00188065714
CNAG_03176	-1.727783926	1.13E-15	6.60E-14
CNAG_07559	-1.728251137	4.60E-24	8.32E-22
CNAG_02927	-1.730215411	1.98E-10	5.88E-09
CNAG_02667	-1.733892779	0.002165492199	0.01283112054
CNAG_01343	-1.738174453	0.005585154143	0.0282919303
CNAG_07580	-1.741392422	9.48E-06	0.0001183534547
CNAG_02255	-1.744665911	0.0002265068792	0.00191063663
CNAG_06136	-1.748619994	0.006804984914	0.03350457738
CNAG_02841	-1.752932429	2.17E-08	4.72E-07
CNAG_02678	-1.753918795	0.0004047946955	0.003113807667
CNAG_02728	-1.75401042	0.0001070103762	0.001003438722
CNAG_02702	-1.760399545	8.30E-12	3.00E-10
CNAG_02665	-1.762823786	9.00E-07	1.39E-05
CNAG_06884	-1.763899952	0.0003119025432	0.002506900469
CNAG_02774	-1.772553866	1.39E-10	4.25E-09

CNAG_04406	-1.774450822	4.28E-05	0.0004614821291
CNAG_02999	-1.775355563	9.91E-05	0.0009417006355
CNAG_02747	-1.775990218	3.46E-05	0.0003859741413
CNAG_06009	-1.778784094	6.12E-12	2.29E-10
CNAG_03024	-1.781748215	1.98E-05	0.0002327125466
CNAG_02989	-1.792145144	0.0003854858409	0.002986484556
CNAG_02903	-1.809863014	3.72E-14	1.80E-12
CNAG_03909	-1.821899192	3.91E-06	5.31E-05
CNAG_02834	-1.821912402	1.42E-05	0.0001713776233
CNAG_05740	-1.822294907	0.01071282127	0.04801299247
CNAG_02008	-1.830288219	5.73E-27	1.28E-24
CNAG_07541	-1.831411374	7.12E-11	2.22E-09
CNAG_01737	-1.831544052	4.87E-22	7.06E-20
CNAG_02975	-1.836969783	1.09E-07	2.05E-06
CNAG_02940	-1.840409125	1.75E-07	3.14E-06
CNAG_01424	-1.841419734	2.46E-07	4.25E-06
CNAG_01081	-1.84709575	4.32E-05	0.000464199682
CNAG_07510	-1.851135348	1.52E-14	7.84E-13
CNAG_05965	-1.865313555	0.004968428068	0.02577622261
CNAG_03234	-1.868166208	0.002433662663	0.01420249258
CNAG_03079	-1.880745952	0.0005451122532	0.004018989195
CNAG_00023	-1.88226436	6.17E-05	0.0006286971466
CNAG_05124	-1.88901558	0.003086424974	0.0174325855
CNAG_12417	-1.894927712	0.004679782423	0.02452019814

CNAG_03366	-1.896195583	7.05E-05	0.0007041288974
CNAG_02779	-1.896880107	3.64E-14	1.77E-12
CNAG_02789	-1.906609515	4.94E-12	1.90E-10
CNAG_04280	-1.908928472	3.41E-07	5.68E-06
CNAG_06285	-1.912684879	2.58E-07	4.43E-06
CNAG_02696	-1.944560969	2.21E-05	0.0002562382259
CNAG_02968	-1.948600353	0.002113172517	0.01259859541
CNAG_02771	-1.991618043	1.45E-09	3.83E-08
CNAG_07474	-1.993311926	0.005935357528	0.02972808719
CNAG_02603	-1.997228857	0.009807230189	0.04496273651
CNAG_02979	-2.012692997	1.11E-06	1.67E-05
CNAG_06590	-2.020500234	5.42E-06	7.14E-05
CNAG_07498	-2.023262476	3.81E-15	2.08E-13
CNAG_05032	-2.026279495	3.13E-07	5.29E-06
CNAG_06903	-2.032124457	1.09E-06	1.65E-05
CNAG_05415	-2.053388525	0.009894906779	0.04522159683
CNAG_12333	-2.053703751	0.001828535382	0.01114233705
CNAG_00498	-2.057623332	0.001461237018	0.009244397946
CNAG_02680	-2.058845654	8.17E-05	0.0007926025318
CNAG_02693	-2.06945088	9.38E-06	0.0001174030119
CNAG_02661	-2.082827858	3.09E-17	2.21E-15
CNAG_07344	-2.09348358	0.0001891684805	0.001641965642
CNAG_03381	-2.103819271	0.01004052478	0.04574279962
CNAG_02998	-2.126129105	7.17E-07	1.12E-05

CNAG_06239	-2.126656868	0.0006991370137	0.005001850611
CNAG_02787	-2.135210073	3.96E-07	6.49E-06
CNAG_03056	-2.141417249	0.002658308411	0.01532884259
CNAG_02592	-2.161370875	0.00488594584	0.02539377232
CNAG_02866	-2.170198053	3.42E-08	7.29E-07
CNAG_00692	-2.170337961	0.006850535011	0.03367162883
CNAG_02932	-2.172490356	0.006698544008	0.0330647892
CNAG_04735	-2.196257341	1.73E-51	1.18E-48
CNAG_02955	-2.197511232	0.0001714858308	0.001507982382
CNAG_07555	-2.204650017	5.87E-11	1.88E-09
CNAG_01420	-2.212479015	9.91E-08	1.88E-06
CNAG_06149	-2.213284558	3.89E-06	5.31E-05
CNAG_02004	-2.219256105	1.02E-32	3.11E-30
CNAG_03067	-2.220521731	0.0001563816391	0.001394202459
CNAG_02881	-2.229559928	0.00739866271	0.03593901962
CNAG_02934	-2.242348946	1.98E-16	1.26E-14
CNAG_01821	-2.264202283	9.31E-17	6.28E-15
CNAG_02735	-2.268062685	6.92E-09	1.62E-07
CNAG_02715	-2.274641781	4.17E-05	0.0004521417173
CNAG_02344	-2.278396497	0.0001729812106	0.001518827448
CNAG_04117	-2.280257021	0.001970499832	0.01187011073
CNAG_05304	-2.285041205	0.0004678124198	0.00352074412
CNAG_02818	-2.285911854	7.34E-35	2.50E-32
CNAG_01795	-2.286373837	9.42E-10	2.55E-08

CNAG_02655	-2.288172133	4.84E-10	1.34E-08
CNAG_02716	-2.292668578	3.78E-11	1.22E-09
CNAG_12243	-2.293534973	0.008746805276	0.04077854914
CNAG_02764	-2.303692609	0.000429088182	0.003258933178
CNAG_02741	-2.33024239	8.48E-11	2.63E-09
CNAG_06901	-2.331797542	0.0001240850239	0.001145020245
CNAG_02888	-2.333779341	0.0004054372352	0.003113807667
CNAG_04858	-2.339382159	0.01060295665	0.04770507283
CNAG_02602	-2.345910068	2.00E-06	2.88E-05
CNAG_04736	-2.347030643	0.0003003611408	0.00243439554
CNAG_02585	-2.349191255	7.26E-46	3.83E-43
CNAG_06973	-2.381842402	0.01022840973	0.04635284666
CNAG_07538	-2.38881604	1.76E-49	1.02E-46
CNAG_03059	-2.394436292	4.73E-09	1.15E-07
CNAG_02918	-2.39933969	1.66E-41	6.88E-39
CNAG_02681	-2.412208288	2.04E-11	6.93E-10
CNAG_13083	-2.425273145	1.40E-06	2.11E-05
CNAG_02861	-2.425987225	0.0008452124578	0.005851859251
CNAG_04874	-2.43416724	4.99E-33	1.61E-30
CNAG_06913	-2.450282539	4.87E-21	5.76E-19
CNAG_03039	-2.458366879	2.60E-14	1.30E-12
CNAG_02415	-2.471493686	3.59E-19	3.20E-17
CNAG_03539	-2.476730033	0.00568662582	0.02863075293
CNAG_00596	-2.491124102	1.00E-05	0.000124398034

CNAG_02819	-2.496173475	1.60E-08	3.53E-07
CNAG_12593	-2.519975858	0.004973687293	0.02578034955
CNAG_01803	-2.551199703	3.52E-28	8.87E-26
CNAG_07520	-2.551491242	2.25E-07	3.91E-06
CNAG_02900	-2.611684584	6.85E-06	8.78E-05
CNAG_06892	-2.618077641	5.27E-21	6.11E-19
CNAG_03019	-2.61872212	8.60E-14	4.08E-12
CNAG_02904	-2.654863604	0.0001802047491	0.001575092792
CNAG_02701	-2.666108194	0.00121461122	0.007958162493
CNAG_07316	-2.676719336	0.002283385	0.01346105399
CNAG_06507	-2.680502663	3.97E-05	0.0004351966693
CNAG_02576	-2.690391911	5.10E-07	8.14E-06
CNAG_03007	-2.703352416	5.43E-55	4.49E-52
CNAG_01121	-2.79628666	3.81E-07	6.29E-06
CNAG_01242	-2.86439065	2.80E-12	1.11E-10
CNAG_00586	-2.873240856	6.58E-13	2.80E-11
CNAG_12570	-2.873852036	1.28E-07	2.37E-06
CNAG_01369	-2.875502933	0.002472197889	0.01439837866
CNAG_12217	-2.909572967	7.44E-24	1.31E-21
CNAG_01506	-3.099411603	7.82E-07	1.22E-05
CNAG_03054	-3.197857731	0.002739101864	0.01568487678
CNAG_02599	-3.285587656	0.009760338855	0.04485421385
CNAG_03047	-3.359947867	8.39E-12	3.02E-10
CNAG_05654	-3.392595104	1.21E-39	4.69E-37

CNAG_04139	-4.305495499	0.002179985621	0.01287769283
CNAG_03076	-4.532304364	0.001255428475	0.008157174312
CNAG_04891	-4.896174451	2.45E-83	7.09E-80
