# Supplementary material for: Integrative Transcriptomic and Systems Biology Analyses Identify TCB1 as a Calcium-Responsive Gene in Cryptococcus neoformans
Source: Microorganisms. 2026 Jan 7;14(1):122. doi: 10.3390/microorganisms14010122 (PMC12843964; doi:10.3390/microorganisms14010122)
Supplement: Supplementary file 1 [file microorganisms-14-00122-s001.zip › Supplementary Table S6.pdf]

Supplementary Table S6. Cross-referencing Dynet PPIN overlaps with FungiDB annotations and conserved domains

Nodes present in Pmc1, Cna1 and Crz1 PPINs		
**Gene ID (*C. neoformans* JEC21)**	**FungiDB product description**	**Interpro description according to FungiDB**
CNK01510	Identified spore protein 4	Glycoside hydrolase superfamily;Uncharacterised protein family, glycosyl hydrolase catalytic domain
CNG02620	rho gdp-dissociation inhibitor	Rho protein GDP-dissociation inhibitor;Immunoglobulin E-set
CNJ03160	conserved hypothetical protein	Ricin B, lectin domain;Ricin B-like lectins
CNF03630	conserved hypothetical protein	Peptidase S54, rhomboid;Peptidase S54, rhomboid domain
CNC06750	conserved hypothetical protein	Bestrophin/UPF0187
CNA06700	conserved hypothetical protein	FAD dependent oxidoreductase;FAD/NAD(P)-binding domain superfamily
CNB02770	expressed protein	N/A
CNC00410	hypothetical protein	Organic solute transporter subunit alpha/Transmembrane protein 184
CNA05700	expressed protein	N/A
CNA06690	hypothetical protein	N/A
CNJ03020	conserved hypothetical protein	Glycoside hydrolase family 16;Concanavalin A-like lectin/glucanase domain superfamily
CNH02735	hypothetical protein	Cation-independent mannose-6-phosphate receptor repeat
CND02090	expressed protein	NAD(P)-binding domain superfamily
CNJ01670	conserved hypothetical protein	Sulfatase-modifying factor enzyme;C-type lectin fold;Histidine-specific methyltransferase, SAM-

		dependent;DinB-like domain
CNB01890	intracellular protein transport-related protein, putative	Clathrin adaptor, mu subunit;Longin-like domain superfamily;Clathrin adaptor, mu subunit, conserved site;AP complex, mu/sigma subunit;Mu homology domain;AP-2 complex subunit mu, C-terminal superfamily;Mu2, C-terminal domain;AP-2 complex subunit mu, N-terminal
CNC00660	long-chain-fatty-acid-CoA ligase, putative	AMP-dependent synthetase/ligase;AMP-binding, conserved site
CNC03090	expressed protein	Protein of unknown function DUF4112
CND03920	hypothetical protein	N/A
CNE01410	conserved hypothetical protein	Annexin;Annexin repeat, conserved site;Annexin repeat;Annexin superfamily
CNE04230	hypothetical protein	Membrane protein SUR7/Rim9-like, fungi
CNH02920	conserved hypothetical protein	Membrane protein SUR7/Rim9-like, fungi
CNG01260	expressed protein	Eisosome component PIL1/LSP1
CNN01210	hypothetical protein	N/A
CND03510	putative calcium-transporting ATPase	P-type ATPase;Cation-transporting P-type ATPase, N-terminal;Cation-transporting P-type ATPase, C-terminal;P-type ATPase, subfamily IIB;P-type ATPase, A domain superfamily;P-type ATPase, phosphorylation site;P-type ATPase, transmembrane domain superfamily;P-type ATPase, cytoplasmic domain N;HAD-like superfamily
CNC06180	conserved hypothetical protein	Protein of unknown function DUF3759
CNB00540	conserved hypothetical protein	WD40 repeat;BING4, C-terminal domain;WD40 repeat, conserved site;WD40-repeat-containing domain superfamily;WD repeat-containing protein WDR46/Utp7

CNM01510	expressed protein	N/A
**Nodes present in Pmc1 and Cna1 PPINs**		
**Gene ID (*C. neoformans* JEC21)**	**FungiDB product description**	**Interpro description according to FungiDB**
CNN02120	conserved hypothetical protein	Store-operated calcium entry-associated regulatory factor
CNN00370	conserved hypothetical protein	NTF2-like domain superfamily;SnoaL-like domain
CNJ00940	expressed protein	N/A
CNK00880	conserved hypothetical protein	Oxoglutarate/iron-dependent dioxygenase;Non-haem dioxygenase N-terminal domain
CNG03085	hypothetical protein	Proteolipid membrane potential modulator
CNM02550	conserved hypothetical protein	Purine-cytosine permease
CNN01300	hypothetical protein	N/A
CNK01810	hypothetical protein	Mitochondrial substrate/solute carrier;Mitochondrial carrier domain superfamily
CND01860	phospholipid transporter, putative	Major facilitator, sugar transporter-like;MFS transporter superfamily
CNC01350	expressed protein	N/A
CND03690	thiosulfate sulfurtransferase, putative	Rhodanese-like domain;Rhodanese-like domain superfamily
CNB05260	conserved hypothetical protein	Glutathione-dependent formaldehyde-activating enzyme/centromere protein V;Mss4-like superfamily
CNB02870	mitochondrial 40s ribosomal protein, putative	Ribosomal protein S14;Ribosomal protein S14, conserved site
CNA07570	large subunit ribosomal protein L27e	Ribosomal protein L27e;Translation protein SH3-like domain superfamily;Eukaryotic Ribosomal Protein L27, KOW domain
CNB00020	expressed protein	N/A
CNI03450	expressed protein	Glutathione-dependent formaldehyde-activating enzyme/centromere protein V;Mss4-like superfamily
CNH03660	alpha-amylase AmyA, putative	Glycosyl hydrolase, family 13, catalytic domain;Alpha-amylase, domain of unknown function DUF1966, C-terminal;Glycoside hydrolase superfamily

CNK00270	UDP-N-acetylglucosamine-dolichyl-phosphate N-acetylglucosaminephosphotransferase, putative	Glycosyl transferase, family 4;UDP-GlcNAc-dolichyl-phosphate GlcNAc phosphotransferase
CNI01310	transporter, putative	Tetracycline resistance protein/drug resistance transporter;Major facilitator superfamily;MFS transporter superfamily
CNA06780	transmembrane receptor, putative	Carbohydrate-binding WSC;Domain of unknown function DUF1996
CNJ02910	glutamate synthase (NADH), putative	Glutamate synthase, alpha subunit, C-terminal;Glutamate synthase domain;Glutamate synthase, NADH/NADPH, small subunit 1;Glutamate synthase, central-N;Alpha-helical ferredoxin;Glutamate synthase, eukaryotic;Glutamine amidotransferase type 2 domain;FAD/NAD(P)-binding domain;Dihydroprymidine dehydrogenase domain II;Nucleophile aminohydrolases, N-terminal;Glutamate synthase, alpha subunit, C-terminal domain superfamily
CNB02680	high-affinity glucose transporter	Sugar/inositol transporter;Major facilitator, sugar transporter-like;Sugar transporter, conserved site;MFS transporter superfamily
CNH02990	myo-inositol transporter	Sugar/inositol transporter;Major facilitator, sugar transporter-like;Sugar transporter, conserved site;MFS transporter superfamily
CNH00540	galactose transporter, putative	Sugar/inositol transporter;Major facilitator, sugar transporter-like;Sugar transporter, conserved site;MFS transporter superfamily
CNB00220	fructosyl amino acid oxidase, putative	FAD dependent oxidoreductase;FAD/NAD(P)-binding domain superfamily
CNC02930	expressed protein	N/A
CNJ02310	sterol regulatory element-binding protein	Myc-type, basic helix-loop-helix (bHLH) domain;Helix-loop-helix DNA-binding domain superfamily
CNK00920	hypothetical protein	N/A
CNC05950	phosphoribosyl-ATP diphosphatase, putative	NUDIX hydrolase domain;NUDIX hydrolase-like domain superfamily;NUDIX hydrolase, conserved site
CNA05170	structural constituent of ribosome, putative	Ribosomal protein L16;Ribosomal protein L10e/L16;Ribosomal protein L16, conserved site;Ribosomal protein L10e/L16 superfamily
CNB01910	60s ribosomal protein l19, mitochondrial precursor, putative	Ribosomal protein L11/L12;Ribosomal protein L11, bacterial-type;Ribosomal protein L11, C-terminal;Ribosomal protein L11, N-terminal;Ribosomal protein L11, C-terminal domain superfamily;Ribosomal protein L11/L12, N-terminal

		domain superfamily
CNB04460	hypothetical protein	SH3-binding, glutamic acid-rich protein;Thioredoxin-like superfamily
CNA02390	hypothetical protein	N/A
CNL05050	conserved hypothetical protein	FAD dependent oxidoreductase;FAD/NAD(P)-binding domain superfamily
CNC00760	pr4/barwin domain protein	RlpA-like protein, double-psi beta-barrel domain;RlpA-like domain superfamily
CNC05410	hypothetical protein	DNA recombination and repair protein Rad51-like, C-terminal;P-loop containing nucleoside triphosphate hydrolase
CNA05010	C-5 sterol desaturase, putative	Fatty acid hydroxylase
CNC02410	C-4 methyl sterol oxidase, putative	Fatty acid hydroxylase
CNE03540	D-lactaldehyde dehydrogenase, putative	NAD-dependent epimerase/dehydratase;NAD(P)-binding domain superfamily
CNI02420	uricase (Urate oxidase), putative	Uricase
CNB03010	mitochondrial ribosomal protein L23, putative	Ribosomal protein L13;Ribosomal protein L13, bacterial-type;Ribosomal protein L13 superfamily
CND04600	ribosomal protein, putative	Ribosomal protein S2;Ribosomal protein S2, bacteria/mitochondria/plastid;Ribosomal protein S2, flavodoxin-like domain superfamily
CNE01390	asparagine-tRNA ligase, putative	Aspartyl/Asparaginyl-tRNA synthetase, class IIb;Aminoacyl-tRNA synthetase, class II (D/K/N);OB-fold nucleic acid binding domain, AA-tRNA synthetase-type;Asparagine-tRNA ligase;Nucleic acid-binding, OB-fold
CNE03920	60s ribosomal protein l1-a (l10a), putative	Ribosomal protein L1;Ribosomal protein L1, conserved site;Ribosomal protein L1-like;Ribosomal protein L1/ribosomal biogenesis protein
CNG03160	ribosomal protein L6, putative	Ribosomal protein L6;Ribosomal protein L6, bacterial-type;Ribosomal protein L6, alpha-beta domain;Ribosomal protein L6, alpha-beta domain superfamily
CNI03170	conserved hypothetical protein	Ribosomal protein L15, bacterial-type;Ribosomal protein L18e/L15P;Ribosomal protein L15;Ribosomal L18e/L15P superfamily
CNA05040	conserved hypothetical protein	C2 domain;Tricalbin, C2B domain;Mug190-like, C2A domain
CNB04170	vesicle-mediated transport-related	Clathrin/coatomer adaptor, adaptin-like, N-

	protein, putative	terminal;Armadillo-type fold;AP-1/2/4 complex subunit beta;AP complex subunit beta
CNC04150	family II 2-keto-3-deoxy-D-arabino-heptulosonate aldolase, putative	DAHP synthetase, class II
CND04560	Identified spore protein 3	RmlC-like cupin domain superfamily;Cupin 2, conserved barrel
CNF00380	hypothetical protein	N/A
CNF04420	cAMP-regulated gene 1	N/A
CNF04490	Ran1-like protein kinase, putative	Protein kinase domain;Serine/threonine-protein kinase, active site;Protein kinase-like domain superfamily;Protein kinase, ATP binding site
CNG00370	expressed protein	Uncharacterized protein C3H7.08c
CNH02350	two-component-like sensor kinase	PAS domain;Signal transduction response regulator, receiver domain;Histidine kinase/HSP90-like ATPase;Signal transduction histidine kinase, dimerisation/phosphoacceptor domain;Signal transduction histidine kinase-related protein, C-terminal;CheY-like superfamily;PAS domain superfamily;Signal transduction histidine kinase, dimerisation/phosphoacceptor domain superfamily;Histidine kinase/HSP90-like ATPase superfamily
CNI02770	hypothetical protein	N/A
CNJ01750	mannose-6-phosphate isomerase, putative	Mannose-6-phosphate isomerase, type I;RmlC-like cupin domain superfamily;Mannose-6-phosphate isomerase
CNM00030	multidrug transporter, putative	Major facilitator superfamily;MFS transporter superfamily
CNM01150	hypothetical protein	N/A
CNN00050	Rds1 protein, putative	Protein Rds1
CND04630	conserved hypothetical protein	Cyanate lyase, C-terminal;Cyanate hydratase;Lambda repressor-like, DNA-binding domain superfamily;Cyanate lyase, C-terminal domain superfamily
CNC00310	Hmp1 protein, putative	N/A
CND00040	transketolase, putative	Transketolase, N-terminal;Transketolase-like, pyrimidine-binding domain;Transketolase C-terminal/Pyruvate-ferredoxin oxidoreductase domain II;Thiamin diphosphate-binding fold;Transketolase, C-terminal domain
CNG04230	hypothetical protein	N/A

CNI02270	expressed protein	N/A
CNM01270	conserved hypothetical protein	DnaJ domain;Chaperone J-domain superfamily
CNA01180	serine/threonine-protein kinase, putative	Protein kinase domain;Serine/threonine-protein kinase, active site;Protein kinase-like domain superfamily
CNB01660	expressed protein	N/A
CNC03530	proteasome subunit, beta type, 7, putative	Proteasome, subunit alpha/beta;Proteasome beta subunit, C-terminal;Nucleophile aminohydrolases, N-terminal;Proteasome subunit beta 7
CNE00350	mitogen activated protein kinase	Protein kinase domain;Mitogen-activated protein (MAP) kinase, conserved site;Serine/threonine-protein kinase, active site;Mitogen-activated protein (MAP) kinase p38-like;Protein kinase-like domain superfamily;Protein kinase, ATP binding site
CNC04990	proteasome subunit beta type 2, putative	Proteasome, subunit alpha/beta;Proteasome beta-type subunit, conserved site;Nucleophile aminohydrolases, N-terminal;Proteasome subunit beta 2
CNB04025	hypothetical protein	Zinc finger, AN1-type;AN1-like Zinc finger
CNC03730	conserved hypothetical protein	Short-chain dehydrogenase/reductase SDR;Short-chain dehydrogenase/reductase, conserved site;NAD(P)-binding domain superfamily
CNC05450	oxidoreductase, putative	Short-chain dehydrogenase/reductase SDR;NAD(P)-binding domain superfamily
CND01120	fatty acid beta-oxidation-related protein, putative	Short-chain dehydrogenase/reductase SDR;Short-chain dehydrogenase/reductase, conserved site;NAD(P)-binding domain superfamily
CND02280	oxidoreductase, putative	Short-chain dehydrogenase/reductase SDR;Short-chain dehydrogenase/reductase, conserved site;NAD(P)-binding domain superfamily
CNE03100	phosphoketolase, putative	Xylulose 5-phosphate/Fructose 6-phosphate phosphoketolase;Transketolase C-terminal/Pyruvate-ferredoxin oxidoreductase domain II;Xylulose 5-phosphate/Fructose 6-phosphate phosphoketolase, C-terminal;Xylulose 5-phosphate/Fructose 6-phosphate phosphoketolase, N-terminal;Xylulose 5-phosphate/Fructose 6-phosphate phosphoketolase, thiamine diphosphate binding site;Xylulose 5-phosphate/Fructose 6-phosphate phosphoketolase, conserved site;Thiamin diphosphate-binding fold
CNG03310	peroxisomal membrane protein, putative	Mitochondrial substrate/solute carrier;Mitochondrial carrier domain superfamily

CNK00910	tbc1 domain family protein, putative	Rab-GTPase-TBC domain;Rab-GTPase-TBC domain superfamily
CNA04970	mannitol dehydrogenase, putative	Alcohol dehydrogenase, zinc-type, conserved site;GroES-like superfamily;Alcohol dehydrogenase, N-terminal
CNA05600	catalase A, putative	Catalase haem-binding site;Catalase immune-responsive domain;Catalase core domain;Catalase, mono-functional, haem-containing;Catalase superfamily;Catalase active site;Catalase, mono-functional, haem-containing, clade 2;Class I glutamine amidotransferase-like;Large catalase, C-terminal domain
CNB05080	transaldolase, putative	Transaldolase/Fructose-6-phosphate aldolase
CNE04080	conserved hypothetical protein	Protein of unknown function DUF4449
CNL06020	catalase 4	Catalase haem-binding site;Catalase immune-responsive domain;Catalase core domain;Catalase, mono-functional, haem-containing;Catalase superfamily;Catalase active site
CNG02380	DNA replication licensing factor cdc19 (cell division control protein 19), putative	MCM domain;DNA replication licensing factor Mcm2;Nucleic acid-binding, OB-fold;P-loop containing nucleoside triphosphate hydrolase;MCM N-terminal domain;Mini-chromosome maintenance protein;MCM OB domain;MCM, AAA-lid domain
CNM01820	DNA unwinding-related protein, putative	MCM domain;Mini-chromosome maintenance complex protein 4;Nucleic acid-binding, OB-fold;Mini-chromosome maintenance, conserved site;P-loop containing nucleoside triphosphate hydrolase;MCM N-terminal domain;Mini-chromosome maintenance protein;MCM OB domain;MCM, AAA-lid domain
CNA00900	ATP dependent DNA helicase, putative	MCM domain;AAA+ ATPase domain;DNA replication licensing factor Mcm3;Nucleic acid-binding, OB-fold;P-loop containing nucleoside triphosphate hydrolase;MCM N-terminal domain;Mini-chromosome maintenance protein;MCM OB domain;MCM, AAA-lid domain
CNB05360	ATP dependent DNA helicase, putative	MCM domain;DNA replication licensing factor Mcm5;Nucleic acid-binding, OB-fold;Mini-chromosome maintenance, conserved site;P-loop containing nucleoside triphosphate hydrolase;MCM N-terminal domain;Mini-chromosome maintenance protein;MCM OB domain;MCM, AAA-lid domain
CNF03830	epsilon DNA polymerase, putative	DNA polymerase alpha/delta/epsilon, subunit B;DNA polymerase epsilon, subunit B
CNC04400	ATP-dependent DNA helicase hus2/rqh1, putative	Helicase, C-terminal;HRDC domain;DNA/RNA helicase, ATP-dependent, DEAH-box type, conserved site;DNA helicase, ATP-dependent, RecQ type;HRDC-like

		superfamily;DEAD/DEAH box helicase domain;Helicase superfamily 1/2, ATP-binding domain;RQC domain;P-loop containing nucleoside triphosphate hydrolase;ATP-dependent DNA helicase RecQ, zinc-binding domain;Winged helix DNA-binding domain superfamily
CNJ00400	DNA topoisomerase type I, putative	DNA topoisomerase, type IA;Zinc finger, CCHC-type;DNA topoisomerase, type IA, domain 2;DNA topoisomerase, type IA, DNA-binding domain;TOPRIM domain;Zinc finger, GRF-type;DNA topoisomerase, type IA, central;DNA topoisomerase, type IA, core domain;DNA topoisomerase, type IA, active site;DNA topoisomerase 3-like, TOPRIM domain;Zinc finger, CCHC-type superfamily
CNA06420	expressed protein	DM9 repeat;Domain of unknown function DUF3421
CNF01190	expressed protein	Cyclin-dependent kinase, regulatory subunit;Cyclin-dependent kinase, regulatory subunit superfamily
CNN00950	conserved hypothetical protein	Sas10 C-terminal domain
CNF04700	hypothetical protein	CCAAT-binding factor;Nucleolar complex protein 4
CNL04720	u3 small nucleolar RNA-associated protein 11, putative	Small-subunit processome, Utp11
CND05510	rRNA processing-related protein, putative	K Homology domain;Ribosomal RNA assembly KRR1;K Homology domain, type 1 superfamily;Krr1, KH1 domain
CNB03110	hypothetical protein	Nucleolar protein 14
CNJ01990	nucleolar essential protein 1, putative	Ribosomal biogenesis, methyltransferase, EMG1/NEP1;Alpha/beta knot methyltransferases
CNH02180	hypothetical protein	Down-regulated-in-metastasis protein;Armadillo-type fold
CNB00340	conserved hypothetical protein	Small-subunit processome, Utp14
CNE01200	rRNA processing-related protein, putative	S1 domain;HAT (Half-A-TPR) repeat;Suppressor of forked;Tetratricopeptide-like helical domain superfamily;Nucleic acid-binding, OB-fold;RNA-binding domain, S1
CNI01120	40S ribosomal protein S7, putative	Ribosomal protein S7e
CNE03760	conserved hypothetical protein	PIN domain;rRNA-processing protein Fcf1/Utp23;PIN-like domain superfamily
CND02860	WD-repeat protein, putative	WD40 repeat;Small-subunit processome, Utp21;Anaphase-promoting complex subunit 4, WD40 domain;WD40-repeat-containing domain superfamily

CNM00140	hypothetical protein	WD40 repeat;Quinoprotein amine dehydrogenase, beta chain-like;WD40-repeat-containing domain superfamily
CNG04380	WD repeat protein, putative	WD40 repeat;Small-subunit processome, Utp12;Quinoprotein alcohol dehydrogenase-like superfamily;WD40 repeat, conserved site;Periodic tryptophan protein 2
CNB01050	hypothetical protein	Small-subunit processome, Utp12;WD40-repeat-containing domain superfamily
CNC02190	GTP binding protein, putative	Ribosome biogenesis protein BMS1/TSR1, C-terminal;AARP2CN;P-loop containing nucleoside triphosphate hydrolase;Ribosome biogenesis protein Bms1, N-terminal;Ribosome biogenesis protein Bms1/Tsr1
CNB05270	conserved hypothetical protein	HAT (Half-A-TPR) repeat;U3 small nucleolar RNA-associated protein 6
CND03780	conserved hypothetical protein	Bystin
CNB00040	WD-repeat protein, putative	WD40 repeat;WD40-repeat-containing domain superfamily
CNB05580	small nucleolar ribonucleoprotein, putative	EF-hand domain pair;U3 small nucleolar ribonucleoprotein complex, subunit Mpp10
CNE02160	hypothetical protein	Ribosomal protein S4/S9, N-terminal;RNA-binding S4 domain;Ribosomal protein S4/S9, eukaryotic/archaeal;Ribosomal protein S4, conserved site;Ribosomal protein S4/S9
CNJ02290	conserved hypothetical protein	WD40 repeat;WD40 repeat, conserved site;WD40-repeat-containing domain superfamily;Ribosomal RNA-processing protein Rrp9-like
CND02780	40s ribosomal protein s6-b, putative	Ribosomal protein S6e;Ribosomal protein S6, eukaryotic;Ribosomal protein S6e, conserved site
CNF00670	structural constituent of ribosome, putative	Ribosomal protein S11;Ribosomal S11, conserved site
CNC05910	hypothetical protein	WD40 repeat;Quinoprotein alcohol dehydrogenase-like superfamily;Small-subunit processome, Utp13;WD40 repeat, conserved site;G-protein beta WD-40 repeat;WD40-repeat-containing domain superfamily
CNL04010	hypothetical protein	Sas10/Utp3/C1D
CNL05200	WD-repeat protein, putative	WD40 repeat;Small-subunit processome, Utp12;WD40 repeat, conserved site;G-protein beta WD-40 repeat;WD40-repeat-containing domain superfamily
CNB01450	conserved hypothetical protein	WD40 repeat;WD40-repeat-containing domain superfamily

CNA07530	57.7 kda trp-asp repeats containing protein, putative	WD40 repeat;U3 small nucleolar RNA-associated protein 15, C-terminal;WD40 repeat, conserved site;G-protein beta WD-40 repeat;WD40-repeat-containing domain superfamily
CNJ00090	u3 small nucleolar ribonucleoprotein protein imp3, putative	Ribosomal protein S4/S9, N-terminal;RNA-binding S4 domain;Ribosomal protein S4/S9
CNE03320	small nuclear ribonucleoprotein, putative	Nop domain;NOP5, N-terminal;NOSIC;Nop domain superfamily
CND02710	conserved hypothetical protein	NOL6/Upt22;Nrap protein domain 1;Nrap protein, domain 2;Nrap protein, domain 3;Nrap protein, domain 4;Nrap protein, domain 5;Nrap protein, domain 6
CNE01090	rRNA primary transcript binding protein, putative	Brix domain
CNB05450	rRNA processing-related protein, putative	NUC153;WD40-repeat-containing domain superfamily;Nucleolar protein 10/Enp2
CNK00110	methyltransferase, putative	Fibrillarin;Fibrillarin, conserved site;S-adenosyl-L-methionine-dependent methyltransferase
CNG00800	conserved hypothetical protein	Ribosomal RNA-processing protein 7, C-terminal domain;RNA-binding domain superfamily;Rrp7, RRM-like N-terminal domain
CNG01210	conserved hypothetical protein	Digestive organ expansion factor, predicted
CNF04820	hypothetical protein	Ribosomal protein L1-like;Ribosomal protein L1/ribosomal biogenesis protein
CNK03230	40s ribosomal protein s5-1, putative	Ribosomal protein S5/S7;Ribosomal protein S5/S7, eukaryotic/archaeal;Ribosomal protein S7, conserved site;Ribosomal protein S7 domain;Ribosomal protein S7 domain superfamily
CNB04730	nam9 protein, mitochondrial precursor, putative	RNA-binding S4 domain;Ribosomal protein S4, bacterial-type;Ribosomal protein S4/S9
CNI02150	conserved hypothetical protein	Ribosomal protein S11
CNN01910	ribosomal protein S12, putative	Ribosomal protein S12, bacterial-type;Ribosomal protein S12/S23;Nucleic acid-binding, OB-fold
CNA00950	mRNA export factor elf1, putative	Chromo/chromo shadow domain;ABC transporter-like;AAA+ ATPase domain;Armadillo-type fold;Chromo-like domain superfamily;Chromo domain;P-loop containing nucleoside triphosphate hydrolase
CNC04540	30S ribosomal protein S17, putative	Ribosomal protein S17/S11;Nucleic acid-binding, OB-fold
CNC01900	structural constituent of ribosome, putative	Ribosomal protein S15;Ribosomal protein S15, bacterial-type;S15/NS1, RNA-binding

CNN01880	nucleolar GTP-binding protein 1, putative	GTP binding domain;Nucleolar GTP-binding protein 1, Rossman-fold domain;NOG, C-terminal;Nucleolar GTP-binding protein 1;P-loop containing nucleoside triphosphate hydrolase;NOG1, N-terminal helical domain
CND03120	conserved hypothetical protein	Brix domain;Ribosome biogenesis protein Rpf2
CNH01320	conserved hypothetical protein	Nucleolar complex protein 2
CNB02830	ribosomal protein, putative	Ribosomal protein L10P;Ribosome assembly factor Mrt4;60S ribosomal protein L10P, insertion domain;Ribosomal protein L10-like domain superfamily
CNC05560	ribosomal large subunit biogenesis-related protein, putative	RNA recognition motif domain;RNA-binding domain superfamily
CNC02820	conserved hypothetical protein	Brix domain
CNF02140	30s ribosomal protein s5, putative	Ribosomal protein S5, C-terminal;Ribosomal protein S5, N-terminal;Ribosomal protein S5 domain 2-type fold
CNB02740	ribosomal protein L4, putative	Ribosomal protein L4/L1e;50S ribosomal protein uL4;Ribosomal protein L4 domain superfamily
CNB05550	cytosolic large ribosomal subunit protein, putative	PUA domain;UPF0113, PUA domain;PUA-like superfamily;Ribosome biogenesis factor, NIP7;UPF0113, pre-PUA domain
CNB02220	nucleolus protein, putative	SAM-dependent methyltransferase RsmB/NOP2-type;Nop2p;Bacterial Fmu (Sun)/eukaryotic nucleolar NOL1/Nop2p, conserved site;RNA (C5-cytosine) methyltransferase;RNA (C5-cytosine) methyltransferase, NOP2;S-adenosyl-L-methionine-dependent methyltransferase;Ribosomal RNA small subunit methyltransferase F, N-terminal
CND03550	rRNA processing-related protein, putative	Eukaryotic rRNA processing
CND02420	elongation factor 3	Chromo/chromo shadow domain;ABC transporter-like;AAA+ ATPase domain;Armadillo-type fold;ABC transporter, conserved site;P-loop containing nucleoside triphosphate hydrolase;TOG domain
CNE04660	hypothetical protein	N/A
CNE02690	ribosomal large subunit assembly and maintenance-related protein, putative	Ribosomal protein L3;Translation protein, beta-barrel domain superfamily;Ribosomal protein L3, conserved site;Ribosomal protein L3, bacterial/organelle-type
CNE01960	ribosomal protein L2, putative	Ribosomal protein L2;Translation protein SH3-like domain superfamily;Nucleic acid-binding, OB-fold;Ribosomal Proteins L2, RNA binding domain;Ribosomal protein L2, C-

		terminal
CNE04980	NTL02AT1906 50S ribosomal protein L22, putative	Ribosomal protein L22/L17;Ribosomal protein L22, bacterial/chloroplast-type;Ribosomal protein L22/L17 superfamily
CNA05935	hypothetical protein	WD40 repeat;Sof1-like protein;WD40-repeat-containing domain superfamily
**Nodes present in Cna1 and Crz1 PPINs**		
**Gene ID (*C. neoformans* JEC21)**	**FungiDB product description**	**Interpro description according to FungiDB**
CNI01170	thiamine pyrophosphokinase, putative	Thiamin pyrophosphokinase;Thiamin pyrophosphokinase, catalytic domain;Thiamin pyrophosphokinase, thiamin-binding domain;Thiamin pyrophosphokinase, eukaryotic;Thiamin pyrophosphokinase, thiamin-binding domain superfamily;Thiamin pyrophosphokinase, catalytic domain superfamily
CNG03470	hypothetical protein	Arv1 protein
CNF01610	putative chitin synthase	Cytochrome b5-like heme/steroid binding domain;Myosin head, motor domain;DEK, C-terminal;P-loop containing nucleoside triphosphate hydrolase;Nucleotide-diphospho-sugar transferases;Class XVII myosin, motor domain;Cytochrome b5-like heme/steroid binding domain superfamily
CNH01200	conserved hypothetical protein	Concanavalin A-like lectin/glucanase domain superfamily
CNG02640	conserved hypothetical protein	YTH domain
CND02140	hypothetical protein	Zinc finger, RING-type
CNN00660	glucan 1,3 beta-glucosidase protein putative	Glycoside hydrolase family 17;Glycoside hydrolase superfamily
CND06130	putative beta-glucan synthase	Beta-glucan synthesis-associated, Skn1;Concanavalin A-like lectin/glucanase domain superfamily
CNG04125	hypothetical protein	N/A
CNF00830	conserved hypothetical protein	SH3 domain;Ysc84 actin-binding domain;SH3YL1-like, SYLF domain;SH3-like domain superfamily
CNN00410	cytoplasm protein, putative	SNARE associated Golgi protein
CNF00610	alpha-glucosidase, putative	Glycosyl hydrolase, family 13, catalytic domain;Glycoside hydrolase superfamily

CNM00090	glycoprotein, putative	N/A
CNL06430	hypothetical protein	Sec1-like protein;Sec1-like superfamily
CNE00400	expressed protein	N/A
CNA06460	hypothetical protein	N/A
CNF02420	glyoxal oxidase precursor, putative	Glyoxal oxidase, N-terminal;Galactose oxidase/kelch, beta-propeller;Immunoglobulin E-set;Galactose oxidase-like, Early set domain
CNA05710	conserved hypothetical protein	Ricin B, lectin domain;Ricin B-like lectins
CNH01730	conserved hypothetical protein	Aldo/keto reductase, conserved site;Aldo/keto reductase;NADP-dependent oxidoreductase domain;NADP-dependent oxidoreductase domain superfamily
CNG02110	specific transcriptional repressor, putative	High mobility group box domain;High mobility group box domain superfamily
CNA05300	putative chitin synthase	Cytochrome b5-like heme/steroid binding domain;Chitin synthase;DEK, C-terminal;Nucleotide-diphospho-sugar transferases;Cytochrome b5-like heme/steroid binding domain superfamily
CNN02240	phospholipid metabolism-related protein, putative	Phosphatidic acid phosphatase type 2/haloperoxidase;Phosphatidic acid phosphatase type 2/haloperoxidase superfamily;Phospholipid phosphatase
CNG01720	endochitinase	Glycoside hydrolase family 18, catalytic domain;Glycosyl hydrolases family 18 (GH18) active site;Chitinase II;Glycoside hydrolase superfamily;Chitinase insertion domain superfamily
CNH02560	syntaxin, putative	Target SNARE coiled-coil homology domain;Syntaxin, N-terminal domain;SNARE
CNA02870	hypothetical protein	N/A
CNE05040	glyoxal oxidase precursor, putative	Glyoxal oxidase, N-terminal;Galactose oxidase/kelch, beta-propeller;Immunoglobulin E-set;Galactose oxidase-like, Early set domain
CNA07540	immunoreactive mannoprotein	N/A
CNI01470	vesicular-fusion protein, putative	NSF attachment protein;Tetratricopeptide-like helical domain superfamily
CNE03810	expressed protein	N/A

CNA03960	glyoxal oxidase precursor, putative	Glyoxal oxidase, N-terminal;Galactose oxidase/kelch, beta-propeller;Immunoglobulin E-set;Galactose oxidase-like, Early set domain
CNE00530	hypothetical protein	N/A
CNF00590	hypothetical protein	N/A
CNA06270	hypothetical protein	Small subunit of serine palmitoyltransferase-like
CNB01020	mandelate racemase/muconate lactonizing enzyme, putative	Mandelate racemase/muconate lactonizing enzyme, N-terminal domain;Mandelate racemase/muconate lactonizing enzyme, C-terminal;Mandelate racemase/muconate lactonizing enzyme, conserved site;Enolase-like, N-terminal;Enolase C-terminal domain-like;L-fuconate dehydratase;Enolase-like, C-terminal domain superfamily
CND03490	chitin-deacetylase	NodB homology domain;Glycoside hydrolase/deacetylase, beta/alpha-barrel
CNK01210	hypothetical protein	FYVE zinc finger;Zinc finger, FYVE/PHD-type
CNG03500	hypothetical protein	Kre9/Knh1 family
CNF01760	hypothetical protein	Glycoside hydrolase, family 5;Glycoside hydrolase superfamily
CNH03510	v-SNARE, putative	Synaptobrevin;Synaptobrevin/Vesicle-associated membrane protein
CNJ00590	glycogen (starch) synthase, putative	Glycogen synthase
CNL04840	exo-beta-1,3-glucanase	Glycoside hydrolase, family 5;Glycoside hydrolase superfamily
CNN02260	1,3-beta-glucanosyltransferase, putative	Glucanosyltransferase;X8 domain;Glycoside hydrolase superfamily
CNA00180	vacuolar calcium exchanger	Sodium/calcium exchanger membrane region
CNA00820	conserved expressed protein	N/A
CNG01240	laccase precursor, putative	Multicopper oxidase, type 1;Cupredoxin;Multicopper oxidase, type 2;Multicopper oxidase, type 3
CNG01250	Laccase 2	Multicopper oxidase, type 1;Cupredoxin;Multicopper oxidase, type 2;Multicopper oxidase, type 3
CNI00320	glyceraldehyde 3-phosphate dehydrogenase, putative	Glyceraldehyde-3-phosphate dehydrogenase, type I;Glyceraldehyde 3-phosphate dehydrogenase, NAD(P) binding domain;Glyceraldehyde 3-phosphate dehydrogenase, catalytic domain;Glyceraldehyde 3-

		phosphate dehydrogenase, active site;Glyceraldehyde/Erythrose phosphate dehydrogenase family;NAD(P)-binding domain superfamily
CNJ01390	expressed protein	Extracellular membrane protein, CFEM domain
CNJ01130	conserved hypothetical protein	N/A
CNM01670	conserved hypothetical protein	Helicase, C-terminal;Helicase/UvrB, N-terminal;Helicase superfamily 1/2, ATP-binding domain;P-loop containing nucleoside triphosphate hydrolase;FANCM/Mph1-like
CNJ03260	hypothetical protein	N/A
CNI00790	hypothetical protein	N/A
**Nodes present in Pmc1 and Crz1 PPINs**		
**Gene ID (*C. neoformans* JEC21)**	**FungiDB product description**	**Interpro description according to FungiDB**
CNJ02900	hypothetical protein	Transglycosylase SLT domain 1;Lysozyme-like domain superfamily
CNN01830	expressed protein	N/A
CND02460	hypothetical protein	N/A
CNC05750	expressed protein	UPF0658 Golgi apparatus membrane protein
CNH03250	hypothetical protein	N/A
CNC06440	inositol-3-phosphate synthase, putative	Myo-inositol-1-phosphate synthase;Myo-inositol-1-phosphate synthase, GAPDH-like;NAD(P)-binding domain superfamily
CND01230	meiotic recombination-related protein, putative	Putative stress-responsive protein
CNC07140	expressed protein	Glycosyltransferase 61
CNE03240	putative chitin synthase	Fungal chitin synthase;Chitin synthase;Chitin synthase N-terminal;Nucleotide-diphospho-sugar transferases
CNI01670	peptidase, putative	Aspartic peptidase A1 family;Aspartic peptidase domain superfamily;Peptidase family A1 domain;Pepsin-like domain
CNC03930	hypothetical protein	Zn(2)-C6 fungal-type DNA-binding domain;Transcription factor domain, fungi;Zn(2)-C6 fungal-type DNA-binding domain superfamily

CNG04480	trehalose synthase, putative	Glycosyl transferase, family 1
CNA01500	alternative oxidase 1	Alternative oxidase
CNC04360	vacuolar membrane protein, putative	Store-operated calcium entry regulator STIMATE/YPL162C
CNC04640	expressed protein	N/A
CNA02750	cytoplasm protein, putative	Tetratricopeptide repeat 1;Tetratricopeptide-like helical domain superfamily;Tetratricopeptide repeat;SGTA, homodimerisation domain
CNH02490	cytoplasm protein, putative	Glycoside hydrolase, family 5;Glycoside hydrolase superfamily;Glycoside hydrolase family 5 C-terminal domain
CNC04960	Ras-like GTP-binding protein, putative	Small GTPase;Small GTP-binding protein domain;P-loop containing nucleoside triphosphate hydrolase
CNB03660	RHEB small monomeric GTPase, putative	Small GTPase;Small GTP-binding protein domain;P-loop containing nucleoside triphosphate hydrolase
CNJ01660	expressed protein	N/A
CNN00760	pr4/barwin domain protein	RlpA-like domain superfamily
CNK02140	conserved hypothetical protein	Membrane-associated, eicosanoid/glutathione metabolism (MAPEG) protein;Membrane associated eicosanoid/glutathione metabolism-like domain superfamily
CNB05330	serine/threonine protein phosphatase 5 phosphatase, putative	Tetratricopeptide-like helical domain superfamily;Tetratricopeptide repeat
CNK00760	conserved hypothetical protein	von Willebrand factor A-like domain superfamily
CNK03430	protein of unknown function	Phosphatidylethanolamine-binding protein;Phosphatidylethanolamine-binding protein, eukaryotic;PEBP-like superfamily
CNC02870	expressed protein	N/A
CNC05510	hypothetical protein	Zinc finger, PARP-type
CNC00045	chitin synthase, putative	N/A
CNC05770	expressed protein	NADH:ubiquinone oxidoreductase intermediate-associated protein 30;Complex I intermediate-associated protein 30, mitochondrial
CND03060	ribosomal chaperone, putative	Ubiquitin-like domain;Ribosomal protein S27a;Zinc-binding ribosomal protein;Ubiquitin conserved

		site;Ubiquitin domain;Ubiquitin-like domain superfamily
CNB01010	conserved hypothetical protein	Ubiquitin-like domain;Heat shock chaperonin-binding;UBA-like superfamily;Ubiquilin;Ubiquitin-associated domain;Ubiquitin domain;Ubiquitin-like domain superfamily
CNK02620	ubiquitin precursor	Ubiquitin-like domain;Ubiquitin conserved site;Ubiquitin domain;Ubiquitin-like domain superfamily
CNC05980	nucleus protein, putative	Zinc finger, AN1-type;AN1-like Zinc finger
CNC04440	ubiquitin-like protein	Ubiquitin-like domain;Ubiquitin conserved site;Ubiquitin domain;Ubiquitin-like domain superfamily;Nedd8-like ubiquitin
CNK02170	conserved hypothetical protein	Ubiquitin-like domain;Ubiquitin domain;Ubiquitin-like domain superfamily
CNA07500	uv excision repair protein rhp23, putative	Ubiquitin-like domain;UV excision repair protein Rad23;Heat shock chaperonin-binding;UBA-like superfamily;XPC-binding domain;Ubiquitin-associated domain;Ubiquitin-like domain superfamily;XPC-binding domain superfamily

N/A - Not Available
