# Supplementary material for: Integrative Transcriptomic and Systems Biology Analyses Identify TCB1 as a Calcium-Responsive Gene in Cryptococcus neoformans
Source: Microorganisms. 2026 Jan 7;14(1):122. doi: 10.3390/microorganisms14010122 (PMC12843964; doi:10.3390/microorganisms14010122)
Supplement: Supplementary file 1 [file microorganisms-14-00122-s001.zip › Supplementary Table S7.pdf]

Supplementary Table S7. Literature microarray and RNA-seq data evidence of possible *TCB1* participation in *C. neoformans* virulence and survival.

Expression	Log_2FC	P-value	Comparison	Reference	Reference title
**Microarray**					
Upregulated	1.8984	1.22E-05	Internalized by *Acanthamoeba castellanii* x PYG medium	Derengowski et al., 2013	The Transcriptional Response of *Cryptococcus neoformans* to Ingestion by *Acanthamoeba castellanii* and Macrophages Provides Insights into the Evolutionary Adaptation to the Mammalian Host
**RNA-seq**					
Upregulated	2.9250	0.001	*in vivo* human CSF *x* YPD	Chen et al., 2014	The *Cryptococcus neoformans* transcriptome at the site of human meningitis
Upregulated	0.5753	1.55E-04	Titan-like cells grown in TCM x regular cells grown in TCM after 7hours	Trevijano-Contador et al. 2018	*Cryptococcus neoformans* can form titan-like cells *in vitro* in response to multiple signals
Upregulated	1.98	2.00E-02	Titan-like cells grown in TCM x regular cells grown in TCM after 18 hours		
Downregulated	-3.0472	2.75E-38	H99 at 37ºC total mRNA x *ccr4*Δ at 37ºC total mRNA	Bloom et al. 2019	Thermotolerance in the pathogen *Cryptococcus neoformans* is linked to antigen masking via mRNA decay-dependent reprogramming
Upregulated	3.5951	1.84E-47	*ccr4*Δ at 30ºC total mRNA X *ccr4*Δ at 37ºC total mRNA		
Upregulated	3.4737	3.28E-44	*ccr4*Δ at 37ºC mRNA polysomal mRNA X *ccr4*Δ at 30ºC polysomal mRNA		
Downregulated	-3.0801	2.42E-38	H99 at 37ºC polysomal mRNA x *ccr4*Δ at 37ºC polysomal mRNA		
Upregulated	1.1266	0.006	H99 x *ada2*Δ mutant strain grown in Littman's medium	Jang et al. 2022	Unraveling Capsule Biosynthesis and Signaling Networks in *Cryptococcus neoformans*
Upregulated	2.8376	3.11E-08	wild-type H99 x *bzp4*Δ mutant strain grown in Littman's medium		

Log_2FC: $Log_2FoldChange$; TCM: Titan Cell Medium; CSF: Cerebrospinal Fluid.
