# Supplementary material for: Integrative Transcriptomic and Systems Biology Analyses Identify TCB1 as a Calcium-Responsive Gene in Cryptococcus neoformans
Source: Microorganisms. 2026 Jan 7;14(1):122. doi: 10.3390/microorganisms14010122 (PMC12843964; doi:10.3390/microorganisms14010122)
Supplement: Supplementary file 1 [file microorganisms-14-00122-s001.zip › Supplementary Table S8.pdf]

Supplementary Table S8. FIMO results for TFBS in *TCB1* promoter region.

Motif ID	Motif Sequence	Promoter	Start	Stop	Strad	Score	p-value	q-value	Matched Sequence
Pdr802_3	5'-GA[A/C]GA-GA-3'	*TCB1*	539	546	+	12.6242	2.41e-05	0.0446	GACGACGA
Pdr802_2	5'-GA[C/G/T]GA[C/T]GA-3'	*TCB1*	539	546	+	13.0121	2.41e-05	0.0446	GACGACGA
Crz1_1	5'-[A/G]CACAGC[A/C]AC-3'	*TCB1*	394	403	+	11.8788	6.91e-05	0.0383	ACACAGCCAA
Pdr802_4	5'-C[C/T]TCC[A/G/T]CC-3'	*TCB1*	920	927	+	9.58788	7.01e-05	0.0219	CCTCCCCC
